# Virtual Reality in Neurorehabilitation: An Umbrella Review of Meta-Analyses

**DOI:** 10.3390/jcm10071478

**Published:** 2021-04-02

**Authors:** Alexandra Voinescu, Jie Sui, Danaë Stanton Fraser

**Affiliations:** 1 Department of Psychology, University of Bath, 10 West, Claverton Down, Bath BA2 7AY, UK; D.StantonFraser@bath.ac.uk; 2 The School of Psychology, King’s College, University of Aberdeen, Aberdeen AB24 3FX, UK; jie.sui@abdn.ac.uk

**Keywords:** neurological disorders, stroke, traumatic brain injury, cerebral palsy, rehabilitation, virtual reality

## Abstract

Neurological disorders are a leading cause of death and disability worldwide. Can virtual reality (VR) based intervention, a novel technology-driven change of paradigm in rehabilitation, reduce impairments, activity limitations, and participation restrictions? This question is directly addressed here for the first time using an umbrella review that assessed the effectiveness and quality of evidence of VR interventions in the physical and cognitive rehabilitation of patients with stroke, traumatic brain injury and cerebral palsy, identified factors that can enhance rehabilitation outcomes and addressed safety concerns. Forty-one meta-analyses were included. The data synthesis found mostly low- or very low-quality evidence that supports the effectiveness of VR interventions. Only a limited number of comparisons were rated as having moderate and high quality of evidence, but overall, results highlight potential benefits of VR for improving the ambulation function of children with cerebral palsy, mobility, balance, upper limb function, and body structure/function and activity of people with stroke, and upper limb function of people with acquired brain injury. Customization of VR systems is one important factor linked with improved outcomes. Most studies do not address safety concerns, as only nine reviews reported adverse effects. The results provide critical recommendations for the design and implementation of future VR programs, trials and systematic reviews, including the need for high quality randomized controlled trials to test principles and mechanisms, in primary studies and in meta-analyses, in order to formulate evidence-based guidelines for designing VR-based rehabilitation interventions.

## 1. Introduction

Neurological disorders are a leading cause of death and disability worldwide with estimated annual costs of €266 billion in Europe [1]. Consequences of disabilities caused by neurological disorders can be reduced by rehabilitation programs in addition to promotion, prevention and treatment [2].

New promising interventions to improve rehabilitation outcomes such as virtual reality (VR)-based interventions have been developed. Using various technical devices (e.g., head-mounted displays, desktop computers, video capture systems, tracking systems, motion-sensing gloves), VR delivers realistic experiences by creating virtual environments (VEs) that closely resemble everyday environments [3]. Common examples of VR programs with promising results for rehabilitation of patients with non-progressive neurological conditions such as stroke or cerebral palsy (CP) are VR-based treadmill training for lower extremity [4], reaching and grasping of virtual objects exercises for the upper extremity [5], and even playing games and performing various activities using commercially available serious games platforms for upper and lower limb function: Nintendo WII or Xbox Kinect [6,7].

Significant improvements in rehabilitation outcomes for patients who underwent VR-based interventions may be explained by their ability to offer meaningful and realistic experiences, thus accommodating principles of rehabilitation [8]. Learning improves if the tasks are meaningful, specific and repetitive and if the task difficulty is increased over time [8,9]. In VR, the number of stimuli and the difficulty of tasks can be adjusted to the needs and possibilities of the patients while maintaining stimulus control and consistency [3,10,11]. Feedback is a key component for motor learning and facilitates quick self-correction [12,13] and VR systems can provide real-time, strategic and goal-directed feedback [3,11]. VR can also be viewed as a medium which offers environmental enrichment. Previous research in animal and human studies showed the positive effect of enriched environments on motor and cognitive performance [14,15].

Recently, there has been an increase in VR-based interventions for rehabilitation with mixed results. To better understand the impact of VR-based interventions on neurological rehabilitation, the first objective of this umbrella review was to summarise the evidence of published meta-analyses regarding the effects of VR-based interventions for improving physical and cognitive functions of patients with stroke, traumatic brain injury (TBI) and CP and assess the quality of the evidence. Three past umbrella reviews reviewed the effectiveness of various interventions, including VR, on upper limb outcome [16], balance outcomes [17] and activities of daily living [18] in stroke with mixed results. Pollock [16] identified moderate quality of evidence in favour of VR; Arienti [17] reported mixed results with quality of evidence ranging from low to high quality; and García-Rudolph [18] reported small to moderate effects for VR interventions, but the quality of evidence was not graded. To our best knowledge, no umbrella review has investigated the effects of VR for CP or TBI. This is the first umbrella review to comprehensively assess the effectiveness of VR-based interventions on multiple physical and cognitive domains and identify factors associated with treatment effects, for patients with stroke, CP and TBI.

Because of the increased heterogeneity of VR platforms and interventions for rehabilitation which may facilitate change via different underlying mechanisms, our second objective was to assess the effects of factors that can impact rehabilitation. Using results from subgroup comparisons, we defined two broad classes of moderator variables referring to VR technology-related variables (e.g., immersion and presence, customization of VR systems), sample and study methodology (e.g., age, clinical diagnosis, nature of the control groups used as comparators). We were also interested in safety issues related to the use of VR especially with vulnerable populations, particularly regarding adverse effects of VR exposure [19,20]. Thirdly, we assessed whether VR is safe by quantifying the number and severity of adverse effects reported in the reviews.

## 2. Methods

This umbrella review was conducted in accordance with the Cochrane guidelines for overview of reviews [21] and the Joanna Briggs Institute guidelines for umbrella reviews [22]. The protocol was registered on the Open Science Framework (OSF) (registration number: osf.io/w6hs8). No ethical approval was needed as we used data from published studies.

### 2.1. Eligibility Criteria

The eligibility criteria was: (a) studies that employed a meta-analytic method; (b) participants with a clinical diagnosis of stroke, TBI, CP and acquired brain injury (ABI), caused by either stroke, TBI or CP; (c) VR-based interventions for rehabilitation of physical and/or cognitive abilities; (d) physical functioning (e.g., upper limb function, balance, gait, motor skills) and/or cognitive functioning outcomes (e.g., attention, memory, executive functioning). We included meta-analytical reviews which used a wide range of VR platforms, such as: head-mounted displays (HMDs), television (TV) screens, desktop computers, video capture systems, tracking systems, headphones, motion-sensing gloves, joysticks, keyboards, including commercial computer games platforms such as the Nintendo WII. In addition, the meta-analysis should have employed appropriate methods. We chose to include only meta-analyses instead of having a broader approach and including systematic reviews without meta-analytical data. The reason for this is the fact that meta-analytical studies offer an effect estimate which would facilitate data synthesis, but this was not the case for systematic reviews. As recommended in the Cochrane guidelines [21] we reported our results and statistical summaries by outcomes.

We included peer reviewed articles, conference proceedings, chapters, dissertation thesis and grey literature. We restricted our focus to English language publications to ensure we had an excellent understanding of methods and data analysis reported by authors. In order to increase power and reduce selection bias, we included meta-analyses which performed subgroup analyses and reported pooled effect sizes for our variables of interest and outcomes.

### 2.2. Search Strategy

A comprehensive search strategy was employed and performed by two review authors to identify potentially relevant records. We searched the following databases through February, 2020 and updated in December, 2020: the Cochrane Database of Systematic Reviews, PsycINFO, EMBASE, PubMed, SCOPUS, ISI Web of Science, Database of Abstracts of Reviews of Effects, Physiotherapy Evidence Database, ACM Digital Library, IEEE Xplore Digital Library, ProQuest Dissertations & Theses A&I, Open Access Theses and Dissertations, EThOS e-theses online service. We searched for the following terms in the publication’s title, abstract, and keywords: (“virtual reality” OR “vr” OR “virtual environment” OR game OR immersive) AND (rehab* OR improv* OR train* OR intervention OR treat* OR expos* OR remediat*) AND (meta-analy* OR review). The search string was modified appropriately for the various databases and an example can be found in the Supplementary Materials. We also searched the references from the most recent systematic reviews and meta-analyses.

### 2.3. Data Collection and Analysis

#### 2.3.1. Selection of Meta-Analysis Process

Two reviewers independently screened the titles and abstracts. All records deemed relevant were retrieved in full text and were reviewed by two reviewers in order to determine whether they met the selection criteria stated previously. Any disagreements were resolved through discussion with a third reviewer.

#### 2.3.2. Data Extraction and Management

Data were extracted independently by two reviewers using a predefined extraction form. Any concerns were discussed with a third reviewer. Where any information from the reviews was unclear or missing, we contacted the review authors. Two attempts were made. We extracted: (a) meta-analysis identification data (e.g., authors, year of publication and county of origin); (b) population characteristics (e.g., age and diagnosis); (c) intervention and control group characteristics (e.g., type of intervention, VR platform, intervention time); (d) review characteristics (e.g., trial design, number of primary studies and number of participants, number of participants per intervention and control group); (e) statistical summaries (e.g., outcomes and effect measure with 95% confidence intervals, *p* values and heterogeneity); (f) apriori moderators (e.g., age, immersion and presence, type of VR platform).

The outcomes were categorized as: (a) lower limb activity (e.g., mobility, ambulation function, gait, walking speed); (b) balance and postural control; (c) upper limb, arm function and activity (e.g., grip strength, arm function, improvement of motor impairment and motor function, arm-hand activities); (d) activity limitation (e.g., activities of daily living, global function, independence); (e) ICF WHO Framework outcomes (e.g., participation, body structure and function, activity); (f) motor function; (g) cognitive functioning (e.g., overall cognition).

#### 2.3.3. Quality of Included Reviews

One review author performed quality assessment of all included meta-analysis and another two reviewers performed the assessment of a random sample of included studies and obtained good agreement. We used the AMSTAR 2 [23] to assess the methodological quality of the included reviews (see Supplementary Materials). Risk of bias (ROB) was reported as assessed by the original review authors. Quality of evidence for each outcome was judged using a modified version for systematic reviews of the GRADE approach [24] (described in the Supplementary Materials).

#### 2.3.4. Overlapping of Studies

We calculated the corrected covered area (CCA) to account for overlapping of studies [25] (Supplementary Materials contains a spreadsheet used to calculate CCA).

#### 2.3.5. Data Synthesis

We produced a narrative description and synthesis of the reviews. We organized the review findings by outcomes and reported all the comparisons that were provided by review authors. For each comparison, we extracted the effect size and the 95% CI (e.g., standardized mean differences, mean differences) and heterogeneity (I*2*) as reported by review authors. To assess the magnitude of the effect, for standardized mean difference and Hedge’s g coefficients we used Cohen’s metrics where a value of between 0.20 and 0.50 indicates a small effect, one between 0.50 and 0.80 indicates a medium effect, while a value larger than 0.80 indicates a large effect size [26]. For mean differences and weighted mean differences, we used the review authors judgements about the magnitude of results because they were in the best position to understand and evaluate the scale results and cut-off scores, given their familiarity with study-level data. For odds ratio, no estimation of the magnitude of the effect was employed because each odds ratio estimates was explained by different variables and each statistical model had a different arbitrary scaling factor [27]. We extracted I*2* as a measure of heterogeneity and interpreted the heterogeneity based on the criteria provided by the Cochrane Handbook. I2 values ranging from 0 to 50% correspond to low and not important heterogeneity, values ranging from 50% to 75% correspond to moderate heterogeneity and values above 75% indicate substantial heterogeneity [28].

For moderator effects, we employed a similar approach of data extraction and reporting as we did for the overall effects. To address safety concerns, we extracted available data and reported the number of primary studies and meta-analyses that reported adverse effects and their magnitude and/or severity. Further details concerning moderator effects data synthesis are available in Supplementary Materials.

## 3. Results

**Figure 1:**
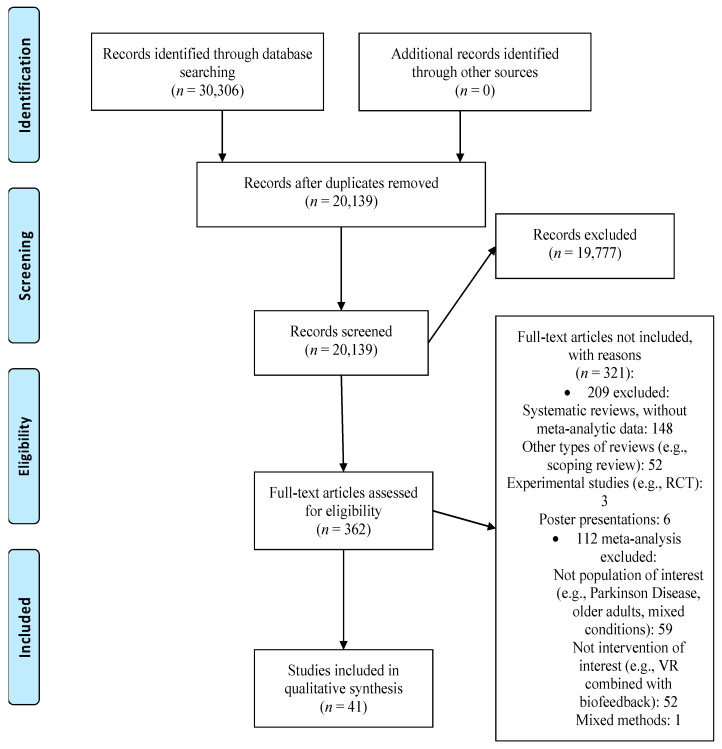
PRISMA flow diagram. RCT: randomized controlled trial, VR: virtual reality.

Our search generated 30,306 records. We excluded 10,167 duplicates and screened 20,139 records. After screening the title and abstract 19,777 articles were excluded and the full text of 362 papers was assessed. We excluded 321 records because they focused on other types of interventions and populations. Thus, 41 meta-analyses met our inclusion criteria and were included in the umbrella review (see Figure 1 for the PRISMA flowchart [29]; Supplementary Materials, Table S1 contains a list of excluded studies with reasons).

### 3.1. Description and Methodological Quality of Included Reviews

#### 3.1.1. Study Characteristics

Forty-one reviews with meta-analytical results were included in our umbrella review. Characteristics of the study, type of patient population, intervention and control conditions, type of VR platform used, and outcomes can be found in Table 1.

#### 3.1.2. Participants

Thirty-two reviews included patients with stroke, six had samples of children with CP [34,36,45,48,55,59,65,67,68] and three had patients with ABI, including stroke and TBI [33,57,61].

#### 3.1.3. Intervention Characteristics

All reviews focused on VR-based interventions, either delivered as standalone interventions or in combination with conventional therapy. Twenty reviews (49%) included both types of interventions in the analyses, six did not specify if VR interventions were delivered alone or in combination with conventional therapy (15%), eight included only VR interventions without conventional therapy (29%) and four reviews included VR with conventional therapy (10%). Three reviews (7%) investigated the moderator effects of VR-based interventions delivered alone versus VR-based interventions delivered in combination with conventional therapy [38,41,50].

#### 3.1.4. Control Group Characteristics

To eliminate more sources of bias from influencing the effect of the VR-based intervention, most of the reviews (37 reviews, 90%) computed pooled effect sizes from primary studies with adequate experimental designs and adequate control groups (e.g., RCTs or quasi-RCTs) allowing comparison of effects of VR-based interventions with control conditions (passive and active conditions), the remaining four reviews included in their analysis those studies with a pre-test post-test design (10%) [34,40,45,60]. Many control interventions were active conditions (e.g., conventional therapy) (19 reviews, 51%), but a considerable number of reviews included comparisons based on heterogeneous control groups (conventional therapy and passive control groups such as waiting list included in the same analysis) (13 reviews, 35%). For some comparisons, the control group type was not specified (3 reviews, 8%) (see Supplementary Materials for Tables S4–S10).

#### 3.1.5. Quality of Included Reviews

According to AMSTAR 2 [23] concerns regarding the methodological quality of the reviews were mainly caused by failure to: (a) report on the sources of funding for primary studies (95%); (b) perform a comprehensive literature search (93%); (c) justify the inclusion of RCTs or non-RCTs (78%); (d) to account for ROB in individual studies when interpreting and discussing results (68%) (Supplementary Materials, Table S2). Forty out of 41 reviews assessed risk of bias. Most reviews used the Physiotherapy Evidence Database (PEDro) Scale (21 reviews, 52%) and Cochrane’s “Risk of bias” tool (15 reviews, 36%). One used the Jadad scale (3%), one used the Joanna Briggs Institute Critical Appraisal tool for RCTs (3%), one used Downs-Black rating scale items (3%) and one used an adapted scoring protocol (3%). Major concerns in relation to ROB were related to performance bias as all reviews (88%) that assessed blinding of participants and personnel included primary studies at high or unclear risk of performance bias (more than 75% of the primary studies reported high or unclear risk of performance bias). Results of GRADE assessment indicated that for immediate follow-up assessment, most evidence was of very low (55 effects out of 147 effects; 37%) and low quality (76 effects out of 147 effects, 52%). Only 14 effects were of moderate quality (10%) and 2 of high quality (1%) (detailed in Supplementary Materials, Tables S4–S10).

#### 3.1.6. Overlapping of Studies

Using the formula provided by Pieper [25] we obtained a value of CCA of 0.042 which indicates a slight overlap of studies.

### 3.2. Intervention Effects and Quality of Evidence

Our first goal was to investigate the effectiveness and quality of the evidence for VR-based interventions on physical and cognitive outcomes of patients with stroke, TBI and CP.

#### 3.2.1. Intervention Effects for Lower Limb Activity

Nineteen meta-analyses assessed the effectiveness of VR interventions at immediate follow-up for lower limb activity compared with conventional therapy or no intervention. Sixteen focused on stroke and three on CP. For CP all three reviews [36,45,65] reported significant improvements in favour of VR with moderate to large effects and very low to moderate quality of evidence. Their analysis [36] included only RCTs and identified moderate heterogeneity. [65] focused only on RCTs but had substantial heterogeneity in results. [45] used a pre-post-test design with low heterogeneity in results. In the case of people with stroke, ten reviews [37,40,43,46,47,51,52,53,58,62] identified low to large significant effects in favour of VR with very low to moderate quality of evidence. Nine reviews included only RCTs in their analysis, but [40] included studies with a pre-post-test design. Heterogeneity was low for most comparisons. Four reviews which included only RCTs and used Timed Up and Go Test (TUG) as an outcome measure of mobility reported improvements for VR groups with effects ranging in magnitude from low to moderate and quality ranging from very low to moderate [42,47,52,62]. On the contrary, two reviews, one that included only RCTs [13] and one with pre-post-test design studies [44] did not identify benefits of using VR on TUG but with low quality of evidence. Heterogeneity was low. Two reviews based on RCTs analysed if effects remain at follow-up (up to 3 months) for people with stroke [38,46]. Significant effects in favour of VR but with low magnitude were reported for walking speed and gait velocity with low and very low quality of evidence. No significant improvements were obtained for functional mobility but with very low quality of evidence.

#### 3.2.2. Intervention Effects for Balance and Postural Control

Nineteen reviews investigated the effectiveness of VR interventions at immediate follow-up for balance and postural control compared with conventional therapy or no intervention. Three meta-analyses included children with CP [36,65,68] and three included people with Acquired Brain Injury (ABI) (e.g., stroke, TBI) [33,57,61]. Thirteen reviews focused on the effect of VR on people with stroke [13,35,37,38,40,42,44,46,47,52,56,62,64]. All included only RCTs except for one that included studies with a pre-post-test design [40]. For CP all reviews reported significant improvements on balance and postural control measures for VR interventions. The magnitude of effects ranged from small to large effects, but with low quality of evidence. For ABI results from three reviews with low and very low quality of evidence did not support better rehabilitation outcomes on measures such as Sit to Stand Test [33] and multiple measures of balance including Berg Balance Scale (BBS) [57,61]. In the case of people with stroke, results reported in the reviews were mixed, depending on the outcome measure used. For BBS [13,38,40,42,47,52,56,62] reported significant improvements for VR, but with effects ranging from low to large in magnitude and quality ranging from low to moderate. Using the same BBS as outcome [35,37,44] identified no effects for VR, but with very low and low quality of evidence. Reviews that used measures such as anteroposterior and mediolateral deviations of the centre of gravity [44] and postural sway measures (e.g., centre of pressure sway/path length) [37,46] did not identify significant improvements for VR with very low and low quality of evidence. Non-significant effects were also reported for the Functional Reach Test (FRT) [42,44,52] and Balance Confidence Scale (BCS) [52,64] with very low and low quality of evidence. A pooled effect based on balance measures such as: BBS, FRT, TUG and Four Step Square Test (FSTQ) significantly favoured VR but was low in magnitude and low in quality [57]. Heterogeneity was low for most comparisons. At up to three months follow-up, only one review [46] reported effects, and in this case they were non significant for VR for people with stroke on balance outcomes, but with very low study quality.

#### 3.2.3. Intervention Effects for Upper Limb, Arm Function and Activity

Eighteen reviews assessed the effectiveness of VR interventions in improving upper limb, arm function and activity for people with stroke, ABI, and CP. Three included children with CP [34,36,48] and reported significant and large effects for VR, but low quality of evidence. Two reviews [36,48] included in their analysis only RCTs, and one [34] reported an analysis based on studies that used a pre-post-test design. One review focused on people with ABI [61] and reported a small but significant effect on the Fugl Myer (FM) Assessment scale with moderate quality of evidence based on RCTs. However, the same study did not identify a significant effect for VR for upper limb function measured using various scales such as the Wolf Motor Test, 9-hole peg test for example, but with low quality of evidence. For people with stroke, five reviews that used FM reported significant improvements for VR [43,50,55,59,62]. The effects were based on RCTs and were moderate to large with very low to high quality. Two reviews with low quality of evidence reported no significant improvements for the VR groups [35,66], noting that both reviews included only RCTs in their analysis. Some reviews that included comparisons between VR and controls on scales such as the Wolf Motor Function Test [60] and Box and Block Test [43,60,62] did not identify any significant improvements for VR interventions, but with very low quality of evidence. To the contrary, one review identified a small but significant effect for upper limb function measured using the Box and Block Test or the Motor Activity Log but with low quality of evidence [55]. Mixed evidence comes from studies which used various upper limb, arm function and activity measures to pool effects. For example, [48,49,51,60] identified significant effects for VR ranging from low to large in magnitude, but with low quality of evidence. Two reviews [49,60] included in their analysis studies that used a pre-post test design. Other reviews that included only RCTs [39,50,53,59,63,66] did not identify any improvements for VR, though the study quality ranged from low to high. In general heterogeneity was low. Only one review [50] reported follow up effects (up to three months) for upper limb function, but the effect was not significant with high quality of evidence.

#### 3.2.4. Intervention Effects for Activity Limitation

Six reviews focused on the effectiveness of VR interventions compared with control interventions for people with stroke and one review on people with ABI. All of them included only RCTs. No review focused on activity limitation of children with CP. For people with ABI Saywell [61] identified a medium and significant effect of VR but with low quality of evidence for independence outcome. For people who had had a stroke two reviews [30,63] identified small and large effects in favour of VR on activities of daily living, but with very low and low quality of evidence. Reported heterogeneity was low. Two reviews [35,43] reported no improvements for VR compared with controls for daily living activities measured using the Barthel Index Scale. Heterogeneity was low for one comparison [35], but substantial in the case of the other [43]. Again, the quality of evidence ranged from very low to low. Two reviews assessed global functioning using the Functional Independence Measure. Domingue-Tellez [43] reported a moderate effect with very low quality of evidence and substantial heterogeneity. Cheok [37] did not identify improvements for VR with low heterogeneity but the quality of evidence was low. Da-Silva [39] reported a significant effect for VR in the case of perceived quality of use of the stroke arm, but no significant results for the perceived amount of use of the stroke arm. For both outcomes, the quality of evidence was rated as very low. None of the reviews included follow up effects for this outcome.

#### 3.2.5. Intervention Effects for ICF WHO Framework: Body Structures/Function, Activity, and Participation

Five reviews investigated the effectiveness of VR for body structures/function, activity, and participation. Two reviews focused on children with CP and three on people with stroke. One review included studies with a pre-post-test design [34] and the rest of the reviews included RCTs. Chen [34] identified significant effects in favour of VR for children with CP for participation and body structure/function. The effects were large and moderate in magnitude, and the quality of evidence was very low and moderate. Noting that the estimates of effects were based on studies which used a pre-post-test design. Chen [36] reported significant improvements in favour of VR for all outcomes for children with CP. Large effects were reported for activity outcome with low quality of evidence. For body function the effect was moderate and the quality was low and for participation the effect was low in magnitude with very low quality of evidence. Results from three reviews suggest significant effects for body structures/functions and activity for people with stroke [10,31,54]. However, the effects were mostly small in magnitude and the quality of evidence ranged from low to moderate. For participation outcome results from two reviews suggested contradictory results. Aminov [31] reported non-significant results with low quality of evidence and [54] reported a moderate effect for VR but with very low quality of evidence for people with stroke. Overall, heterogeneity was mostly low, with a few cases of moderate heterogeneity. None of the reviews included follow up effects for this outcome.

#### 3.2.6. Intervention Effects for Motor Function

Three reviews assessed the effectiveness of VR for motor function. One included children with CP [45] and two included people with stroke [46,50]. Ghai [45] reported a moderate significant effect for gross motor function with low quality of evidence. Noting that the evidence comes from studies with a pre-post design and not RCTs which can lessen the quality of evidence with moderate heterogeneity. For people with stroke, neither of the two reviews which included only RCTs identified significant improvements for the VR groups with quality of evidence ranging from very low to moderate [46,50]. Heterogeneity ranged from low to substantial. There were no reviews that included follow up effects for this outcome.

#### 3.2.7. Intervention Effects for Cognitive Functioning

Only two reviews which included RCTs investigated the effectiveness of VR in improving cognitive functioning for people with stroke [31,67]. Aminov [31] reported a significant small to medium effect size with very low quality of evidence for overall cognition. Heterogeneity was low. While Wiley [67] did not identify any significant results which favour VR on cognitive outcomes such as: global cognition, attention, memory, and language with very low quality of evidence and small to moderate heterogeneity. None of the reviews included follow up effects for this outcome.

### 3.3. Moderator Effects

For our second objective that aimed to identify factors that can enhance rehabilitation outcomes we detected four moderator variables that were reported in reviews (Supplementary Materials, Tables S12–S15).

#### 3.3.1. Mode of Delivery

The first moderator aimed to identify differences in effects between VR standalone interventions and VR interventions delivered in combination with conventional therapy. Three reviews investigated this moderator and all focused on people with stroke [38,41,50]. None of the reviews investigated other conditions. For lower limb outcomes such as gait speed or mobility (measured with TUG) three reviews pointed out no significant differences between the effects of VR interventions delivered alone vs. those combined with conventional therapy [38,41,50]. Similar results emerged for activity limitation [50]. Balance (measured with BBS) results in two reviews were inconclusive as one review [41] indicated positive effects only for VR interventions delivered alone and not for VR combined with conventional therapy. However, another review [50] reported significant effects for VR interventions combined with conventional therapy, but not for VR standalone interventions. A slight benefit reported in one review suggested significant improvements for VR interventions delivered with conventional therapy for upper limb outcomes. Such improvements were not significant for VR interventions delivered alone [50].

In conclusion, the summary of evidence suggests that adding conventional therapy to VR training does not significantly improve lower limb activity, balance and activity limitation outcomes compared to only delivering VR interventions alone. For upper limb function, results suggest better rehabilitation outcomes in the case of VR interventions combined with conventional therapy.

#### 3.3.2. Timed Match Interventions

A second moderator reported in one review compared differences in effects between time dose matched interventions and time non-dose matched interventions for people with ABI [61]. There were no significant effects reported for non-dose matched interventions on any of the outcomes: lower limb gait, upper limb, or FM. Non-significant effects were also identified for dose-matched interventions on lower limb and upper extremity. A small significant effect was reported for FM [61]. For all the comparisons the heterogeneity was low. Based on the above results we might conclude that there is limited evidence to support any differences between interventions that are dose matched and those that are not on physical functioning.

#### 3.3.3. Intervention Length

Two reviews assessed the effect of intervention length (using meta-regression and categorical variables) and reported non-significant effects on upper limb activity for children with CP and people with stroke [34,49]. One review identified that interventions with a total duration greater than 15 h positively impacted upper limb function [55]. Taken together, evidence that supports the significant effect of intervention length on rehabilitation outcomes is mixed.

#### 3.3.4. Technological Features of the VR Platforms

Two moderators focused on identifying if technological features of the VR platforms used produced different effects. Comparisons concerned potential differences between commercially available systems and customized systems [10,31,34,36,50,54] and between VE-based interventions and interactive gaming (IG)-based interventions [47]. Overall, results highlighted the importance of the technological components that underlie VR interventions and stress that specially designed and customized VR interventions were more effective for: upper extremity, ambulation and postural control [36]; arm function [36]; upper limb body function and activity [10]; overall body function and activity [54] with small to large effects and low heterogeneity. VEs -based interventions showed significant improvements with small to moderate effects for functional mobility and balance [47].

### 3.4. Safety Concerns in VR-Adverse Effects

Our third objective aimed to investigate whether VR is safe. Ten out of 41 meta-analysis included in our umbrella review reported adverse effects (see Table 2). Six reviews reported no major adverse effects [35,38,52,56,63,65]. Four reviews reported a few cases of mild adverse effects linked with study participation: transient dizziness and headache, pain, dizziness, increase in hypertonicity, loss of control, increased spasticity, back ache and fatigue [33,37,50,62] (see Table 2).

## 4. Discussion

The current umbrella review assessed if VR based interventions could aid rehabilitation in patients with stroke, ABI and CP. The meta-analyses in this umbrella review identified some beneficial effects of VR-based interventions on physical and cognitive functioning. We included 41 eligible meta-analyses which increased the statistical power. This umbrella review included separate data synthesis for several outcomes of interest: lower limb activity; balance and postural control; upper limb, arm function and activity; activity limitation; ICF WHO Framework (body structures/function, activity, and participation); motor function; cognitive functioning. This allowed us to conduct an in-depth data synthesis to identify for which functional outcome VR works best. Additionally, we quantified the ROB reported in the reviews and assessed the quality of evidence for each outcome to clearly inform researchers and practitioners about the evidence that supports the use of VR interventions. We chose to focus the discussion mostly on evidence that comes from moderate or high quality of evidence [69]. The certainty of the evidence that comes from moderate quality studies suggests that the true effect is probably close to the estimated effect and high quality indicates that the true effect is similar to the estimated effect. To the contrary, evidence of very low and low quality suggests that it is probable that the true effect is different than the estimated effect [69,70]. The data synthesis found mostly low- or very low-quality evidence that supports the effectiveness of VR interventions. Most reviews focused on people with stroke, and only six on children with CP and three on people with ABI. Only a limited number of effects were rated as having moderate and high quality of evidence, but overall, results of moderate and high quality of evidence highlighted potential benefits of VR for improving ambulation function of children with CP, mobility, balance, upper limb function, and body structure/function and activity of people with stroke, and upper limb function of people with ABI. Our results are in line with other studies that investigated the efficacy of VR interventions in various vulnerable populations. For example, significant improvements in VR-based rehabilitation interventions compared with control interventions were also obtained for older healthy adults and older adults with other neurological conditions such as dementia, Parkinson’s Disease, Multiple Sclerosis [71,72,73,74,75].

Mixed evidence of very low quality emerged for cognitive functioning for people with stroke, but no data was available for this outcome in the case of people with ABI, including TBI and children with CP. A lack of reviews that included samples of people with ABI was also identifed in the case of lower limb function, ICF WHO framework (body function, activity, and participation), and motor function. The quality of evidence for most effects was downgraded mainly due to small sample sizes, high ROB of primary studies and failure to include grey literature and conduct a comprehensive literature search (as assessed by four items from AMSTAR) according to the criteria proposed by Pollock [24]. Regarding the ROB, the main weakness was caused by the lack of participants and personnel blinding. We agree with Laver [50] that this domain is more strongly related to the type and intrinsic characteristics of the intervention and less to the study quality. Even if the blinding of participants and personnel might be more difficult for VR-based studies, adding an active control group that can undergo equivalent less immersive VR interventions (e.g., training using interactive gaming or interventions delivered via PCs) may reduce the likelihood of performance bias.

An important question is whether the effects were maintained at follow up. Two reviews identified small effects with small 95% CIs at follow-up (up to three months) for people with stroke on walking speed and gait velocity [38,46]. Effects were not significant for mobility, but the 95% CIs were wide [46]. Because all these effects were rated as having low and very low quality, this restricts our confidence in the estimate of effects. Only one review reported effects at three months follow-up for people with stroke which were not significant with narrow 95% CIs, but with low quality of evidence [46]. In the case of upper limb function one review reported no significant improvements for the VR group, but with high quality of evidence and narrow 95% CIs which reflects enough precision in the effect estimates [50]. Regarding children with CP and people with ABI, including TBI no review assessed VR-based interventions at follow up. Taken together, results suggest that there is currently a lack of reporting of follow up data to assess if the benefits of using VR were sustained in the long run.

Another key point concerns the clinical relevance of the results. Support in favour of VR on the TUG mobility outcome for people with stroke comes from two reviews with moderate study quality of evidence [47,52]. The 95% CIs reported by [47] were small, but those reported by [52] were wide which might limit our confidence in the results. It is important to notice that even if the two reviews pointed out statistical significance for TUG outcomes, the results showed that the effect reflects minimal clinically important changes. In previous studies [76] reported 95% CIs of the smallest real difference (SRD) for TUG between −3.75 to 2.59 s. SRD was proposed as a measure of sensitivity to change. Values that fall outside this range indicate real or clinical changes. Both reviews reported values within these ranges, which limit our ability to conclude that the improvements were real or of practical significance. For people with stroke, two reviews rated as having moderate quality of evidence suggested that VR was more effective than control groups in improving balance as measured with BBS with moderate magnitude of effects and narrow 95% CIs [47,56]. Taking into account the practical significance of these results, [47] calculated coefficients (95% minimal detectable changes) and reported that the effects observed for BBS indicated that the improvements reflect clinically meaningful changes. Such a result strengthens our ability to conclude that the effects reflected real improvements. For upper limb function measured with FM evidence of high-quality pointed out that VR was effective for people with stroke with relatively narrow 95% CI which could indicate that despite some uncertainty there still can be enough precision to highlight the utility of the intervention. However, the mean difference reported by [50] was lower than the minimum value of 7.2 or 9 reported in previous studies for SRD to reflect real or clinical changes [77,78].

In the case of children with CP no data was reported for outcomes measured with individual scales such as TUG mobility, balance measured with BBS, or upper limb function assessed with FM. In these cases outcomes resulted from composite scores from multiple measurement instruments. In terms of magnitude of effects, large effects which suggest meaningful improvements, were obtained for balance and upper limb function, although the quality of evidence remains of very low and low quality. For people with ABI most of the reported effects for balance measured with BBS and Sit to Stand Test were small in magnitude and non significant though with very low and low quality of evidence. For FM outcome results indicated a significant small to moderate effect with moderate quality of evidence.

### 4.1. Factors That Can Enhance Rehabilitation Outcomes

#### 4.1.1. Factors Identified via Moderator Analysis

Our second objective aimed to identify factors that can enhance rehabilitation outcomes and highlight the underlying mechanisms that can explain their effect. Overall, results offer support in favour of customized VR systems compared to commercially available VR systems (e.g., Nintendo Wii, Microsoft Kinect), especially for upper limb extremity, body function and activity. Bespoke VR systems are more likely to follow rehabilitation principles compared to commercial VR by adjusting to user needs and abilities, supporting feedback, task-specific practice and usage of affected limb, and increasing difficulty [10,30,31]. Research using these environments is also more likely to design and conduct usability evaluations with users to select the type of tasks and activities to reach specific rehabilitation goals [73,74]. Even if customized VR systems may require more intensive time for development than off-the-shelf commercial VR systems, they may also be more effective in rehabilitation. Moderators assessing the impact of delivering VR interventions alone or in combination with conventional therapy, and those assessing the length of VR intervention did not have any clinical significance.

#### 4.1.2. Proposed Factors

While performing our literature review and data synthetises, we noticed that the existing literature concerning moderator factors for VR intervention effects was missing some important variables. To cover this gap, informed by a literature review, we propose other factors that might impact VR treatment outcomes such as: type of interaction in VR, components of the VR intervention (e.g., tasks, activities, gaming elements), immersion, presence and participant enjoyment and motivation.

Interaction is achieved mainly via technical capabilities of the VR system (hardware) that allows the user to explore and manipulate the environment, ultimately changing the events [79]. Many primary studies used a form of VR interaction (e.g., motion capture technology to capture patient’s movement) that accommodates neurorehabilitation principles and creates enriched environments to facilitate neuroplasticity by helping patients practice and learn in VR real life tasks and activities. Previous studies showed that interaction in VR improves performance. For example, medical students who manipulated directly and in real-time virtual 3D anatomical structures had better learning outcomes than students who passively viewed the interaction in the same stereoscopic 3D environment [80]. We speculate that environments in which interaction takes place in real time such as the situation in which the VR system responds to the user’s actions and sends feedback can improve rehabilitation outcomes. An example of such real-time interaction is when the participant walks on a treadmill and the speed of the treadmill is adjusted according to user’s movements and the projected VR environment changes the direction while the user moves throughout the environment. Immersion is an objective feature related more to the technology being used to deliver virtual experiences and the ability to simulate the real world and create authentic experiences [3,79]. Some VR systems are more immersive than others. For example, those that use body and head tracking technology coupled with a large field of view displays (e.g., HMDs) to generate a 360° “first person” view of the scenario are highly immersive [3]. Less immersive VR systems use desktop computer screens without motion tracking technology. Presence is a subjective state of consciousness and describes the extent to which people can actually feel they are “there” in the VR [79] and is often measured using questionnaires [81,82]. It is commonly accepted that technological features of the VR systems (e.g., motion tracking technology, field of view and stereoscopy) which make the experience highly immersive increase presence [83]. Adequate immersion and presence help the user to behave in VR as they normally do in real life situations [79] and might contribute to the successful transference of skills and knowledge acquired in VR to the real world [84] though the role of immersion and presence in rehabilitation should further be explored in meta-analyses. Increased interaction in VR was also suggested to be positively related to task enjoyment which can lead to a higher level of programme enjoyment. Research has shown that enjoyment of VR interventions for rehabilitation elevated adherence to therapy [85]. Another mechanism proposed by Howard [71] to explain positive rehabilitation outcomes of VR that is closely related to user needs was participants’ increased excitement which contributes to increased motivation. Adding gaming elements to the application can also boost motivation, engagement and adherence to intervention because people will be less focused on the physical impairment and focus their attention on the experience [75,86]. There is need for further empirical studies to test these proposed factors in order to identify mechanisms that can enhance VR rehabilitation outcomes.

Less emphasis in the stroke, TBI and CP literature was placed on differentiating the methodology used to deliver the intervention than in other domains [87] such as tasks, activities or games. VR tasks refer to specific actions, activities are broader and target high level functions and games follow specific rules [87]. Various tasks, activities and games were used for VR rehabilitation ranging from less complex (e.g., grasping and reaching objects) to more complex (e.g., playing games which require interacting within the game, following rules and keeping score.). In line with rehabilitation principles that stress the importance of task specific practice and gradually increasing task difficulty [9], we suggest designing interventions which start at a low level of complexity with tasks and continue at a higher level with activities and games.

### 4.2. Safety Concerns in VR-Adverse Effects

The few meta-analyses that reported adverse effects did not identify an increased number of adverse effects and none reported severe adverse effects. However, adverse effects in VR should be documented to allow for an informed decision about the safety and feasibility of using VR with vulnerable populations.

### 4.3. Implications for Neurorehabilitation

A main question is whether improvements observed in VR can translate to real life improvements and the underlying clinical impact. Most effects that were expressed via standardized mean differences were of moderate and large magnitude, which suggests that VR-based interventions have clinical significance. Major clinical improvements based on large effects were reported for lower limb activity, balance and postural control. Small improvements were observed for motor function. Some studies computed effect sizes using mean differences for well-established scales such as lower limb activity measured with TUG, balance measured with BBS and upper limb function measured with FM. In the case of these studies we were able to benchmark the results reported from these meta-analyses with SRD values published in other studies that can indicate whether the changes had clinical relevance. For TUG and FM the reported effect sizes were of small magnitude and limited clinical relevance. For BBS the values were large and likely to reflect clinically significant changes.

The most investigated condition was stroke and only a limited number of reviews included children with CP and people with ABI. When it comes to the target population, compelling evidence of moderate and high quality of evidence emerged for people with stroke on most outcomes: mobility, balance, upper limb function, and body structure/function and activity. Evidence of moderate quality in favour of VR for improving upper limb function was reported for people with ABI, including TBI. For children with CP, evidence of moderate quality supports the use of VR interventions for rehabilitation of lower limb activity such as ambulation function.

Larger effects were reported for VR interventions which consisted of various rehabilitation activities (e.g., treadmill walking, gait training for lower limb activity; balance training exercises, postural control exercises for balance and postural control) delivered via commercially available systems and engineer-built systems resulted in greater improvements. VR interventions designated to improve upper limb functions resulted in smaller improvements. Such interventions consisted mostly of VR programs in which people had to perform motor tasks by moving or manipulating virtual objects. Some VR devices were coupled with data gloves to allow for real-time feedback. Several explanations can account for larger effects for lower limb activity and balance versus upper limb function. First, treadmill training and postural VR interventions usually use larger screens or HMDs which allow for increased immersion compared to reaching and grasping tasks that can be delivered on smaller screens which can be less immersive [47]. It was also argued that VR interventions for upper limb rehabilitation should include high intensity training with many repetitions [8,88]. However, two meta-analyses showed that the duration of the intervention does not impact treatment effects for children with CP and people with stroke for upper limb function [34,49]. Based on the synthesis of evidence we could not identify any superiority effects of age e.g., young people outcomes such as those of children with CP compared to older adults such as people with stroke. We also mention that stroke was the most studied condition with increased data availability which could also contribute to the quality and number of trials included in the meta-analysis.

There is a general agreement that VR can provide meaningful and realistic experiences which can facilitate rehabilitation outcomes [8,89]. For example, by being able to repetitively deliver the intervention while gradually increasing the level of difficulty VR can be an efficient means to apply principles of experience-dependent plasticity for rehabilitation of patients with brain damage [9] and principles of motor learning which are known to improve rehabilitation outcomes [8]. Main advantages of VR are accessibility of practice repetition, multisensory feedback, increasing task difficulty, task specificity [8,89]. All the VR interventions included in the meta-analyses included to a degree rehabilitation tasks that allowed for repetition, multisensory and immediate feedback, variability and adaptation of task difficulty to particular user needs. Additionally, evidence from moderation analysis suggests that customizing the VR systems and adapting them to patients’ needs can improve rehabilitation outcomes by implementing rehabilitation principles (e.g., supporting feedback, task-specific practice and usage of affected limb, adjusting for task difficulty).

Despite promising results concerning the effectiveness of VR-based interventions in rehabilitation, there is still inconclusive evidence concerning the successful transference of skills from VR to real life settings [89]. Examples of rehabilitation tasks that follow motor learning principles in VR are reaching movements while wearing an HMD, virtually rotating a hand held virtual object, arm or joint motions to play various sports in VR [89]. In short, the repetitive practice of specific motor skills improves the ability to perform the task. Rehabilitation outcomes are improved if the practice of the motor task takes place in realistic and meaningful environments where multisensory information can modulate performance [90]. It was suggested that successful implementation depends on the software and hardware capabilities [91]. For example, a mismatch in sensory and motor information between the virtual and real environment can lead to a failure of successful skill transfer. Key features of the VR environment such as fidelity (multisensory stimuli: haptic, visual and auditory) and dimensionality lead to successful rendering of the real world tasks to VR, which in turn impacts motor learning and motor execution [89]. Main challenges concern barriers of transfer of learning issues that relate to reduced ecological validity and task specificity. Major limitations can be caused by system delays (e.g., delays in the visual display of stimuli or system latency between participants actions via controllers and the VR system responses) that can reduce the realism of the experience or failure to correctly estimate the perceived distance in virtual environments compared to real situations which can prevent optimal transfer of skills acquired in VR to real life [91]. Addressing technical limitations such as these can improve the ecological validity of the intervention effects.

## 5. Limitations and Future Directions

Our study raises several points of interest for future work. First, we included both RCTs and studies with a pre-test post-test design in order to increase statistical power. However, only four reviews included studies with a pre-test post-test design and 37 reviews included only RCTs. To account for this, we have signposted throughout our review where evidence came for studies with a pre-post-test design which consequently reduced our confidence in the results that came from those reviews.

Our moderation synthetises may be limited by subgroup comparisons performed in reviews. Even though we identified important apriori moderators (e.g., immersion), we were not able to assess directly their contribution to VR effectiveness because the reviews did not account for these variables. In future reviews, it would be useful to identify the effectiveness or superiority of VR interventions by comparing the intervention groups with passive and active control groups. Similarly, identifying whether highly immersive VR environments are more effective than low immersive VR environments will allow for better design of VR protocols for intervention. Even though stroke, TBI and CP have negative impacts on cognitive functions, there is currently a lack of reviews that focus on cognitive rehabilitation. Future reviews should investigate the effect and quality of evidence of VR interventions on cognitive functioning. Currently there is limited data on the cost-effectiveness of VR interventions compared to traditional neurorehabilitation, as none of the reviews provided such data.

## 6. Conclusions

Our umbrella review synthesised a large body of literature on the effects and quality of the evidence of VR-based interventions for physical and cognitive rehabilitation of patients with stroke, TBI and CP. Overall, there is evidence of a benefit of VR in improving physical functioning in people with stroke, TBI and CP, however, most results are based on very low- and low-quality studies. There is a need for high quality RCTs to further investigate the effects of VR interventions.

Our results suggest that the effectiveness of VR interventions is boosted by variables that relate to the technological features of the VR environment, such as customization of VR environments and, possibly, by immersive and interactive VR. We highlight the need to identify and test potential mechanisms that are responsible for effective VR-based rehabilitation, in order to formulate evidence-based guidelines for the design of VR-based rehabilitation interventions.

## Figures and Tables

**Table 1:** Characteristics of the included meta-analytic studies.

Author(s), Year	Population Age	Country of Origin	Sample Size Per Included Meta-Analysis	Number of Primary Studies Include in Meta-Analysis	Clinical Status of the Sample	List of Interventions with VR Component	List of Control Interventions	Type of VR Platform Used for Intervention	VR intervention Time	Outcome/s Measurement Instrument
Ahn et al. [30]	Adults	Korea	694 participants (intervention = 278; control = 416)	9	Stroke ^c^	VR interventions consisted of: motor-based learning; combined and conventional upper limb therapy; various exercises delivered via gaming consoles; reinforced feedback; VR in combination with low frequency repetitive transcranial magnetic stimulation; goal-oriented movement amplification	Control intervention consistent of: standard therapy for upper extremity; conventional occupational therapy; VR in combination with sham repetitive transcranial magnetic stimulation; goal-oriented training in VR without movement amplification	VE-based system (e.g., RehabMaster plus other VR systems that were not specified) IG-based system (e.g., IG type not specified)	687 min (total); 48 min (per session); 12 sessions	Activities of daily living: Performance on daily living activities (Barthel Index scale and Functional Independence Measure)
Aminov et al. [31]	Adults	Australia	955 participants (intervention = 484; control = 471)	31	Stroke (sub-acute, chronic, ischemic, hemorrhagic and mixed)	VR interventions consisted of: various rehabilitation exercises delivered via video capture or tabletop systems with sensors to detect patients’ movements and data gloves to allow for grasping and reaching objects; video capture systems and sensors to allow patients to observe their own movements on the screen and the correct trajectory; reinforced feedback for exercises; various exercises delivered via gaming consoles	Control intervention consisted of: standard therapy; usual activities at the rehabilitation center including social activities, creative crafts and physical activities; physiotherapy alone and in combination with occupational therapy; occupational therapy; computer assisted cognitive rehabilitation; physical therapy with or without occupational therapy	VE-based system (e.g., Reinforced Feedback in Virtual Environment system; Rehab Master game-based system) IG-based system (e.g., Nintendo Wii, Xavix; PlayStation EyeToy; IREX, Xbox Kinect)	685 min (total); 42 min (per session); 18 sessions	ICFWHO framework: Body Structure/Function (e.g., Fugl Meyer Assessment); Activity (e.g., Box and Blocks Test); Participation (e.g., Motor Activity Log) Cognitive functions: Attention (e.g., Continuous Performance Test); Executive functioning (e.g., Trail Making Test- A, Tower of London); Verbal and spatial memory (Visual learning test, Verbal learning test)
Barclay et al. [32] ^a^	Adults	Canada	20 participants (intervention = 11, control = 9)	1	Stroke (chronic)	VR intervention consisted of: motorised treadmill training	Control intervention consisted of: treadmill training without VR	VE-based system (e.g., visual screen with leg sensors and treadmill)	180 min (total); 20 min (per session); 9 sessions	Community ambulation: Time to walk (e.g., Community Walk Test); Walking ability (e.g., Walking Ability Questionnaire; Gait speed (e.g., 10-min walk test); Self-efficacy (Balance Confident Scale)
Booth et al. [33] ^a^	Adults	UK	10 participants (intervention = 9, control = 8)	1	Acquired Brain Injury (e.g., stroke, TBI)	VR intervention consisted of: balance exercises delivered via a game-based platform	Control intervention consisted of: traditional rehabilitation	IG-based system (e.g., Nintendo Wii balance-board)	1200 min (total); 60 min (per session), 20 sessions	Balance: Number of full stands (30 s sit-to-stand test)
Chen et al. [34]	Children	USA	122 participants (intervention = 97, control = 25)	14	Cerebral Palsy (hemiplegia, diplegia, quadriplegia, mixed, spastic)	VR interventions consisted of: various rehabilitation activities delivered via commercially available systems and e engineer-built systems	Control intervention consisted of: conventional therapy	IG-based system (e.g., PlayStation; IREX; NJIT-RAVR; Engineer-built hand system plus EyeToy Play Sony, 5DT 5 Ultra glove plus PlayStation; RE-ACTION system, GX system; NeuroGame, E-link Evaluation and Exercise System; powered joystick linked to computer game, Wii)	539 min (total); 53 min (per session); 13 sessions	ICFWHO framework: Body Structure/Function (e.g., kinematics); Activity (e.g., Bruininks-Oseretsky Test of Motor Proficiency); Participation (e.g., Canadian Occupational Performance Measure)
Chen et al. [35] ^a^	Adults	China	100 participants (intervention = 50, control = 50)	4	Stroke ^c^	VR interventions consisted of: therapy program at home via videoconferencing system; motor task treatment	Control intervention consisted of: VR therapy program in the hospital; traditional physical therapy	VE-based system (e.g., workstation equipped with a 3D motion tracking system which recorded the patient’s arm movements)	1200 min (total); 60 min (per session); 20 sessions	Upper extremity motor function: Fugl Myer assessment Activities of daily living: Performance on daily living activities (Barthel Index scale)
Chen et al. [36]	Children	USA	504 participants (intervention = 266, control = 238)	19	Cerebral Palsy (hemiplegia, diplegia, mixed, spastic)	VR interventions consisted of: physical exercises with interactive games and serious games	Control intervention consisted of: usual care; usual physical activity; modified constraint-induced movement therapy; standard care; regular routine; conventional therapy; no input other than data collection for outcome measures; waiting list control group	IG-based system (e.g., Nintendo Wii; Eloton SimCycle; PlayStation; GestureXtreme; Q4; E-link; Xbox Kinect; engineer-built seat cushion)	1215 (total); 55 (per session); 29 sessions	ICFWHO framework: Body Structure/Function (e.g., Bruininks-Oseretsky Test of Motor Proficiency); Activity (e.g., Pediatric Berg Balance Scale); Participation (e.g., Assessment of Motor and Process Skills) Arm function (e.g., Bruininks-Oseretsky Test of Motor Proficiency); Ambulation function (e.g., 10-min walk test) Postural Control (e.g., Berg Balance Scale)
Cheok et al. [37]	Adults	Singapore	161 participants (intervention = 82, control = 79)	6	Stroke ^c^	VR interventions consisted of: conventional gaming with conventional physical therapy; physical exercises and balance training with interactive games and serious games	Control intervention consisted of: physical activity and or in combination with occupational therapy; general exercises in combination with electrical stimulation to tibialis anterior on affected side; leisure activities, such as playing cards, bingo, or Jenga	IG-based system (e.g., Nintendo Wii)	485 (total); 33 (per session); 14 sessions	Global function (e.g., Functional Independence Measure) Functional mobility (e.g., Timed Up and Go test) and balance (e.g., Berg Balance Scale) Static balance (Postural sway measures: anteroposterior eyes closed; anteroposterior eyes open; medio-lateral eyes closed; medio-lateral eyes open)
Corbetta et al. [38]	Adults	Italy	341 participants (intervention = 169; control = 172)	15	Stroke (ischaemic, haemorrhagic)	VR interventions consisted of: treadmill walking training; stepping over virtual objects on a treadmill; physical exercises for balance and stepping hills; robot training for foot movements in a virtual environment; postural control exercises; balance training exercises with serious games	Control intervention consisted of: conventional rehabilitation with or without treadmill walking training; stepping over real foam objects in a hallway; physical exercises; robot training for foot movements without VR; treadmill training simulating stepping obstacles	VE-based system (e.g., HMDs; audio-visual system combined with a motion-tracking system; projectors, treadmill training system; robotic VR) IG-based system (e.g., Microsoft Xbox 360 Kinect, Nintendo Wii; IREX)	459 (total); 32 (per session); 13 sessions	Walking speed (e.g., the 10-metre walk) Functional mobility (e.g., Timed Up and Go test) Balance (e.g., Berg Balance Scale)
Da-Silva et al. [39] ^a^	Adults	UK	231 participants (intervention = 113; control = 118)	2	Stoke ^c^	VR interventions consisted of: self-directed exercise delivered via interactive gaming system	Control intervention consisted of: tailored arm exercises; no input other than visits to collect outcome measures	IG-based system (e.g., Nintendo Wii; Hand-mounted unit with infra-red light and Nintendo Wiimotes to translate hand movement)	2790 (total); 33 (per session); 107 sessions	Arm function (e.g., Wolf Motor Function Test) Independence and self-care activities: Perceived amount and quality of use of the stroke (e.g., Motor Activity Log)
De Keersmmaecker et al. [40]	Adults	Belgium	105 participants (Pre-test post-test design = 105)	9	Stroke ^c^	VR interventions consisted of: treadmill walking training	n/a	VE-based system (e.g., HMDs; visual screen; treadmill with multiple screens, robotic device Lokomat exoskeleteon with a screen)	646 (total); 35 (per session); 18 sessions	Gait (e.g., Ten-Minute Walk Test) Functional gait (e.g., Berg Balance Scale, Timed Up and Go)
De Rooij et al. [41]	Adults	The Netherlands	516 participants ^b^	21	Stroke ^c^	VR interventions consisted of: treadmill walking training; manipulating objects that are projected on a screen; exercise, balance and/or posture training on interactive gaming systems with or without feedback	Control intervention consisted of: group therapy; conventional therapy with or without task-oriented training; ergometer bicycle training; traditional non-VR treadmill training; physical exercises; conventional therapy plus weight-shift training; conventional therapy plus extra balance therapy; stepping over real foam objects	VE-based system (e.g., HMDs; visual screen; BioRescue platform and monitor) IG-based system (Microsoft Xbox Kinect, PlayStation, Nintendo Wii Fit, SeeMe VR system, IREX)	494 (total); 31 (per session); 15 sessions	Balance Static (e.g., Average postural sway speed) and dynamic balance (e.g., Berg Balance Scale, Timed Up and Go) Gait (e.g., Six-Minute Walk Test)
Domínguez-Téllez et al. [42]	Adults	France, Spain	262 participants (intervention = 131; control = 131)	10	Stroke ^c^	VR interventions consisted of: treadmill walking training; gait training with active gaming component	Control intervention consisted of: traditional non-VR treadmill training; exposure to identical VR, but without balance component training and seated; conventional therapy; task-oriented training; conventional physiotherapy	VE-based system (e.g., HMDs; visual screen) IG-based system (Microsoft Xbox Kinect, Nintendo Wii Balance, IREX)	641 (total); 37 (per session); 15 sessions	Balance (e.g., Berg Balance Scale) Gait (e.g., Ten-Minute Walk Test)
Domínguez-Téllez et al. [43]	Adults	France, Spain	735 participants (intervention = 368; control = 367)	15	Stroke ^c^	VR interventions consisted of: game-based interventions on manipulation of virtual objects, performing various VR tasks, VR with bionic gloves, VR therapy and exoskeleton	Control intervention consisted of: conventional therapy, intensive therapy, functional therapy, recreational therapy	VE-based system (e.g., HMDs; visual screen, CAERN system, mechatronic VR, SmartGlove bionic glove, Armeo Spring exoskeleton, You Grabber bionic gloves) IG-based system (Microsoft Xbox Kinect, PlayStation, IREX, Nintendo Wii)	822 (total); 45 (per session); 19 sessions	Upper limb motor function (e.g., Fugl- Meyer) Quality of life (e.g., Functional independence measure)
Ferreira et al. [13]	Adults	Brazil	310 participants ^b^	11	Stroke (chronic, acute)	VR interventions consisted of: interactive games mainly for balance training with or without feedback	Control intervention consisted of: physical therapy; physiotherapy including balance training; conventional physiotherapy and exposure to identical VR, but without balance component training	IG-based system (e.g., Nintendo Wii, Xbox with Kinect, virtual walking training program using a real-world video recording, IREX; standard computer, an audio-visual output system, and a motion tracking system)	467 (total); 31 (per session); 14 sessions	Functional balance (e.g., Berg Balance Scale) Mobility (e.g., Timed Up and Go)
García-Muñoz et al. [44]	Adults	, Spain	161 participants (intervention = 87; control = 74)	6	Stroke ^c^	VR interventions consisted of: interactive games mainly for balance training	Control intervention consisted of: conventional therapy, occupational therapy, physiotherapy and speech therapy, neurodevelopment training and progressive training	IG-based system (e.g., Nintendo Wii)	527 (total); 33 (per session); 15 sessions	Dynamic balance (e.g., Berg Balance Scale; Timed Up and Go) Static balance (Postural sway measures: anteroposterior deviations of the center of gravity)
Ghai et al. [45]	Children	Germany, India	274 participants (Pre-test post-test design = 274)	14	Cerebral Palsy (spastic diparesis, hemiplegic, mixed)	VR interventions consisted of: gait training with active gaming component; gait training; gait training with interactive gaming; training with anodal transcranial direct current stimulation; ankle movement exercises with customized computer games; treadmill training; ankle training with robot-assistance and VR; home-based virtual cycling training	n/a	IG-based system (Xbox Kinect; Gait Realtime Analysis Interactive Lab; Wii Fit U device; Wii)	1000 (total); 33 (per session); 36 sessions	Gait (e.g., gait velocity, stride length, cadence, stride width) Gross motor function (e.g., Gross motor function test)
Gibbons et al. [46]	Adults	Australia	523 participants (intervention = 262; control = 261)	20	Stroke (acute-subacute, chronic)	VR interventions consisted of: interactive games mainly for balance training; treadmill training; physical exercises; postural control training	Control intervention consisted of: standard care; conventional therapy; exposure to identical VR, but without balance component training and seated; real object training with foam obstacles; task-oriented training; outdoor gait training; ergometer bicycle training; treadmill training; documentary videos	VE-based system (e.g., treadmill plus PC and TV; screen standard computer, synchronized with treadmill velocity; HMDs; visual screen with leg sensors and treadmill) IG-based system (e.g., Nintendo Wii; X-box Kinect; IREX; standard computer, an audio-visual output system, and a motion tracking system; notebook computer, beam projector and screens without tracking system)	541 (total); 30 (per session); 18 sessions	Functional balance (e.g., Berg Balance Scale) Static balance (Postural sway measures: COP velocity, COP sway/path-length eyes open and eyes closed, percentage WB on affected limb) Functional mobility (e.g., Timed Up and Go) Spatiotemporal characteristics/kinematics of gait (e.g., 10 m walk test, cadence, stride length, step length, stance time) Motor function (e.g., Fugl- Meyer) Muscle tone (Tardieu scale) Balance confidence and falls (e.g., ABC questionnaire)Walking ability (e.g., Walking ability questionnaire)
Iruthayarajah et al. [47]	Adults	UK	413 participants ^b^	17	Stroke (chronic ischaemic, haemorrhagic)	VR interventions consisted of: interactive games and VR mainly for balance training; treadmill training; physical exercises; postural control training; exercise program with cognitive tasks; VR training at home; treadmill gait training in VR in combination with functional electric stimulation	Control intervention consisted of: task-oriented training; general exercise therapy; conventional weight-shift training; conventional physical therapy; no intervention, resumed with normal routine; occupational and physical therapy; balance training; treadmill training with or without physical therapy; treadmill gait training; conventional physiotherapy; environmental documentary; proprioceptive neuromuscular facilitation exercise program; VR training in clinic; treadmill gait training in combination with functional electric stimulation	VE-based system (e.g., real-world video recordings connected to a treadmill and PC and projector without tracking system; HMD and treadmill; HMD, treadmill with optic flow; IREX HMD; standard computer, an audio-visual output system, and a motion tracking system, video display and an audio system; notebook computer, beam projector and screens; real-world video recordings connected to a treadmill and stabilizing system) IG-based system (e.g., Nintendo Wii; Xbox Kinetic; BioRescue)	461 (total); 31 (per session); 23 sessions.	Dynamic balance (e.g., Berg Balance Scale, Timed Up and Go)
Johansen et al. [48]	Children	Norway	222 participants (intervention = 113, control 109)	7	Cerebral Palsy (hemiplegia, diplegia, tetraplegia)	VR interventions consisted of: motion controlled video games for hand and arm function	Control intervention consisted of: no treatment, conventional therapy	IG-based system (e.g., Nintendo Wii; Wii sports; Wii fit; Xbox 360; Kinect Sports Adventures, PlayStation EyeToy, Wii sports resort	755 (total); 38 (per session); 20 sessions	Hand and arm function (e.g., Jebsen-Taylor Hand Function Test, Quality of Upper Extremity Skills Test)
Karamians et al. [49]	Adults	US	1198 participants (intervention) ^b^	38	Stroke (acute, chronic, mixed)	VR interventions consisted of: VR and game-based interventions for upper extremity rehabilitation	Control intervention consisted of: conventional/traditional occupational therapy, task-oriented rehabilitation, task-related practice, Bobath and neurodevelopmental treatment, stretching, strengthening and activities of daily living	VE-based system IG-based system	975 (total)	Upper extremity function (e.g., Wolf Motor Functioning Test, Fugl Meyer, Action Research Arm Test)
Laver et al. [50]	Adults	Australia	1827 participants (intervention = 827, control 1000)	50	Stroke (ischaemic, hemiparesis, haemorrhagic)	VR interventions consisted of: interactive games and VR mainly for balance training; treadmill training; physical exercises with or without feedback; postural control training; exercise program with cognitive tasks; VR training at home; treadmill gait training in VR in combination with functional electric stimulation	Control intervention consisted of: usual care; therapy provided based on the Bobath approach; Wii gaming system; training using the PSS CogRehab program; computer-assisted rehabilitation; leisure activities including cards, bingo and Jenga; ergometer bicycle training with feedback; pointing at targets in a physical environment; VR intervention without feedback; general exercise/physical therapy; no intervention; occupational therapy; balance training; treadmill training with or without physical therapy; conventional physiotherapy	VE-based system (e.g., HMDs and data gloves) IG-based system (e.g., Nintendo Wii; customized games running on laptop; depth sensing camera and display on a television screen; videogames with a robotic arm; IREX; Armeo^®^Spring arm orthosis interactive games, rehabilitation gaming system; Kinect; Sony Play Station; SeeMe VR; arm orthosis (T-WREX) combined with custom-designed software package, video capture system; Space Balance 3D; VFT system which consists of a computer, a monitor, and a force plate; Rutgers ankle rehabilitation system connected to a desktop computer; VR therapy which consists of an exoskeleton connected to the computer; RehabMaster™; CAREN system, actuated virtual keyboard)	760 (total); 45 (per session); 17 sessions	Arm function and activity (e.g., Fugl Meyer) Hand function (e.g., grip strength) Gait and balance: Lower limb activity (e.g., Timed Up and Go Test), Balance and functional control (e.g., Berg Balance Scale) Global motor function (e.g., Motor Assessment Scale)Activity limitation: activities of daily living (e.g., Functional IndependenceMeasure)
Lee et al. [51]	Adults	Korea	542 participants (intervention = 276, control = 198)	21	Stroke (chronic)	VR interventions consisted of: balance, muscular, strength exercises; arm and finger rehabilitation exercises; reinforced feedback; exercises for upper limb function; treadmill training; various commercial and/or serious games delivered via gaming consoles; VR with robotic gait training; exercises delivered via serious games in combination with low frequency repetitive transcranial magnetic stimulation; exercises delivered via serious games plus mental practice	Control intervention consisted of: treadmill training without VR; robotic gait training without VR; physical and occupational therapy; physical exercises; passive control group; ergometer training; serious games exercises in combination with sham repetitive transcranial magnetic stimulation; exercises delivered via serious games without mental practice	VE-based system (e.g., treadmill with real-world video recording; other VEs not fully described) IG-based system (e.g., Wii, Nintendo; Xbox Kinect)	897 (total); 47 (per session); 21 sessions	Upper limb function (e.g., Jebsen Taylor Hand Function Test) Lower limb function (e.g., speed and cadence)
Li et al. [52]	Adults	China	428 participants (intervention = 230, control = 198)	16	Stroke (acute-subacute, chronic)	VR interventions consisted of: balance training; treadmill training; physical therapy; treadmill training in VR in combination with functional electric stimulation	Control intervention consisted of: physical and occupational therapy with or without functional electrical stimulation; conventional weight-shift training; balance training; treadmill gait training in combination with functional electric stimulation; walking training	VE-based system (e.g., HMD and treadmill with and without suspension; treadmill, PC, projector and screen with optic flow; treadmill, PC, screen with sensors; standard computer, an audio-visual output system, and a motion tracking system with video display and an audio system) IG-based system (e.g., Nintendo Wii, IREX; TV screen and balance board Wii; treadmill walking training with real-world video recording and a camera stabilizing system)	386 (total); 26 (per session); 16 sessions	Balance (e.g., Berg Balance Scale, Timed Up and Go Test) Activities specific (ABC) Forced platform indicators (e.g., sway velocity, weight distribution)
Lin et al. [53] ^a^	Adults	Taiwan	684 participants (intervention = 321, control = 360)	9	Stroke ^c^	VR interventions consisted of: balance training; physical training; arm support training	Control intervention consisted of: recreational activities; no additional treatment; interactive gaming balance-based rehabilitation in seated position; conventional reach training; physical exercises	VE-based system (e.g., ArmeoBoom integrated with a webcam and a laptop and an adjusted 3D virtual environment) IG-based system (e.g., Nintendo Wii, IREX; hand-held remote controller with a base movement sensor, PC, and display screen)	339 (total); 43 (per session); 15 sessions	Upper extremity (e.g., Fugl Meyer) Lower extremity (e.g., Timed Up and Go Test)
Lohse et al. [54]	Adults	USA	626 participants (intervention = 321, control = 305)	24	Stroke ^c^	VR interventions consisted of: arm exercises; treadmill training; balance training; exercise therapy; reflection therapy; street crossing cognitive training; treadmill gait training in VR in combination with functional electric stimulation; functional tasks; skills training; reinforced feedback therapy; upper limb training at home	Control intervention consisted of: recreational activities; treadmill training, balance training; occupational therapy with or without physiotherapy; computer-based visual scanning tasks; traditional therapy, physiotherapy; treadmill gait training in combination with functional electric stimulation; psychoeducational training; no intervention	VE-based system (e.g., treadmill, projector and laptop not synchronized; HMD with a motion tracking system; desktop computer; PC, LCD projector, tracking system and data gloves; Rutgers ankle rehabilitation system and a six-degree of freedom Stewart platform force-feedback system; PC workstation, a high-resolution LCD projector, a 3D motion-capture system; PC, video-camera and 3D motion-capture system; CAERN system) IG-based system (e.g., semi-immersive workbench with haptic device and stereoscopic glasses; Nintendo Wii, IREX; computer and data gloves synchronized; computer and camera)	698 (total); 41 (per session); 17 sessions	ICFWHO framework: Body Structure/Function (e.g., Fugl Meyer Assessment); Activity (e.g., Berg Balance Scale); Participation (e.g., Jebsen-Taylor Hand Function Test)
Maier et al. [10]	Adults	Spain	1470 (intervention = 817, control = 653)	30	Stroke (chronic, subacute, acute)	VR interventions consisted of: various task practices with or without feedback	Control intervention consisted of: conventional therapy; occupational therapy; physical therapy; passive control	VE-based system (e.g., TV and motion tracking; HMD and motion tracking; computer and motion tracking through computer vision and data gloves) IG-based system (e.g., Wii; PlayStation EyeToy; Kinect)	1374 (total); 51 (per session); 17 sessions	ICFWHO framework, only upper limb outcomes: Upper limb body function (e.g., Brunnstrom Motor Recovery Stage) Upper limb activity (e.g., Action Research Arm Test)
Mekbib et al. [55]	Adults	China, USA	1292 (intervention = 650, control = 642)	27	Stroke (chronic, subacute)	VR interventions consisted of: upper limb exercises using customized or interactive gaming platforms with or without feedback	Control intervention consisted of: conventional therapy, physical therapy, occupational therapy for upper limb	VE-based system (e.g., adaptive reality rehabilitation system that uses a camera to track patients’ movements, VR system with feedback) IG-based system (X-box Kinect, YouGrabber, Rehabilitation Gaming System (RGS), NintendoWii, FurballHunt tabletop rehabilitation game, RehabMaster game, Smart Glove, PneuGlove, MusicGlove	797 (total); 44 (per session); 18 sessions	Upper limb function (e.g., Fugl Myer, Box and Block Test, Motor Activity Log)
Mohammadi et al. [56]	Adults	Iran, USA	325 (intervention = 166, control = 159)	13	Stroke (chronic, subacute)	VR interventions consisted of: exercises; treadmill training; game-based physical exercises	Control intervention consisted of: motion, stretching, strengthening, therapeutic exercises; functional electrical stimulation; gait and balance training; neurodevelopmental training; functional activities; treadmill training without VR	VE-based system (e.g., monitor, speakers, and a static or dynamic balance training surface or floor space) IG-based system (Wii Fit; IREX system, Microsoft X-box Kinect, BalPro, BalTrak, BioRescue systems)	465 (total); 30 (per session); 15 sessions	Balance (Berg Balance Scale)
Prosperini et al. [57]	Adults	Italy	521 (intervention = 266, control = 255)	20	ABI (stroke, TBI)	VR interventions consisted of: various balance exercises delivered via commercially available gaming consoles	Control interventions consisted of: standard physical therapy, cognitive rehabilitation, waiting list, balance platform therapy, balance re-training, mirror visual feedback training, stationary cycling, vidogames	IG-based system (Wii balance board, Dance Dance Revolution, Kinect)	651 (total); 37 (per session); 20 sessions	Balance (Berg Balance Scale, Timed Up and Go Test)
Rodrigues-Baroni et al. [58]	Adults	Brazil	154 participants^b^	7	Stroke (chronic)	VR interventions consisted of: treadmill training; game-based physical exercises; exercises with feedback	Control intervention consisted of: treadmill training; no intervention; exercises without feedback	VE-based system (e.g., treadmill projector and laptop not synchronized; treadmill and HMD; treadmill training or stretching exercises; Rutgers ankle rehabilitation system and a six-degree of freedom Stewart platform force-feedback system; treadmill, Pc and projector) IG-based system (e.g., Wii; PlayStation EyeToy; Kinect; IREX system)	549 (total); 41 (per session); 13 sessions	Walking speed (e.g., timed walk measure based upon the 10-Meter Walk Test)
Rutkowski et al. [59]	Adults	Poland, Italy	271 (intervention = 144, control = 127)	10	Stroke (chronic, subacute)	VR interventions consisted of: upper limb exercise, grasp and reach exercise, cognitive exercises, interactive video games, practice specific grips to play music notes or songs	Control intervention consisted of: conventional physicaltherapy, exercisesfor increased trunk stability, lower extremity musclestrength, gait ability, occupational therapy, motion and strengthening exercises, table-top activities, training for functional outcomes	VE-based system (e.g., treadmill projector and laptop) IG-based system (e.g., Rehabilitation Gaming System, Virtual realityreflectiontherapy, IREX system, RehabMaster, RAPAEL Smart Glove, ctuated virtualkeypad system(AVK) withPneuGlove, MusicGlove)	577 (total); 24 (per session); 24 sessions	Upper limb function (e.g., Fugl Myer) Lower limb function (gait) (e.g., Timed Up and Go Test)
Saposnik et al. [60]	Adults	Canada	195 participants (intervention = 54, control = 49, pre-test post-test design = 92)	12	Stroke (chronic, subacute)	VR interventions consisted of: exercise therapy; motor exercises	Control intervention consisted of: exercise therapy; motor exercises	VE-based system (e.g., PC and electromagnetic motion tracking system; PC and data gloves; HMDs, VR curved screen; PC, a high-resolution LCD projector, a wall screen, a 3D motion-tracking system and simple manipulable objects; pneumatic hand orthosis and HMD; pneumatic glove and HMD) IG-based system (e.g., IREX; Nintendo Wii; PlayStation EyeToy; VR Motion; semi-immersive workbench, handheld stylus and stereoscopic shuttered glasses)	1562 (total); 65 (per session); 17 sessions	Motor function (e.g., Wolf motor function test) Motor impairment (e.g., Fugl-Meyer)
Saywell et al. [61]	Adults	New Zealand	612 participants (intervention = 304, control = 296, pre-test post-test design = 12)	23	Acquired Brain Injury (stroke, TBI)	VR interventions consisted of: physical exercise delivered via serious games; hand exercises; balance training; physical exercise delivered via serious games with electrical stimulation; treadmill training with functional electrical stimulation; robotic therapy performing the ankle movements using the robotic system and VR	Control intervention consisted of: traditional stroke rehabilitation; no intervention; physiotherapy with or without occupational therapy; physical exercise delivered via serious games without VR component; traditional hand exercises; traditional reach-training; no intervention; general exercise and electrical stimulation; robotic therapy performing the same ankle movements using the robotic system without VR	VE-based system (e.g., PC, LCD projector, tracking system and data gloves; MusicGlove and IsoTrainer) IG-based system (e.g., semi-immersive workbench, handheld stylus and stereoscopic shuttered glasses; Nintendo Wii; IREX; X-Box Kinect; HMD, motion tracking system and sensors; PlayStation EyeToy, T-WREX weight-supported arm orthosis; ArmeoBoom device integrated with a webcam and a laptop; eBaViR; Rutgers ankle rehabilitation system connected to a desktop computer)	722 (total); 41 (per session); 18 sessions	Upper limb (e.g., Wolf motor function test) Lower limb balance measures (e.g., Berg Balance Scale) Lower limb gait measures (e.g., Timed Up and Go Test) Measures of independence (e.g., Functional independence measure) Fugl Meyer
Tay et al. [62]	Adults	Malaysia	436 participants ^b^	17	Stroke (chronic, acute)	VR interventions consisted of: balance training in standing position; upper limb exercises in standing position with feedback; upper limb exercises with feedback; balance training using serious games; arm support training; gesture therapy	Control intervention consisted of: balance training in a seated position; occupational therapy and/or Wii upper limb exercises without feedback; physiotherapy and occupational therapy; occupational therapy; proprioceptiveneuromuscular facilitation exercise program; occupational therapy and exercises	VE-based system (e.g., hand-held remote controller detected with a base movement sensor, laptop computer and a 32-inch liquid crystal-display screen; ArmeoBoom integrated with a webcam and a laptop and an adjusted 3D virtual environment) IG-based system (e.g., Nintendo Wii, IREX, RGS, Tetrax Biofeedback System center of pressure controlled; FurballHunt game which consisted of a horizontally placed screen with webcam and motion capture software; BioRescue platform with pressure sensors and monitor; Gesture Xtreme video-capture system, Rehab Master, You Grabber with PC, screen and gloves containing sensors; Gesture Therapy; PC, tracking system and data gloves; MoU-Rehab- a combination of two mobile devices: a tablet PC and a smartphone with a Bluetooth connection; RehabMaster with PC, monitor, and depth sensor; T-WREX weight-supported arm orthosis with PC, a web cam, and a hand grip)	642 (total); 37 (per session); 16 sessions	Balance and gait (e.g., Berg Balance Scale, Timed Up and Go Test, walking tests) Upper limb function (e.g., Fugl-Meyer, Box and Block Test)
Veerbeek et al. [63]	Adults	The Netherlands	507 participants ^b^	23	Stroke ^c^	VR interventions consisted of: finger movement and tracking exercise at home and in the clinic in an fMRI scanner; reinforced feedback; arm therapy; exercise training using serious games; arm exercises at home; therapy program at home via videoconferencing system; gesture therapy; hand opening exercises with pneumatic hand support and feedback; hand exercises; stepping over virtual objects on a treadmill; training on how to access and use the station facilities of the Mass Transit Railway; treadmill training; balance and exercise training; foot exercises with visual and auditory feedback; robotic therapy performing the ankle movements using the robotic system and VR	Control intervention consisted of: no intervention/waiting list; conventional rehabilitation; conventional exercises; VR arm exercises in the clinic; physiotherapy and occupational therapy; exercises for the upper limb; therapy provided based on the Bobath approach; occupational therapy; recreational activities; movement training; grasp-and-release of the virtual and actual objects exercises but without any assistance of hand opening; conventional therapy; stepping over foam objects in a hallway; psycho-educational programme with video modelling; treadmill training; static and dynamic balance training; foot exercises without visual and auditory feedback; robotic therapy performing the same ankle movements using the robotic system without VR	VE-based system (e.g., PC, projector, and potentiometer; PC workstation, a high-resolution LCD projector, a 3D motion-capture system; HMD, data gloves, tracking system; workstation equipped with a 3D motion tracking system; computer workstation connected to a 3D motion-tracking system and projector; ArmeoBoom integrated with a webcam and a laptop and an adjusted 3D virtual environment; HandTutor gloves; pneumatic hand orthosis and HMD; HMD and treadmill; PC with desktop; treadmill, PC, visual screen with electromagnetic tracking system) IG-based system (IREX; Wii; Semi-immersive workbench with haptic device and stereoscopic glasses; Playstation EyeToy; PC, tracking system and data gloves; Rutgers ankle rehabilitation system connected to a desktop computer)	Range between 3 to 7 days per week; 10 days to 7 weeks; 20 to 60 min per session	Gait speed and maximum gait speed (e.g., 10-m walk test) Step length (gait analysis)Walking ability (e.g., Walking ability questionnaire) Motor function arm (e.g., Fugl-Meyer) Muscle tone (e.g., Modified Ashworth scale) Arm-hand activities (unilateral) (e.g., Box and Block Test) Arm-hand activities (bilateral) (e.g., Chedoke arm and hand activity inventory) Basic ADL (e.g., Functional independence measure)
Wang et al. [64] ^a^	Adults	China	41 participants (intervention = 22, control = 19)	2	Stroke ^c^	VR interventions consisted of: treadmill training	Control intervention consisted of: traditional treadmill training; treadmill training, without control over the slope of the treadmill	VE-based system (e.g., treadmill, PC, visual screen with electromagnetic tracking system; treadmill and HMD)	12 sessions ^d^	Balance (ABC scale)
Warnier et al. [65]	Children	The Netherlands	194 participants ^b^	7	Cerebral Palsy (hemiplegic, diplegic, spastic quadriplegia, spastic diplegic, athetoid, ataxic)	VR interventions consisted of: interventions for balance and walking using gaming component, intervention using virtual cycling	Control intervention consisted of: other interventions than VR	VE-based system (e.g., virtual cycling system with interactive workout) IG-based system (customized PC gaming, TYMO-Tyromotion, Wii Games, Nintendo Wii Fit balance board and balance based video game, jogging program)	1144 (total); 31 (per session); 39 sessions	Balance (e.g., Timed Up and Go Test, Functional reach test) Walking (e.g., 10-m walk test)
Wattchow et al. [66] ^a^	Adults	Australia	76 participants ^b^	4	Stroke ^c^	VR interventions consisted of: motor tasks; conventional gaming tasks; customized rehabilitation gaming tasks	Control intervention consisted of: conventional therapy plus motor tasks without VR; physical and occupational therapy	VE-based system (e.g., hand-held remote controller detected with a base movement sensor, laptop computer customized rehabilitation gaming soft- ware and a 32-inch liquid- crystal-display screen) IG-based system (e.g., IREX)	576 (total); 25 (per session); 25 sessions	UL impairment (e.g., Fugl-Meyer) UL activity (e.g., Barthel Index)
Wiley et al. [67]	Adults	Canada	122 participants (intervention = 63, control = 51)	5	Stroke	VR interventions consisted of: cognitive training and rehabilitation, activities of daily livingsimulation, reaching tasks combined with cognitive training,	Control intervention consisted of: computerized cognitive rehabilitation, traditional cognitive rehabilitation, spatial and timeorientation techniques, writingtraining, occupational therapy and physiotherapy	VE-based system (e.g., Joystim system) IG-based system (e.g., IREX, Reh@City, Reh@Task)	600 (total); 40 (per session); 15 sessions	Cognitive function: global cognition (MMSE), attention (Trail Making Test A), Memory (Digit Span Test), language (Stroke Impact ScaleCommunication Domain and Neurobehavioral Functioning Inventory-Communication Domain)
Wu et al. [68]	Children	China, US	448 participants (intervention = 226, control = 222)	11	Cerebral Palsy	VR interventions consisted of: game-based interventions for balance and walking	Control intervention consisted of: regular rehabilitation, strength training, neurodevelopmental therapy	IG-based system (Nintendo Wii Fit, Nintendo Wii Fit balance board, Xbox Kinect, Q4 situational interactive rehabilitation training system)	1193 (total); 31 (per session); 40 sessions	Balance and gait (e.g., Berg Balance Scale, Timed Up and Go Test)

Note. ^a^ = data extracted from subgroup analysis performed in the meta-analysis; ^b^ = number of participants in the intervention and control group could not be extracted; ^c^ = condition sub-types not specified; ^d^ = duration of session is not reported; n/a = not applicable, pre-test post-test design.

**Table 2:** Adverse effects. Summary of findings.

Author(s), Year	Number of Primary Studies Which Reported Adverse Events	Reported Results and Severity of Symptoms
Corbetta et al. [38]	1 study	No major adverse effects.
Laver et al. [50]	23 studies	19 studies reported no significant adverse events linked to study participation; 4 studies reported: transient dizziness and headache (2 cases); pain (2 cases); pain and dizziness (several participants) not related to intervention; increase in hypertonicity (3 cases).
Booth et al. [33]	3 studies	Minor adverse effects: either no effects noted, loss of control, or dizziness.
Chen et al. [35]	No study reported adverse effects	N/A
Cheok et al. [37]	1 study	Minor adverse effects: increased spasticity (3 cases).
Li et al. [52]	1 study	No major adverse effects.
Tay et al. [62]	4 studies	Mild pain, back ache and fatigue (4 studies).
Mohammadi et al. [56]	2 studies	No major adverse effects.
Veerbeek et al. [63]	23 studies	No major adverse effects.
Warnier et al. [65]	7 studies	No major adverse effects.

## Supplementary Materials

The following are available online at https://www.mdpi.com/article/10.3390/jcm10071478/s1, Table S1: Excluded meta-analysis with reasons, Table S2: AMSTAR 2 quality assessment of meta-analyses of randomized and non-randomized studies, Table S3: Risk of bias for the included studies, Table S4: Details of included reviews. Reported effects of interventions and quality of evidence for reported outcomes: Lower limb activity, Table S5: Details of included reviews. Reported effects of interventions and quality of evidence for reported outcomes: Balance and postural control, Table S6: Details of included reviews. Reported effects of interventions and quality of evidence for reported outcomes: Upper limb, arm function and activity, Table S7: Details of included reviews. Reported effects of interventions and quality of evidence for reported outcomes: Activity limitation, Table S8: Details of included reviews. Reported effects of interventions and quality of evidence for reported outcomes: ICF WHO Framework: body structures/function, activity and participation, Table S9: Details of included reviews. Reported effects of interventions and quality of evidence for reported outcomes: Motor function, Table S10: Details of included reviews. Reported effects of interventions and quality of evidence for reported outcomes: Cognitive functioning, Table S11: Reported effects for follow-up, Table S12: Subgroup comparisons. Moderator effects for comparisons between VR interventions alone versus VR interventions with conventional therapy, Table S13: Subgroup comparisons. Moderator effects for comparisons between time dose matched interventions versus time non-dose matched interventions, Table S14: Subgroup comparisons. Moderator effects for comparisons between commercially available systems versus customized systems, Table S15: Subgroup comparisons. Moderator effects for comparisons between VE-based interventions versus IG-based interventions
