# Supplementary material for: Virtual Reality in Neurorehabilitation: An Umbrella Review of Meta-Analyses
Source: J Clin Med. 2021 Apr 2;10(7):1478. doi: 10.3390/jcm10071478 (PMC8038192; doi:10.3390/jcm10071478)
Supplement: Supplementary file 1 [file jcm-10-01478-s001.zip › Supplem files/Appendix 1-Supplemental_Data.docx]

Complete search string for Scopus

( TITLE-ABS-KEY ( "Virtual reality"  OR  "vr"  OR  "virtual environment"  OR  game  OR  immersive )  AND  TITLE-ABS-KEY ( rehab*  OR  improv*  OR  train*  OR  intervention  OR  treat*  OR  expos*  OR  remediat* )  AND  TITLE-ABS-KEY ( meta-analy*  OR  review ) )  AND  ( LIMIT-TO ( LANGUAGE ,  "English" ) )

Search date: February 11^th^, 2020

Hits: 7359

( TITLE-ABS-KEY ( "virtual reality" OR "vr" OR "virtual environment" OR game OR immersive ) AND TITLE-ABS-KEY ( rehab* OR improv* OR train* OR intervention OR treat* OR expos* OR remediat* ) AND TITLE-ABS-KEY ( meta-analy* OR review ) ) AND PUBYEAR > 2018 AND ( LIMIT-TO ( LANGUAGE , "English" ) )

Search date: December 24^th^, 2020

Hits: 1044

Supplementary Table S1

*Excluded meta-analysis with reasons*

| Author(s), year | Reasons for exclusion |
| --- | --- |
| Agostini et al., 2015 | Not population of interest; heterogeneous population: neurological, total knee arthroplasty (TKA), cardiac; two studies that focused on VR interventions and no subgroup analysis on VR was reported |
| Alvarez-Bueno et al., 2017 | Not population of interest: healthy children and adolescents |
| Alwardat et al., 2018 | Not population of interest: Parkinson Disease |
| Araujo et al., 2019 | Not intervention of interest: balance-training interventions, but no subgroup analysis for VR was reported |
| Balasukumaran et al., 2018 | Not population of interest; heterogeneous population: people with gait impairments (e.g., osteoarthritis of the knee, low back pain) knee pathology, stroke, Parkinson Disease, Cerebral palsy, juvenile rheumatoid arthritis, low back pain |
| Bania et al., 2019 | Not intervention of interest: activity training on the ground, but no subgroup analysis for VR was reported |
| Bediou et al., 2018 | Not population of interest: children, younger adults, older adults |
| Booth et al., 2018 | Not intervention of interest: gait training enhanced with VR combined with biofeedback |
| Buchignani et al., 2019 | Not intervention of interest: action observation training |
| Burton et al., 2019 | Not population of interest: older people in the community |
| Busk et al., 2019 | Not population of interest: older people in the community |
| Cabrales, 2018 | Mixed methodology: “Study data points were matched up via visual  approximation to create equivalent pairings between virtual reality and mirror therapy studies” (Cabrales, 2018, p. 11) |
| Cacciata et al., 2019 | Not population of interest: older people adults |
| Cao et al., 2018 | Not intervention of interest: exercise intervention without VR component |
| Carvalho et al., 2017 | Not intervention of interest: robotic gait training without VR component |
| Chen et al., 2014 | Not intervention of interest: constraint-induced movement therapy without VR component |
| Choi & Hector, 2012 | Not population of interest: older adults |
| Chung et al., 2016 | Not intervention of interest: resistance training without VR component |
| Cooke et al., 2010 | Not intervention of interest: exercise-based therapies without VR component |
| Corbetta et al., 2010 | Not intervention of interest: constraint induced movement therapy without VR component |
| Corregidor-Sánchez et al., 2020 | Not population of interest: older adults without neurological conditions |
| Cottrell et al., 2017 | Not intervention of interest: real-time telerehabilitation without VR component |
| Coupar, 2012 | Not intervention of interest: simultaneous bilateral training |
| Coussement, 2008 | Not population of interest; heterogeneous population: acute- and chronic-care hospitalized |
| Crocker et al., 2013 | Not population of interest: older residents of long-term care and no mention of any cognitive/neurological impairment |
| Cruickshank et al., 2015 | Not population of interest: Parkinson Disease and Multiple Sclerosis |
| Da Rocha et al., 2015a | Not population of interest: Parkinson Disease |
| Da Rocha et al., 2015b | Not population of interest: Parkinson Disease |
| Dillon et al., 2018 | Not population of interest: adults aged above 50 years with visual impairment |
| Dockx et al., 2016 | Not population of interest: Parkinson Disease |
| Elbanna et al., 2019 | Not intervention of interest: noninvasive brain stimulation, but no subgroup analysis for VR was reported |
| Elsner et al., 2016 | Not intervention of interest: compared active transcranial direct-current stimulation (tDCS) with an active control intervention (physiotherapy or virtual reality) |
| Falck et al., 2019 | Not population of interest: older adults |
| Fedewa et al., 2016 | Not population of interest: overweight and obese participants |
| Finnegan et al., 2019 | Not population of interest: older adults |
| French et al., 2010 | Not intervention of interest: repetitive task training |
| Goodwin et al., 2008 | Not population of interest: Parkinson Disease |
| Grande et al., 2019 | Not intervention of interest: interventions promoting physical activity |
| Hamilton et al., 2018 | Not intervention of interest: transcranial direct-current stimulation (tDCS) combined with VR compared with an active control intervention (VR) |
| Hanratty et al., 2012 | Not population of interest: Subacromial impingement syndrome |
| Harris et al., 2015 | Not population of interest: Parkinson Disease and older adults |
| Hart & Buck, 2019 | Not population of interest: older adults; Not intervention of interest: resistance training |
| Hayward et al., 2014 | Not intervention of interest: altering a single component of a rehabilitation programme (e.g. adding bilateral practice alone) on functional recovery after stroke |
| Hillier & McDonnell, 2016 | Not population of interest: adults diagnosed with symptomatic unilateral peripheral vestibular dysfunction living in the community |
| Hopewell et al., 2019 | Not intervention of interest: multifactorial interventions for preventing falls in older people |
| Howard, 2017 | Not population of interest; population too heterogeneous and type of conditions were not fully described and set as an inclusion/exclusion criteria |
| Hugues et al., 2019 | Not intervention of interest: physical therapy |
| Kalron & Zeilig, 2015 | Not population of interest: exercise intervention programs (aerobic, yoga, sport climbing, VR); one study focused on VR interventions but no subgroup analysis on VR was reported |
| Kayambu et al., 2013 | Not population of interest: critically ill |
| Isabel Corregidor-Sanchez et al., 2020 | Not population of interest: older adults |
| Lal et al., 2018 | Not population of interest: patients with concussion |
| Lau et al., 2017 | Not population of interest: patients with Asperger, ADHD, Depression, healthy elderly |
| Logan et al., 2003 | Not intervention of interest: occupational therapy and leisure therapy |
| Lopéz et al., 2020 | Other intervention: computerized training without VR component |
| Louie et al., 2018 | Not intervention of interest: extremity mirror therapy without VR component |
| Lucas et al., 2016 | Not population of interest: children with mild to moderate gross motor disorders; Not intervention of interest: aquatic therapy, Taekwondo, Treadmill training without body weight, balance training |
| Lundell et al., 2015 | Not population of interest: chronic obstructive pulmonary disease |
| Mansfield et al., 2015 | Not population of interest: older adults and Parkinson Disease |
| Mansor et al., 2019 | Not population of interest: older adults |
| Martins et al., 2016 | Not intervention of interest: suit therapy |
| McIntyre et al., 2012 | Not intervention of interest: constraint-induced movement therapy without VR component |
| Mentiplay et al., 2019 | Not population of interest: developmental coordination disorder |
| Metcalf et al., 2012 | Not intervention of interest: intervention to increase the physical activity of children/adolescents |
| Moreau et al., 2016 | Not intervention of interest: rehabilitation interventions to improve gait speed without VR component |
| Morgan et al., 2013 | Not intervention of interest: enriched environments (e.g., motor, social enrichment) without VR component |
| Morgan et al., 2013 | Not intervention of interest: fundamental movement skill interventions without VR component |
| Morello et al., 2019 | Not intervention of interest: multifactorial falls prevention programmes Not population of interest: older adults presenting to the emergency department with a fall |
| Mura et al., 2018 | Not population of interest: stroke, Dyslexia, Mild Cognitive Impairment, Multiple Sclerosis, Alzheimer Disease, Parkinson Disease, Down syndrome |
| Murphy & Tickle-Degnen, 2001 | Not population of interest: Parkinson Disease |
| Ni et al., 2018 | Not population of interest: Parkinson Disease |
| Ng et al., 2019 | Not population of interest: healthy participants |
| Norouzi-Gheidari et al., 2012 | Not intervention of interest: robot-assisted therapy |
| Pacheco et al., 2020 | Not population of interest: older adults without neurological conditions |
| Pearsall et al., 2014 | Not population of interest: adults with serious mental illness |
| Perrochon et al., 2019 | Not population of interest: neurological conditions, but no subgroup analysis for our population of interest was reported |
| Pogrebnoy & Dennett, 2020 | Not intervention of interest: exercise programs |
| Pope et al., 2017 | Not population of interest: young, middle-aged and older adults, no mention of any condition |
| Reedman et al., 2017 | Not intervention of interest: interventions to increase physical activity participation of children with cerebral palsy without VR component |
| Rintala et al., 2019 | Not intervention of interest: technology-based distance physical rehabilitation interventions, but no subgroup analysis for VR was reported |
| Rosa et al., 2016 | Not population of interest: stroke, Alzheimer Disease, Parkinson Disease, older adults and no sub group comparison for stroke was provided |
| Rosenbaum et al., 2014 | Not population of interest ; population too heterogeneous; Other intervention: physical activity interventions without VR component |
| Rushton et al., 2012 | Not population of interest: lumbar spinal fusion |
| Sala et al., 2018 | Not population of interest ; population too heterogeneous and type of conditions were not fully described and set as an inclusion/exclusion criteria |
| Salazar et al., 2019 | Not intervention of interest: static stretching positioning |
| Saleem et al., 2019 | Not intervention of interest: transcranial direct current stimulation |
| Schröder et al., 2019 | Not intervention of interest: repetitive gait training |
| Shi et al., 2011 | Not intervention of interest: modified constraint-induced movement therapy |
| Soares de Moura et al., 2019 | Not intervention of interest: transcranial direct current stimulation |
| Stanmore et al., 2017 | Not population of interest: healthy, Parkinson Disease, stroke, Schizophrenia, Mild Cognitive Impairment |
| Stevenson et al., 2012 | Not intervention of interest: constraint-induced movement therapy |
| Stewart et al., 2006 | Not intervention of interest: bilateral movement training without VR component |
| Stretton et al., 2017 | Not intervention of interest: interventions to improve real-world walking without VR component |
| Subramanian & Prasanna, 2018 | Not intervention of interest: VR combined with non-invasive brain stimulation |
| Tang et al., 2015 | Not intervention of interest: interventions on balance self-efficacy, VR studies were included but no subgroup analysis reported for VR |
| Tăut et al., 2017 | Not population of interest: stroke, Parkinson Disease, Acquired Brain Injury, older participants, Alzheimer Disease, neck pain, Cerebral Palsy and no subgroup analysis for stroke and/or Cerebral Palsy was reported |
| Tomlinson et al., 2012 | Not population of interest: Parkinson Disease |
| Triccas et al., 2016 | Not intervention of interest: Transcranial direct current stimulation combined with VR |
| Tripette et al., 2017 | Not population of interest: stroke, Multiple Sclerosis, Alzheimer Disease, healthy, Cerebral Palsy and no subgroup analysis for stroke and/or Cerebral Palsy was reported |
| Uhrbrand et al., 2015 | Not population of interest: Parkinson Disease |
| Valkenborghs et al., 2019 | Not intervention of interest: task-specific training, but no subgroup analysis for VR was reported |
| Välimäki et al., 2014 | Not population of interest: people with serious mental illness |
| van Duijnhoven et al., 2016 | Not intervention of interest: exercise therapy on balance capacity, but no subgroup analysis for VR was reported |
| van Criekinge et al., 2019 | Not intervention of interest: trunk training |
| Vazquez et al., 2018 | Not population of interest: healthy adults older than 44 years old |
| Veerbeek et al., 2017 | Not intervention of interest: robot-assisted therapy for the upper limb |
| Verweij et al., 2019 | Not intervention of interest: postacute multidisciplinary rehabilitation |
| Wang et al., 2016 | Not population of interest: Parkinson Disease |
| Yang et al., 2019 | Not intervention of interest: electrical stimulation therapy |
| Yilmazer et al., 2019 | Not intervention of interest: somatosensory interventions |
| Yu et al., 2018 | Not intervention of interest: motor skill interventions, but no subgroup analysis for VR was reported |
| Zhang et al., 2014 | Not intervention of interest: physical rehabilitation interventions, but no VR component |
| Zou et al., 2018 | Not intervention of interest: mindful exercises (e.g., Tai Chi, Qigong, yoga), but no VR component |

Supplementary Table S2

*AMSTAR 2 quality assessment of meta-analyses of randomized and non-randomized studies*

|  | AMSTAR 2 items^a,b,c^ | | | | | | | | | | | | | | | |
| --- | --- | --- | --- | --- | --- | --- | --- | --- | --- | --- | --- | --- | --- | --- | --- | --- |
| Author(s), year | 1 | 2 | 3 | 4 | 5 | 6 | 7 | 8 | 9 | 10 | 11 | 12 | 13 | 14 | 15 | 16 |
| Ahn et al., 2019 | Y | P Y | N | N | Y | N | Y | P Y | N | N | Y | N | N | Y | Y | Y |
| Aminov et al., 2018 | Y | P Y | Y | N | Y | Y | N | Y | Y | N | Y | N | Y | Y | Y | Y |
| Barclay et al., 2015 | Y | P Y | Y | Y | Y | Y | Y | Y | Y | Y | Y | N | Y | Y | Y | N |
| Booth et al., 2014 | N | P Y | Y | N | Y | Y | Y | Y | P Y | Y | Y | N | Y | N | N | Y |
| Chen et al., 2014 | N | P Y | N | N | N | Y | Y | P Y | N | N | Y | N | N | Y | N | Y |
| Chen et al., 2015 | Y | P Y | Y | N | Y | Y | Y | P Y | Y | N | Y | N | N | N | N | N |
| Chen et al., 2018 | Y | P Y | Y | N | Y | N | Y | Y | Y | N | Y | N | N | Y | Y | Y |
| Cheok et al., 2015 | Y | P Y | Y | N | Y | Y | Y | Y | Y | N | Y | N | N | N | N | Y |
| Corbetta et al., 2015 | Y | P Y | N | N | Y | Y | N | Y | Y | N | Y | N | N | N | N | Y |
| Da-Silva et al., 2018 | N | Y | Y | N | Y | N | Y | Y | Y | N | Y | N | N | Y | N | Y |
| De Keersmmaecker et al., 2019 | Y | Y | N | N | Y | Y | N | PY | Y | N | Y | N | N | Y | N | Y |
| De Rooij et al., 2016 | Y | P Y | N | N | Y | Y | Y | P Y | Y | N | Y | Y | Y | Y | N | N |
| Domínguez-Téllez et al., 2019a | N | PY | N | N | Y | Y | N | PY | Y | N | N | N | N | N | N | N |
| Domínguez-Téllez et al., 2019b | N | PY | N | N | Y | Y | N | PY | Y | N | N | N | N | N | N | N |
| Ferreira et al., 2020 | N | P Y | N | N | Y | N | N | P Y | Y | N | Y | N | Y | Y | Y | Y |
| García-Muñoz et al, 2019 | Y | PY | N | N | Y | Y | N | PY | Y | N | N | N | N | N | N | N |
| Ghai et al., 2019 | Y | P Y | N | N | N | N | N | P Y | Y | N | Y | Y | Y | Y | Y | Y |
| Gibbons et al., 2016 | N | P Y | N | N | Y | N | N | Y | Y | N | Y | N | Y | Y | N | N |
| Iruthayarajah et al., 2017 | Y | P Y | N | N | N | Y | N | P Y | Y | N | Y | N | N | N | N | N |
| Johansen et al., 2020 | Y | Y | N | N | Y | Y | Y | PY | Y | N | Y | Y | Y | Y | N | N |
| Karamians et al., 2019 | Y | Y | N | N | N | N | N | PY | PY | N | Y | Y | Y | Y | Y | Y |
| Laver et al., 2017 | Y | P Y | Y | Y | Y | Y | Y | Y | Y | N | Y | Y | Y | Y | Y | N |
| Lee et al., 2019 | Y | P Y | N | N | N | Y | N | P Y | Y | N | Y | Y | Y | N | Y | Y |
| Li et al., 2016 | Y | Y | N | N | Y | Y | Y | P Y | Y | N | Y | N | N | Y | N | Y |
| Lin et al., 2018 | Y | P Y | N | N | Y | N | N | Y | Y | N | Y | N | N | Y | N | N |
| Lohse et al., 2014 | Y | P Y | N | P Y | Y | Y | N | P Y | Y | N | Y | N | N | Y | Y | Y |
| Maier et al., 2019 | Y | P Y | N | N | Y | Y | Y | Y | Y | N | Y | N | N | Y | Y | N |
| Mekbib et al., 2020 | Y | N | N | N | N | Y | N | PY | Y | N | Y | N | N | Y | N | Y |
| Mohammadi et al., 2019 | Y | Y | N | N | Y | N | N | P Y | Y | N | Y | N | N | Y | N | Y |
| Prosperini et al., 2020 | Y | Y | N | N | Y | Y | N | Y | Y | N | Y | Y | Y | Y | Y | Y |
| Rodrigues-Baroni et al., 2014 | Y | N | Y | N | N | N | Y | Y | Y | N | Y | N | N | Y | N | N |
| Rutkowski et al., 2020 | N | PY | N | N | Y | Y | N | PY | Y | N | Y | N | N | Y | N | Y |
| Saposnik et al., 2011 | N | N | N | N | Y | N | N | P Y | N | N | Y | N | N | Y | Y | N |
| Saywell et al., 2017 | Y | P Y | N | N | Y | Y | Y | P Y | Y | N | Y | N | N | N | N | Y |
| Tay et al., 2018 | N | P Y | N | N | N | Y | Y | Y | Y | N | Y | N | Y | Y | N | Y |
| Veerbeek et al., 2014 | Y | P Y | N | N | N | N | N | N | Y | N | Y | N | N | Y | N | Y |
| Wang et al., 2015 | Y | P Y | N | N | Y | Y | N | P Y | Y | N | Y | N | N | N | Y | Y |
| Warnier et al., 2019 | N | PY | N | N | Y | Y | Y | PY | Y | N | N | N | N | N | N | Y |
| Wattchow et al., 2018 | Y | Y | N | N | Y | N | Y | N | Y | N | Y | N | N | N | N | Y |
| Wiley et al., 2020 | Y | PY | N | N | Y | Y | N | Y | Y | N | N | N | N | N | N | Y |
| Wu et al., 2019 | Y | PY | N | N | Y | Y | N | PY | Y | N | Y | N | N | Y | Y | Y |

*Note.* ^a^ Y = Yes; N =No; P Y = Partial Yes; ^b^ AMSTAR 2 critical domains = Item 2, Item 4, Item 7, Item 9, Item 11, Item 13, Item 15; ^c^ AMSTAR 2 Items:

1. Did the research questions and inclusion criteria for the review include the components of PICO?

2. Did the report of the review contain an explicit statement that the review methods were established prior to the conduct of the review and did the report justify any significant deviations from the protocol?

3. Did the review authors explain their selection of the study designs for inclusion in the review?

4. Did the review authors use a comprehensive literature search strategy?

5. Did the review authors perform study selection in duplicate?

6. Did the review authors perform data extraction in duplicate?

7. Did the review authors provide a list of excluded studies and justify the exclusions?

8. Did the review authors describe the included studies in adequate detail?

9. Did the review authors use a satisfactory technique for assessing the risk of bias (RoB) in individual studies that were included in the review?

10. Did the review authors report on the sources of funding for the studies included in the review?

11. If meta-analysis was performed, did the review authors use appropriate methods for statistical combination of results?

12. If meta-analysis was performed, did the review authors assess the potential impact of RoB in individual studies on the results of the meta-analysis or other evidence synthesis?

13. Did the review authors account for RoB in primary studies when interpreting/discussing the results of the review?

14. Did the review authors provide a satisfactory explanation for, and discussion of, any heterogeneity observed in the results of the review?

15. If they performed quantitative synthesis did the review authors carry out an adequate investigation of publication bias (small study bias) and discuss its likely impact on the results of the review?

16. Did the review authors report any potential sources of conflict of interest, including any funding they received for conducting the review?

Supplementary Table S3

*Risk of bias for the included studies*

| Author(s), year | Risk of bias measurement instrument | Risk of bias reported in the meta-analysis | Overall risk of bias reported in the meta-analysis | Summary of trial limitations (risk of  bias reported in percentages) |
| --- | --- | --- | --- | --- |
| Ahn et al., 2019 | Jadad Scale | **Random sequence generation:** 1 RCT low risk; 8 RCTs high risk  **Blinding of outcome assessment:** 3 RCTs low risk; 6 RCTs high risk  **Incomplete outcome data:** 7 RCTs low risk; 2 RCTs high risk | "Three studies scored 1–4 of the maximum score of 4 points." | **Random sequence generation:** 11% RCT low risk; 89% RCTs high risk  **Blinding of outcome assessment:** 33% RCTs low risk; 66% RCTs high risk  **Incomplete outcome data:** 78% RCTs low risk; 22% RCTs high risk |
| Aminov et al., 2018 | Physiotherapy Evidence  Database (PEDro) Scale | **Random sequence generation:** 30 RCTs low risk; 1 RCT high or unclear risk  **Allocation concealment:** 10 RCTs low risk; 21 RCTs high or unclear risk  **Blinding of participants:** 1 RCT low risk; 30 RCTs high risk  **Blinding of personnel:** 31 RCTs high risk  **Blinding of outcome assessment:** 19 RCTs low risk; 12 RCTs high or unclear risk  **Incomplete outcome data:** 26 RCTs low risk; 5 RCTs high or unclear risk  **Group similarity at baseline:** 25 RCTs low risk; 6 RCTs high or unclear risk  **Intention to treat:** 16 RCTs low risk; 15 high or unclear risk | "The methodological quality of included studies was generally high." | **Random sequence generation:** 97% RCTs low risk; 3% RCTs high or unclear risk  **Allocation concealment:** 32% RCTs low risk; 68% RCTs high or unclear risk  **Blinding of participants:** 3% RCTs low risk; 97% RCTs high risk  **Blinding of personnel:** 100% RCTs high risk  **Blinding of outcome assessment:** 61% RCTs low risk; 39% RCTs high or unclear risk  **Incomplete outcome data:** 84% RCTs low risk; 16% RCTs high or unclear risk  **Group similarity at baseline:** 81% RCTs low risk; 19% RCTs high or unclear risk  **Intention to treat:** 52% RCTs low risk; 48% high or unclear risk |
| Barclay et al., 2015 | Cochrane’s “Risk of bias” tool | **Random sequence generation:** 1 RCT unclear risk  **Allocation concealment:** 1 RCT low risk  **Blinding of participants:** 1 RCT high risk  **Blinding of personnel:** 1 RCT high risk  **Blinding of outcome assessment:** 1 RCT low risk  **Incomplete outcome data:** 1 RCT high risk  **Selective reporting:** 1 RCT low risk | ^a^ | **Random sequence generation:** 100% RCTs unclear risk  **Allocation concealment:** 100% RCTs low risk  **Blinding of participants:** 100% RCTs high risk  **Blinding of personnel:** 100% RCTs high risk  **Blinding of outcome assessment:** 100% RCTs low risk  **Incomplete outcome data:** 100% RCTs high risk  **Selective reporting:** 100% RCTs low risk |
| Booth et al., 2014 | Joanna Briggs Institute appraisal  tool | **Random sequence generation:** 1 RCT low risk  **Allocation concealment:** 1 RCT unclear risk  **Blinding of participants:** 1 RCT high risk  **Blinding of outcome assessment:** 1 RCT low risk  **Incomplete outcome data:** 1 RCT high risk  **Group similarity at baseline:** 1 RCT low risk | ^a^ | **Random sequence generation:** 100% RCTs low risk  **Allocation concealment:** 100% RCTs unclear risk  **Blinding of participants:** 100% RCTs high risk  **Blinding of outcome assessment:** 100% RCTs low risk  **Incomplete outcome data:** 100% RCTs high risk  **Group similarity at baseline:** 100% RCTs low risk |
| Chen et al., 2014 | “Similar adapted scoring protocol, which was used by Huang et al. (2009) to evaluate case series or single-subject design articles. This scoring system consisted of 11 items with categories on study population, design, blinding procedure, measurement instruments, control of cointervention, control for dose of therapy, and appropriate statistical analysis.” | ^a^ | “In general, the methodological quality of articles that investigated VR effect was poor to fair.” | ^a^ |
| Chen et al., 2015 | Cochrane’s “Risk of bias” tool | ^a^ | 3 RCTs low risk; 1 RCT unclear risk | ^a^ |
| Chen et al., 2018 | Physiotherapy Evidence  Database (PEDro) Scale | ^a^ | “The PEDro quality  scores for the studies ranged from 4 to 8 (4 studies with a score of 4, 4 studies with a score of 5, 3 studies with a score of 6, 3 studies with a score of 7, and 5 studies with a score of 8), indicating  that the quality of the RCTs included in this meta-analysis was fair to good.” | ^a^ |
| Cheok et al., 2015 | Physiotherapy Evidence  Database (PEDro) Scale | **Random sequence generation:** 5 RCTs low risk; 1 RCT unclear risk  **Allocation concealment:** 3 RCTs low risk; 2 RCTs unclear risk; 1 RCTs high risk  **Blinding of participants and personnel:** 5 RCTs unclear risk; 1 RCT high risk  **Blinding of outcome assessment:** 4 RCTs low risk; 2 RCTs unclear risk  **Incomplete outcome data:** 1 RCT low risk; 2 RCTs unclear risk; 3 RCTs high risk  **Group similarity at baseline:** 4 RCTs low risk; 2 RCTs unclear risk  **Selective reporting:** 6 RCTs low risk  **Co interventions constant:** 6 RCTs low risk | “All included studies have  a degree of performance bias due to the difficulty in blinding participants and therapists to group allocation. The median PEDro score was 6/10 trials (range 3 to 8).” | **Random sequence generation:** 83% RCTs low risk; 17% RCTs unclear risk  **Allocation concealment:** 50% RCTs low risk; 33% RCTs unclear risk; 17% RCTs high risk  **Blinding of participants and personnel:** 83% RCTs unclear risk; 17% RCT high risk  **Blinding of outcome assessment:** 67% RCTs low risk; 33% RCTs unclear risk  **Incomplete outcome data:** 17% RCT low risk; 33% RCTs unclear risk; 50% RCTs high risk  **Group similarity at baseline:** 67% RCTs low risk; 33% RCTs unclear risk  **Selective reporting:** 100% RCTs low risk  **Co interventions constant:** 100% RCTs low risk |
| Corbetta et al., 2015 | Cochrane’s “Risk of bias” tool | **Random sequence generation:** 12 RCTs low risk; 3 RCTs unclear risk  **Allocation concealment:** 6 RCTs low risk; 8 RCTs unclear risk; 1 RCTs high risk  **Blinding of outcome assessment:** 12 RCTs low risk; 3 RCTs unclear risk  **Selective reporting:** 15 RCTs low risk | “The quality of the trials was good.” | **Random sequence generation:** 80% RCTs low risk; 20% RCTs unclear risk  **Allocation concealment:** 40% RCTs low risk; 53% RCTs unclear risk; 7% RCTs high risk  **Blinding of outcome assessment:** 80% RCTs low risk; 20% RCTs unclear risk  **Selective reporting:** 100% RCTs low risk |
| Da-Silva et al., 2018 | Cochrane’s “Risk of bias” tool | **Random sequence generation:** 2 RCTs low risk  **Allocation concealment:** 2 RCTs low risk  **Blinding of participants and personnel:** 2 RCTs high risk  **Blinding of outcome assessment:** 2 RCTs unclear risk  **Incomplete outcome data:** 1 RCT low risk; 1 RCT high risk  **Selective reporting:** 1 RCTs low risk; 1 RCT unclear risk | ^a^ | **Random sequence generation:** 100% RCTs low risk  **Allocation concealment:** 100% RCTs low risk  **Blinding of participants and personnel:** 100% RCTs high risk  **Blinding of outcome assessment:** 100% RCTs unclear risk  **Incomplete outcome data:** 50% RCTs low risk; 50% RCT high risk  **Selective reporting:** 50% RCTs low risk; 50% RCTs unclear risk |
| De Keersmmaecker et al., 2019 | Cochrane’s “Risk of bias” tool | **Random sequence generation:** 3 RCTs low risk; 5 RCTs unclear risk; 1 RCT n/a  **Allocation concealment:** 6 RCTs low risk; 2 RCTs unclear risk; 1 RCT n/a  **Blinding of participants and personnel:** 8 RCTs low risk; 1 RCT n/a  **Blinding of outcome assessment:** 7 RCTs low risk; 2 RCTs unclear risk  **Incomplete outcome data:** 7 RCTs low risk; 1 RCT unclear risk; 1 RCT high risk  **Selective reporting:** 2 RCTs low risk; 7 RCTs unclear risk | “Only 3 studies provided a  preregistered protocol.” | **Random sequence generation:** 33% RCTs low risk; 56% RCTs unclear risk; 11% RCT n/a  **Allocation concealment:** 67% RCTs low risk; 22% RCTs unclear risk; 11% RCT n/a  **Blinding of participants and personnel:** 89% RCTs low risk; 11% RCT n/a  **Blinding of outcome assessment:** 78% RCTs low risk; 22% RCTs unclear risk  **Incomplete outcome data:** 78% RCTs low risk; 11% RCT unclear risk; 11% RCT high risk  **Selective reporting:** 22% RCTs low risk; 78% RCTs unclear risk |
| De Rooij et al., 2016 | Physiotherapy Evidence  Database (PEDro) Scale | **Random sequence generation:** 21 RCTs low risk  **Allocation concealment:** 8 RCTs low risk; 13 RCTs high risk  **Blinding of participants:** 1 RCT low risk; 20 RCTs high risk  **Blinding of personnel:** 21 RCTs high risk  **Blinding of outcome assessment:** 13 RCTs low risk; 8 RCTs high risk  **Incomplete outcome data:** 16 RCTs low risk; 5 RCTs high risk  **Group similarity at baseline:** 18 RCTs low risk; 3 RCTs high risk  **Intention to treat:** 9 RCTs low risk, 12 RCTs high risk | “The PEDro scores of the included studies varied between 3 and 8, with a median of 6.0 and an interquartile range of 2.0. Thirteen studies had a score of 6 or higher and were considered of high  quality.” | **Random sequence generation:** 100% RCTs low risk  **Allocation concealment:** 38% RCTs low risk; 62% RCTs high risk  **Blinding of participants:** 5% RCT low risk; 95% RCTs high risk;  **Blinding of personnel:** 100% RCTs high risk  **Blinding of outcome assessment:** 62% RCTs low risk; 38% RCTs high risk  **Incomplete outcome data:** 76% RCTs low risk; 24% RCTs high risk  **Group similarity at baseline:** 86% RCTs low risk; 14% RCTs high risk  **Intention to treat:** 43% RCTs low risk, 57% RCTs high risk |
| Domínguez-Téllez et al., 2019a | Physiotherapy Evidence  Database (PEDro) Scale | ^a^ | “After evaluating the studies with the PEDro scale, it is worth highlighting that, of the 14 studies included in the review, seven have a high methodological quality (≥ 6 points).” | ^a^ |
| Domínguez-Téllez et al., 2019b | Physiotherapy Evidence  Database (PEDro) Scale | ^a^ | “The methodological quality of the included studies was  generally high (average total PEDro score = 6.25, range 4–  10).” | ^a^ |
| Ferreira et al., 2020 | Physiotherapy Evidence  Database (PEDro) Scale | **Random sequence generation:** 10 RCTs low risk; 1 RCT high risk  **Allocation concealment:** 7 RCTs low risk; 4 RCTs high risk  **Blinding of participants:** 1 RCT low risk; 10 RCTs high risk  **Blinding of personnel:** 11 RCTs high risk  **Blinding of outcome assessment:** 8 RCTs low risk; 3 RCTs high risk  **Incomplete outcome data:** 8 RCTs low risk; 3 RCTs high risk  **Group similarity at baseline:** 10 RCTs low risk; 1 RCT high risk  **Intention to treat:** 4 RCTs low risk, 7 RCTs high risk | “The mean score of the methodological quality of the studies evaluated  using the PEDro Scale was 6.2 + 1.9, with only one study achieving a score  of 3, two studies scoring 4, one study scoring 5, three studies scoring 7, and  four studies scoring 8.” | **Random sequence generation:** 91% RCTs low risk; 9% RCTs high risk  **Allocation concealment:** 64% RCTs low risk; 36% RCTs high risk  **Blinding of participants:** 9% RCT low risk; 91% RCTs high risk  **Blinding of personnel:** 100% RCTs high risk  **Blinding of outcome assessment:** 73% RCTs low risk; 27% RCTs high risk  **Incomplete outcome data:** 73% RCTs low risk; 27% RCTs high risk  **Group similarity at baseline:** 91% RCTs low risk; 9% RCT high risk  **Intention to treat:** 36% RCTs low risk, 64% RCTs high risk |
| García-Muñoz et al, 2019 | Physiotherapy Evidence  Database (PEDro) Scale | **Random sequence generation:** 6 RCTs low risk  **Allocation concealment:** 5 RCTs low risk; 1 RCT high risk  **Blinding of participants:** 6 RCT low risk  **Blinding of personnel:** 6 RCTs high risk  **Blinding of outcome assessment:** 4 RCTs low risk; 2 RCTs high risk  **Incomplete outcome data:** 6 RCTs low risk  **Group similarity at baseline:** 6 RCTs low risk  **Intention to treat:** 3 RCTs low risk, 3 RCTs high risk | “Eleven studies showed methodological quality 'good' (evidence level I), five, methodological quality 'fair' (level II evidence). All studies were described as randomized, and in most cases the allocation was hidden. However, neither the subjects nor the therapists were blinded in any case.” | **Random sequence generation:** 100% RCTs low risk  **Allocation concealment:** 83% RCTs low risk; 17% RCT high risk  **Blinding of participants:** 100% RCT low risk  **Blinding of personnel:** 100% RCTs high risk  **Blinding of outcome assessment:** 67% RCTs low risk; 33% RCTs high risk  **Incomplete outcome data:** 100% RCTs low risk  **Group similarity at baseline:** 100% RCTs low risk  **Intention to treat:** 50% RCTs low risk, 50% RCTs high risk |
| Ghai et al., 2019 | Physiotherapy Evidence  Database (PEDro) Scale | **Random sequence generation:** 14 RCTs low risk  **Allocation concealment:** 4 RCTs low risk; 10 RCTs high risk  **Blinding of participants:** 5 RCT low risk; 9 RCTs high risk  **Blinding of personnel:** 4 RCTs low risk; 10 RCTs high risk  **Blinding of outcome assessment:** 5 RCTs low risk; 9 RCTs high risk  **Incomplete outcome data:** 10 RCTs low risk; 4 RCTs high risk  **Group similarity at baseline:** 10 RCTs low risk  **Intention to treat:** 14 RCTs high risk | “The average PEDro score of the 18 included studies was  computed to be (M ± S.D). 5.7 ± 1.4 out of 10, indicating, on  average, a “good” quality of the studies. Here, one study scored  9, one study scored 8, three scored 7, two  scored 6, six scored 5, and three studies  scored 4.” | **Random sequence generation:** 100% RCTs low risk  **Allocation concealment:** 29% RCTs low risk; 71% RCTs high risk  **Blinding of participants:** 36% RCT low risk; 64% RCTs high risk  **Blinding of personnel:** 29% RCTs low risk; 71% RCTs high risk  **Blinding of outcome assessment:** 36% RCTs low risk; 64% RCTs high risk  **Incomplete outcome data:** 71% RCTs low risk; 29% RCTs high risk  **Group similarity at baseline:** 100% RCTs low risk  **Intention to treat:** 100% RCTs high risk |
| Gibbons et al., 2016 | Physiotherapy Evidence  Database (PEDro) Scale | **Random sequence generation:** 18 RCTs low risk; 2 RCTs high risk  **Allocation concealment:** 7 RCTs low risk; 13 RCTs high risk  **Blinding of participants:** 2 RCTs low risk; 18 RCTs high risk  **Blinding of personnel:** 20 RCTs high risk  **Blinding of outcome assessment:** 10 RCTs low risk; 10 RCTs high risk  **Incomplete outcome data:** 10 RCTs low risk; 10 RCTs high risk  **Group similarity at baseline:** 19 RCTs low risk; 1 RCT high risk  **Intention to treat:** 1 RCT low risk, 19 RCTs high risk | “PEDro scores ranged from 3 to 8, with a mean of 5.2.  Given the nature of the interventions none of the studies satisfied the criteria of blinding therapists and participants, therefore  the most likely source of bias. The second most frequent unsatisfed criteria was attrition bias, meaning that in many cases there is a potential that people who did not complete the study would have responded to treatment differently to the ones that completed the study, therefore potentially  affecting results.” | **Random sequence generation:** 90% RCTs low risk; 10% RCTs high risk  **Allocation concealment:** 35% RCTs low risk; 65% RCTs high risk  **Blinding of participants:** 10% RCTs low risk; 90% RCTs high risk  **Blinding of personnel:** 100% RCTs high risk  **Blinding of outcome assessment:** 50% RCTs low risk; 50% RCTs high risk  **Incomplete outcome data:** 50% RCTs low risk; 50% RCTs high risk  **Group similarity at baseline:** 95% RCTs low risk; 5% RCT high risk  **Intention to treat:** 5% RCT low risk, 95% RCTs high risk |
| Iruthayarajah et al., 2017 | Physiotherapy Evidence  Database (PEDro) Scale | ^a^ | “PEDro scores ranged  from 5 to 8.” Good methodological quality for 15 studies and fair methodological quality for two studies. | ^a^ |
| Johansen et al., 2020 | Cochrane’s “Risk of bias” tool | **Random sequence generation:** 4 RCTs low risk; 2 RCTs unclear risk; 1 RCT high risk  **Allocation concealment:** 3 RCTs low risk; 2 RCTs unclear risk; 2 RCT high risk  **Blinding of participants and personnel:** 6 RCTs low risk; 1 RCT high risk  **Blinding of outcome assessment:** 4 RCTs low risk; 1 RCT unclear risk; 2 RCTs high risk  **Incomplete outcome data:** 5 RCTs low risk; 1 RCT unclear risk; 1 RCT high risk  **Selective reporting:** 4 RCTs low risk; 3 RCTs high risk | “Overall, there were many unclear and high risk of  bias factors due to lack of reporting.” | **Random sequence generation:** 57% RCTs low risk; 29% RCTs unclear risk; 14% RCT high risk  **Allocation concealment:** 44% RCTs low risk; 28% RCTs unclear risk; 28% RCT high risk  **Blinding of participants and personnel:** 86% RCTs low risk; 14% RCT high risk  **Blinding of outcome assessment:** 57% RCTs low risk; 14% RCT unclear risk; 29% RCTs high risk  **Incomplete outcome data:** 72% RCTs low risk; 14% RCT unclear risk; 14% RCTs high risk  **Selective reporting:** 57% RCTs low risk; 43% RCTs high risk |
| Karamians et al., 2019 | Downs-Black rating scale items | **Random sequence generation:** 26 RCTs low risk; 12 RCTs high risk  **Blinding of outcome assessment:** 31 RCTs low risk; 7 RCTs high risk  **Intention to treat:** 20 RCTs low risk; 18 RCTs high risk | ^a^ | **Random sequence generation:** 68% RCTs low risk; 32% RCTs high risk  **Blinding of outcome assessment:** 82% RCTs low risk; 18% RCTs high risk  **Intention to treat:** 53% RCTs low risk; 47% RCTs high risk |
| Laver et al., 2017 | Cochrane’s “Risk of bias” tool | **Random sequence generation:** 34 RCTs low risk; 12 RCTs unclear risk; 4 RCTs high risk  **Allocation concealment:** 26 RCTs low risk; 21 RCTs unclear risk; 3 RCTs high risk  **Blinding of outcome assessment:** 41 RCTs low risk; 6 RCTs unclear risk; 3 RCTs high risk  **Incomplete outcome data:** 32 RCTs low risk; 13 RCTs unclear risk; 5 RCTs high risk  **Selective reporting:** 25 RCTs low risk; 24 RCTs unclear risk; 1 RCT high risk | ^a^ | **Random sequence generation:** 68% RCTs low risk; 24% RCTs unclear risk; 8% RCTs high risk  **Allocation concealment:** 52% RCTs low risk; 42% RCTs unclear risk; 6% RCTs high risk  **Blinding of outcome assessment:** 82% RCTs low risk; 12% RCTs unclear risk; 6% RCTs high risk  **Incomplete outcome data:** 64% RCTs low risk; 26% RCTs unclear risk; 10% RCTs high risk  **Selective reporting:** 50% RCTs low risk; 48% RCTs unclear risk; 2% RCT high risk |
| Lee et al., 2019 | Physiotherapy Evidence  Database (PEDro) Scale | **Random sequence generation:** 20 RCTs low risk; 1 RCT high risk  **Allocation concealment:** 9 RCTs low risk; 12 RCTs high risk  **Blinding of participants:** 3 RCTs low risk; 18 RCTs high risk  **Blinding of personnel:** 2 RCTs low risk; 19 RCTs high risk  **Blinding of outcome assessment:** 13 RCTs low risk; 8 RCTs high risk  **Incomplete outcome data:** 14 RCTs low risk; 7 RCTs high risk  **Group similarity at baseline:** 20 RCTs low risk; 1 RCT high risk  **Intention to treat:** 6 RCTs low risk; 15 high risk | ^a^ | **Random sequence generation:** 95% RCTs low risk; 5% RCT high risk  **Allocation concealment:** 43% RCTs low risk; 57% RCTs high risk  **Blinding of participants:** 14% RCTs low risk; 86% RCTs high risk  **Blinding of personnel:** 10% RCTs low risk; 90% RCTs high risk  **Blinding of outcome assessment:** 62% RCTs low risk; 38% RCTs high risk  **Incomplete outcome data:** 67% RCTs low risk; 33% RCTs high risk  **Group similarity at baseline:** 95% RCTs low risk; 5% RCT high risk  **Intention to treat:** 29% RCTs low risk; 71% high risk |
| Li et al., 2016 | Cochrane’s “Risk of bias” tool | ^a^ | “Quality of studies was assessed using the criteria  recommended in the Cochrane Collaboration’s  ‘Risk of bias’ tool. The criteria included: (1) random sequence generation; (2) allocation concealment; (3) blinding of participants and personnel;  (4) blinding of outcome assessment; (5) incomplete  outcome data and selective reporting. For each criteria met, a score of 1 was allocated. A score of 3 or  above meant a good study. The methodological quality of the  included studies was modest.”  11 RCTs have good quality; 4 RCTs have poor quality; scores ranged from 2 to 4 | ^a^ |
| Lin et al., 2018 | Physiotherapy Evidence  Database (PEDro) Scale | ^a^ | “The PEDro scale (0-10 points with a higher score indicating higher quality) is used to assess the risk of bias and the quality of  the included studies. The scale includes 10 items regarding random allocation, blinding procedures, and the dropout rate. Quality was classified as high (6-10), fair (4-5), and poor (≤ 3). To make recommendations based on a high level of evidence, this review included only trials with a PEDro score ≥ 4.” | ^a^ |
| Lohse et al., 2014 | Physiotherapy Evidence  Database (PEDro) Scale | **Random sequence generation:** 20 RCTs low risk; 4 RCT high or unclear risk  **Allocation concealment:** 9 RCTs low risk; 15 RCTs high or unclear risk  **Blinding of participants:** 5 RCTs low risk; 19 RCTs high or unclear risk  **Blinding of personnel:** 1 RCT low risk; 23 RCTs high or unclear risk  **Blinding of outcome assessment:** 14 RCTs low risk; 10 RCTs high or unclear risk  **Incomplete outcome data:** 19 RCTs low risk; 5 RCTs high or unclear risk  **Group similarity at baseline:** 15 RCTs low risk; 9 RCTs high or unclear risk  **Intention to treat:** 4 RCTs low risk, 20 high or unclear risk | “PEDro scores for the various studies were moderate, with a mean of 5.42 and SD of 1.60. Areas of weakness across studies were concealment of participant allocation (34.6%), blinding of participants (19.2%) and  therapists (3.8%) to conditions and following an intention to treat (ITT) analysis (19.2%). Proper concealment and ITT analysis are particularly important considerations; studies may have actually  fulfilled these criteria but lacked explicit description in their Methods sections. Lack of blinding for both participants and therapists was also a limitation of the studies. Although it is not  feasible to truly "blind" participants to the fact that they are receiving VR therapy, keeping patients and therapists naive to the  experimental hypotheses would be a useful step to add experimental rigour and should be reported if it was achieved.” | **Random sequence generation:** 83% RCTs low risk; 17% RCT high or unclear risk  **Allocation concealment:** 38% RCTs low risk; 62% RCTs high or unclear risk  **Blinding of participants:** 20% RCTs low risk; 80% RCTs high or unclear risk  **Blinding of personnel:** 4% RCT low risk; 96% RCTs high or unclear risk  **Blinding of outcome assessment:** 59% RCTs low risk; 41% RCTs high or unclear risk  **Incomplete outcome data:** 79% RCTs low risk; 21% RCTs high or unclear risk  **Group similarity at baseline:** 62% RCTs low risk; 38% RCTs high or unclear risk  **Intention to treat:** 17% RCTs low risk, 83% high or unclear risk |
| Maier et al, 2019 | Cochrane’s “Risk of bias” tool | **Random sequence generation:** 25 RCTs low risk; 4 RCT unclear risk; 1 RCT at high risk  **Allocation concealment:** 12 RCTs low risk; 16 RCTs at unclear risk; 2 RCTs high risk  **Blinding of participants and personnel:** 5 RCT low risk; 16 RCTs unclear risk; 9 RCTs high risk  **Blinding of outcome assessment:** 25 RCTs low risk; 4 RCTs unclear risk; 1 RCTs high risk  **Incomplete outcome data:** 23 RCTs low risk; 5 RCTs unclear risk; 2 RCTs high risk  **Selective reporting:** 29 RCTs low risk; 1 RCT high risk | ^a^ | **Random sequence generation:** 84% RCTs low risk; 13% RCT unclear risk; 3% RCT at high risk  **Allocation concealment:** 40% RCTs low risk; 53% RCTs at unclear risk; 7% RCTs high risk  **Blinding of participants and personnel:** 17% RCT low risk; 53% RCTs unclear risk; 30% RCTs high risk  **Blinding of outcome assessment:** 83% RCTs low risk; 13% RCTs unclear risk; 3% RCTs high risk  **Incomplete outcome data:** 76% RCTs low risk; 17% RCTs unclear risk; 7% RCTs high risk  **Selective reporting:** 97% RCTs low risk; 3% RCT high risk |
| Mekbib et al., 2020 | Physiotherapy Evidence  Database (PEDro) Scale | **Random sequence generation:** 27 RCTs low risk  **Allocation concealment:** 13 RCTs low risk; 14 RCTs high or unclear risk  **Blinding of participants:** 27 RCTs high or unclear risk  **Blinding of personnel:** 27 RCTs high or unclear risk  **Blinding of outcome assessment:** 23 RCTs low risk; 7 RCTs high or unclear risk  **Incomplete outcome data:** 20 RCTs low risk; 7 RCTs high or unclear risk  **Group similarity at baseline:** 22 RCTs low risk; 5 RCTs high or unclear risk  **Intention to treat:** 11 RCTs low risk, 16 high or unclear risk | “The overall quality of the included studies was high (average total PEDro score of 6.29).” | **Random sequence generation:** 100% RCTs low risk  **Allocation concealment:** 47% RCTs low risk; 53% RCTs high or unclear risk  **Blinding of participants:** 100% RCTs high or unclear risk  **Blinding of personnel:** 100% RCTs high or unclear risk  **Blinding of outcome assessment:** 85% RCTs low risk; 15% RCTs high or unclear risk  **Incomplete outcome data:** 74% RCTs low risk; 26% RCTs high or unclear risk  **Group similarity at baseline:** 81% RCTs low risk; 19% RCTs high or unclear risk  **Intention to treat:** 41% RCTs low risk, 59% high or unclear risk |
| Mohammadi et al., 2019 | Physiotherapy Evidence  Database (PEDro) Scale | **Random sequence generation:** 13 RCTs low risk  **Allocation concealment:** 11 RCTs low risk; 2 RCTs high risk  **Blinding of participants:** 1 RCT low risk; 12 RCTs high risk  **Blinding of personnel:** 1 RCT low risk; 12 RCTs high risk  **Blinding of outcome assessment:** 8 RCTs low risk; 5 RCTs high risk  **Incomplete outcome data:** 13 RCTs low risk  **Group similarity at baseline:** 13 RCTs low risk  **Intention to treat:** 10 RCTs low risk, 3 high risk | PedRo  “The studies were overall of high quality with a mean of 7.42 (SD .98) and scores ranging from 5 to 9 out of 10. There was only 1 low quality study with a score of 5 out of 10.” | **Random sequence generation:** 100% RCTs low risk  **Allocation concealment:** 85% RCTs low risk; 15% RCTs high risk  **Blinding of participants:** 8% RCT low risk; 92% RCTs high risk  **Blinding of personnel:** 8% RCT low risk; 92% RCTs high risk  **Blinding of outcome assessment:** 62% RCTs low risk; 38% RCTs high risk  **Incomplete outcome data:** 100% RCTs low risk  **Group similarity at baseline:** 100% RCTs low risk  **Intention to treat:** 77% RCTs low risk, 23% high risk |
| Prosperini et al., 2020 | Physiotherapy Evidence  Database (PEDro) Scale | ^a^ | “Approximately, 75% of included studies (*n* = 31) were of high quality (rating ≥ 6/10 on the PEDro scale), nine studies were of fair quality (rating 4–5/10 on the PEDro scale), and only one study was of poor quality (rating 3/10 on the PEDro scale).” | ^a^ |
| Rodrigues-Baroni et al., 2014 | Physiotherapy Evidence  Database (PEDro) Scale | **Random sequence generation:** 7 RCTs low risk  **Allocation concealment:** 4 RCTs low risk; 3 RCTs high risk  **Blinding of participants:** 1 RCTs low risk; 5 RCTs high risk  **Blinding of personnel:** 7 RCTs high risk  **Blinding of outcome assessment:** 6 RCTs low risk; 1 RCTs high risk  **Incomplete outcome data:** 4 RCTs low risk; 3 RCTs high risk  **Group similarity at baseline:** 6 RCTs low risk  **Intention to treat:** 1 RCT low risk, 6 high risk | “A source of bias in the included trials was lack of  blinding of therapists and participants, since it is  very diffcult or unpractical to blind either during  the delivery of complex interventions, such as walking training. In addition, the majority of the  included trials did not report whether an intentionto-treat analysis was carried-out. On the other hand, the mean PEDro score of 6.1 for the included trials indicated good methodological quality.” | **Random sequence generation:** 100% RCTs low risk  **Allocation concealment:** 57% RCTs low risk; 43% RCTs high risk  **Blinding of participants:** 14% RCTs low risk; 86% RCTs high risk  **Blinding of personnel:** 100% RCTs high risk  **Blinding of outcome assessment:** 86% RCTs low risk; 14% RCTs high risk  **Incomplete outcome data:** 57% RCTs low risk; 43% RCTs high risk  **Group similarity at baseline:** 100% RCTs low risk  **Intention to treat:** 14% RCT low risk, 86% high risk |
| Rutkowski et al., 2020 | Cochrane’s “Risk of bias” tool | **Random sequence generation:** 6 RCTs low risk; 4 RCTs high risk  **Allocation concealment:** 5 RCTs low risk; 5 RCTs high risk  **Blinding of outcome assessment:** 9 RCTs low risk; 1 RCTs high risk  **Incomplete outcome data:** 7 RCT low risk; 3 RCT high risk  **Selective reporting:** 9 RCTs low risk; 1 RCT high risk | ^a^ | **Random sequence generation:** 60% RCTs low risk; 40% RCTs high risk  **Allocation concealment:** 50% RCTs low risk; 50% RCTs high risk  **Blinding of outcome assessment:** 90% RCTs low risk; 10% RCTs high risk  **Incomplete outcome data:** 70% RCT low risk; 30% RCT high risk  **Selective reporting:** 90% RCTs low risk; 10% RCT high risk |
| Saposnik et al., 2011 |  | ^a^ | ^a^ | ^a^ |
| Saywell et al., 2017 | Cochrane’s “Risk of bias” tool | **Random sequence generation:** 13 RCTs low risk; 10 RCTs high risk  **Allocation concealment:** 6 RCTs low risk; 17 RCTs high risk  **Blinding of participants and personnel:** 5 RCTs low risk; 18 RCTs high risk  **Blinding of outcome assessment:** 17 RCTs low risk; 6 RCTs high risk  **Incomplete outcome data:** 11 RCT low risk; 12 RCT high risk  **Selective reporting:** 2 RCTs low risk; 21 RCT high risk  **Other sources of bias:** 22 RCTs low risk; 1 RCT high (within group design) | “Thirteen studies were considered to be of high  methodological quality, with scores ranging between 21 and 25. The remainder were considered to be of moderate quality, ranging between 15 and 20. Most studies clearly described their hypothesis, functional outcomes measures, and adopted measure that have  been reported as being valid and reliable. Five studies failed to report on compliance with the  intervention/therapy and four blinded participants to the intervention by ensuring  participants were unaware of the intent and purpose of the study.” | **Random sequence generation:** 57% RCTs low risk; 43% RCTs high risk  **Allocation concealment:** 26% RCTs low risk; 76% RCTs high risk  **Blinding of participants and personnel:** 22% RCTs low risk; 78% RCTs high risk  **Blinding of outcome assessment:** 74% RCTs low risk; 26% RCTs high risk  **Incomplete outcome data:** 48% RCT low risk; 52% RCT high risk  **Selective reporting:** 9% RCTs low risk; 91% RCT high risk  **Other sources of bias:** 96% RCTs low risk; 4% RCT high (within group design) |
| Tay et al., 2018 | Cochrane’s “Risk of bias” tool | **Random sequence generation:** 8 RCTs low risk; 6 RCTs unclear risk; 3 RCTs high risk  **Selective reporting:** 17 RCTs low risk | “Half of the studies reviewed reported the sequence generation but allocation concealment was not explicitly described. Most of the studies did not blind the participants nor the therapists delivering the intervention. Outcomes assessors were blinded instead in these studies except for the study by Hung et al. Three  studies were evaluated to have high risk of selection bias for matching parameters before randomization or randomized participants according to admission period. Hung et al. [36] excluded patients who  could not complete 6 weeks of training hence may contribute to other bias such as higher adherence rate.” | **Random sequence generation:** 47% RCTs low risk; 35% RCTs unclear risk; 18% RCTs high risk  **Selective reporting:** 100% RCTs low risk |
| Veerbeek et al., 2014 | Physiotherapy Evidence  Database (PEDro) Scale | ^a^ | “PEDro score range 5 to 7 and 3 to 8. Our quantitative analyses only included RCTs with a PEDro score  of ≥ 4.” | ^a^ |
| Wang et al., 2015 | Cochrane’s “Risk of bias” tool | **Random sequence generation:** 2 RCTs low risk  **Allocation concealment:** 2 RCTs low risk  **Blinding of participants and personnel:** 2 RCTs high risk  **Blinding of outcome assessment:** 2 RCTs low risk  **Incomplete outcome data:** 2 RCTs low risk  **Selective reporting:** 2 RCTs low risk;  **Other sources of bias:** 2 RCTs unclear risk | ^a^ | **Random sequence generation:** 100% RCTs low risk  **Allocation concealment:** 100% RCTs low risk  **Blinding of participants and personnel:** 100% RCTs high risk  **Blinding of outcome assessment:** 100% RCTs low risk  **Incomplete outcome data:** 100% RCTs low risk  **Selective reporting:** 100% RCTs low risk;  **Other sources of bias:** 100% RCTs unclear risk |
| Warnier et al., 2019 | Physiotherapy Evidence  Database (PEDro) Scale | **Random sequence generation:** 7 RCTs low risk  **Allocation concealment:** 4 RCTs low risk; 3 RCTs high  **Blinding of participants:** 7 RCTs high risk  **Blinding of personnel:** 7 RCTs high risk  **Blinding of outcome assessment:** 3 RCTs low risk; 4 RCTs high risk  **Incomplete outcome data:** 2 RCTs low risk; 5 RCTs high risk  **Group similarity at baseline:** 7 RCTs low risk  **Intention to treat:** 1 RCT low risk; 6 RCTs high risk | “Of these  studies, eight received a total of ≥6 points on the Pedro  scale which represents good quality. Two  other studies received five points. Two studies received  a total score of three and the remaining two studies scored  two points and one point, respectively.” | **Random sequence generation:** 100% RCTs low risk  **Allocation concealment:** 57% RCTs low risk; 43% RCTs high  **Blinding of participants:** 100% RCTs high risk  **Blinding of personnel:** 100% RCTs high risk  **Blinding of outcome assessment:** 43% RCTs low risk; 57% RCTs high risk  **Incomplete outcome data:** 29% RCTs low risk; 71% RCTs high risk  **Group similarity at baseline:** 100% RCTs low risk  **Intention to treat:** 14% RCT low risk; 86% RCTs high risk |
| Wattchow et al., 2018 | Cochrane’s “Risk of bias” tool | **Random sequence generation:** 1 RCT low risk; 3 RCTs unclear risk  **Allocation concealment:** 1 RCT low risk; 3 RCTs unclear risk  **Blinding of participants and personnel:** 1 RCT low risk; 3 RCTs high risk  **Blinding of outcome assessment:** 2 RCTs low risk; 2 RCTs high risk  **Incomplete outcome data:** 2 RCTs low risk; 2 RCTs high risk  **Selective reporting:** 3 RCTs low risk; 1 RCT unclear risk  **Other sources of bias:** 2 RCTs low risk; 2 RCTs unclear risk | ^a^ | **Random sequence generation:** 25% RCTs low risk; 75% RCTs unclear risk  **Allocation concealment:** 25% RCTs low risk; 75% RCTs unclear risk  **Blinding of participants and personnel:** 25% RCT low risk; 75% RCTs high risk  **Blinding of outcome assessment:** 50% RCTs low risk; 50% RCTs high risk  **Incomplete outcome data:** 50% RCTs low risk; 50% RCTs high risk  **Selective reporting:** 75% RCTs low risk; 25% RCTs unclear risk  **Other sources of bias:** 50% RCTs low risk; 50% RCTs unclear risk |
| Wiley et al., 2020 | Cochrane’s “Risk of bias” tool | **Random sequence generation:** 4 RCTs low risk; 1 RCTs high or unclear risk  **Allocation concealment:** 4 RCTs low risk; 1 RCTs high or unclear risk  **Blinding of participants and personnel:** 5 RCTs high or unclear risk  **Blinding of outcome assessment:** 1 RCTs low risk; 4 RCTs high or unclear risk  **Incomplete outcome data:** 5 RCT low risk  **Selective reporting:** 5 RCTs low risk |  | **Random sequence generation:** 80% RCTs low risk; 20% RCTs high or unclear risk  **Allocation concealment:** 80% RCTs low risk; 20% RCTs high or unclear risk  **Blinding of participants and personnel:** 100% RCTs high or unclear risk  **Blinding of outcome assessment:** 10% RCTs low risk; 90% RCTs high risk  **Incomplete outcome data:** 100% RCT low risk  **Selective reporting:** 100% RCTs low risk |
| Wu et al., 2019 | Physiotherapy Evidence  Database (PEDro) Scale | **Random sequence generation:** 11 RCTs low risk  **Allocation concealment:** 11 RCTs high risk  **Blinding of participants:** 11 RCTs high risk  **Blinding of personnel:** 11 RCTs high risk  **Blinding of outcome assessment:** 1 RCT low risk; 10 RCTs high risk  **Incomplete outcome data:** 11 RCTs low risk  **Group similarity at baseline:** 11 RCTs low risk  **Intention to treat:** 5 RCTs low risk; 5 RCTs high risk; 1 RCT at unclear risk | “Ten studies were deemed to have a  moderate risk of bias. Since allocation concealment was not conducted in all the included studies,  the quality of included studies decreased. Blinding was not frequently carried out in the studies (it was  only conducted in one study [31]), so the score of blinding was deducted for most of the studies.” | **Random sequence generation:** 100% RCTs low risk  **Allocation concealment:** 100% RCTs high risk  **Blinding of participants:** 100% RCTs high risk  **Blinding of personnel:** 100% RCTs high risk  **Blinding of outcome assessment:** 9% RCT low risk; 91% RCTs high risk  **Incomplete outcome data:** 100% RCTs low risk  **Group similarity at baseline:** 100% RCTs low risk  **Intention to treat:** 45% RCT low risk; 45% RCTs high risk; 10% RCT at unclear risk |

*Note.* ^a^ Data not available; n/a: not applicable; Random sequence generation (selection bias); Allocation concealment (selection bias); Blinding of participants and personnel (performance bias); Blinding of outcome assessment (detection bias); Incomplete outcome data (attrition bias); Selective reporting (reporting bias); Intention to treat (attrition bias)

Supplementary Table S4

*Details of included reviews. Reported effects of interventions and quality of evidence for reported outcomes: Lower limb activity*

| Author(s), year | Design, number of studies *(number of effects included in the analysis)*, total number of participants per outcome | Intervention, number of studies and participants per comparison | Control, number of studies and participants per comparison | Outcome and effect measure (95% CI) | Heterogeneity (I^2^) | Quality of the evidence (GRADE) | Reason for quality of evidence ratings |
| --- | --- | --- | --- | --- | --- | --- | --- |
| Barclay et al., 2015 | RCT, 1 *(1)*, 20 participants | VR-based interventions^a^, 1 (n = 11) | Conventional therapy, 1 (n = 9) | Time to walk: MD = -3.64 *ns* [-23.07 to 15.79] | n/a | Moderate | Imprecision (- 2): number of participants within pooled analysis less than 100; wide CI crossing line of no effect |
| Barclay et al., 2015 | RCT, 1 *(1)*, 20 participants | VR-based interventions^a^, 1 (n = 11) | Conventional therapy, 1 (n = 9) | Walking ability: MD = -0.31 *ns* [-10.31 to 9.69] | n/a | Moderate | Imprecision (- 2): number of participants within pooled analysis less than 100 |
| Barclay et al., 2015 | RCT, 1 *(1)*, 20 participants | VR-based interventions^a^, 1 (n = 11) | Conventional therapy, 1 (n = 9) | Gait speed: MD = 0.09 *ns* [-0.36 to 0.54] | n/a | Moderate | Imprecision (- 2): number of participants within pooled analysis less than 100 |
| Barclay et al., 2015 | RCT, 1 *(1)*, 20 participants | VR-based interventions^a^, 1 (n = 11) | Conventional therapy, 1 (n = 9) | Self-efficacy: SMD = 0.41 *ns* [-0.48 to 1.31] | n/a | Moderate | Imprecision (- 2): number of participants within pooled analysis less than 100 |
| Chen et al., 2018 | RCTs, 8 *(8)*, 282 participants | VR-based interventions^a^, 8 (n = 144) | Conventional therapy, 6 (n = 75); No intervention, 2 (n = 63) | Ambulation function: SMD = 0.75^ss^ [0.34 to 1.16] in favor of VR | 59.00 | Moderate | Risk of bias (review quality) (-2): no comprehensive literature search; status of publication (i.e., grey literature) was not used as an inclusion criteria |
| Cheok et al., 2015 | RCTs, 2 *(2)*, 42 participants | VR-based interventions with conventional therapy, 2 (n = 21) | Conventional therapy, 2 (n = 21) | Functional mobility: SMD = 0.64* [0.00 to 1.28] in favor of VR | 67.00 | Very low | Imprecision (- 2): number of participants within pooled analysis less than 100  Risk of bias (trial quality) (-1): ROB for individual trials could not be estimated as the trials were not fully described for this outcome, we were conservative and assumed that less than 75% of participants had low ROB  Risk of bias (review quality) (-2): no comprehensive literature search; status of publication (i.e., grey literature) was not used as an inclusion criteria |
| De Keersmmaecker et al., 2019 | Pre-post design, 7 *(7)*, 82 participants | VR-based interventions^a^  7 (n = 82) | n/a | Walking speed: MD = 0.17*** [0.12 to 0.22] in favor of post-test after VR | 0.00 | Low | Imprecision (-2): number of participants within pooled analysis less than 100  Risk of bias (review quality) (-2): no comprehensive literature search; status of publication (i.e., grey literature) was not used as an inclusion criteria |
| De Keersmmaecker et al., 2019 | Pre-post design, 4 *(4)*, 53 participants | VR-based interventions^a^  4 (n = 53) | n/a | Cadence: MD = 15.59** [7.26 to 23.91] in favor of post-test after VR | 29.00 | Low | Imprecision (-2): number of participants within pooled analysis less than 100  Risk of bias (review quality) (-2): no comprehensive literature search; status of publication (i.e., grey literature) was not used as an inclusion criteria |
| De Keersmmaecker et al., 2019 | Pre-post design, 4 *(4)*, 53 participants | VR-based interventions^a^  4 (n = 53) | n/a | Step length, paretic side: MD = 6.53** [3.69 to 9.37] in favor of post-test after VR | 00.00 | Low | Imprecision (-2): number of participants within pooled analysis less than 100  Risk of bias (review quality) (-2): no comprehensive literature search; status of publication (i.e., grey literature) was not used as an inclusion criteria |
| De Keersmmaecker et al., 2019 | Pre-post design, 4 *(4)*, 53 participants | VR-based interventions^a^  4 (n = 53) | n/a | Stride length, paretic side: MD = 12.27** [6.98 to 17.56] in favor of post-test after VR | 00.00 | Low | Imprecision (-2): number of participants within pooled analysis less than 100  Risk of bias (review quality) (-2): no comprehensive literature search; status of publication (i.e., grey literature) was not used as an inclusion criteria |
| De Keersmmaecker et al., 2019 | Pre-post design, 2 *(2)*, 22 participants | VR-based interventions^a^  2 (n = 22) | n/a | Single limb support period, paretic side: MD = 5.49** [2.87 to 8.12] in favor of post-test after VR | 00.00 | Low | Imprecision (-2): number of participants within pooled analysis less than 100  Risk of bias (review quality) (-2): no comprehensive literature search; status of publication (i.e., grey literature) was not used as an inclusion criteria |
| De Keersmmaecker et al., 2019 | Pre-post design, 4 *(4)*, 43 participants | VR-based interventions^a^  4 (n = 43) | n/a | Timed Up and Go Test: MD = 3.42** [1.71 to 5.12] in favor of post-test after VR | 29.00 | Low | Imprecision (-2): number of participants within pooled analysis less than 100  Risk of bias (review quality) (-2): no comprehensive literature search; status of publication (i.e., grey literature) was not used as an inclusion criteria |
| Domínguez-Téllez et al., 2019a | RCTs, 4 *(4)*, 128 participants | VR-based interventions with conventional therapy, 2 (n = 63) | Conventional therapy, 2 (n = 65) | Timed Up and Go: SMD = 1.42^ss^ [1.03 to 1.81] in favor of VR | ^d^ | Very low | Imprecision (-1): number of participants within pooled analysis less than 100  Inconsistency (-1): I^2^ not reported, assumed to be greater than 75%  Risk of bias (trial quality) (-1): ROB for individual biases could not be extracted, we were conservative and assumed that < 75% of participants had low ROB  Risk of bias (review quality) (-2): no comprehensive literature search; status of publication (i.e., grey literature) was not used as an inclusion criteria |
| Domínguez-Téllez et al., 2019a | RCTs, 4 *(4)*, 85 participants | VR-based interventions with conventional therapy, 4 (n = 44) | Conventional therapy, 4 (n = 41) | Gait (Ten-Minute Walk Test): SMD = -1.10 *ns* [-2.66 to 0.45] | ^d^ | Very low | Imprecision (-2): number of participants within pooled analysis less than 100  Inconsistency (-1): I^2^ not reported, assumed to be greater than 75%  Risk of bias (trial quality) (-1): ROB for individual biases could not be extracted, we were conservative and assumed that < 75% of participants had low ROB  Risk of bias (review quality) (-2): no comprehensive literature search; status of publication (i.e., grey literature) was not used as an inclusion criteria |
| Domínguez-Téllez et al., 2019a | RCTs, 3 *(3)*, 70 participants | VR-based interventions with conventional therapy, 3 (n = 35) | Conventional therapy, 3 (n = 35) | Cadence: SMD = -1.51^ss^ [-2.05 to -0.97] in favor of VR | ^d^ | Very low | Imprecision (-2): number of participants within pooled analysis less than 100  Inconsistency (-1): I^2^ not reported, assumed to be greater than 75%  Risk of bias (trial quality) (-1): ROB for individual biases could not be extracted, we were conservative and assumed that < 75% of participants had low ROB  Risk of bias (review quality) (-2): no comprehensive literature search; status of publication (i.e., grey literature) was not used as an inclusion criteria |
| Domínguez-Téllez et al., 2019a | RCTs, 3 *(3)*, 70 participants | VR-based interventions with conventional therapy, 3 (n = 35) | Conventional therapy, 3 (n = 35) | Step length: SMD = -1.63^ss^ [-2.18 to -1.08] in favor of VR | ^d^ | Very low | Imprecision (-2): number of participants within pooled analysis less than 100  Inconsistency (-1): I^2^ not reported, assumed to be greater than 75%  Risk of bias (trial quality) (-1): ROB for individual biases could not be extracted, we were conservative and assumed that < 75% of participants had low ROB  Risk of bias (review quality) (-2): no comprehensive literature search; status of publication (i.e., grey literature) was not used as an inclusion criteria |
| Domínguez-Téllez et al., 2019a | RCTs, 3 *(3)*, 70 participants | VR-based interventions with conventional therapy, 3 (n = 35) | Conventional therapy, 3 (n = 35) | Stride length: SMD = -1.63^ss^ [-2.18 to -1.08] in favor of VR | ^d^ | Very low | Imprecision (-2): number of participants within pooled analysis less than 100  Inconsistency (-1): I^2^ not reported, assumed to be greater than 75%  Risk of bias (trial quality) (-1): ROB for individual biases could not be extracted, we were conservative and assumed that < 75% of participants had low ROB  Risk of bias (review quality) (-2): no comprehensive literature search; status of publication (i.e., grey literature) was not used as an inclusion criteria |
| Domínguez-Téllez et al., 2019a | RCTs, 2 *(2)*, 46 participants | VR-based interventions with conventional therapy, 2 (n = 23) | Conventional therapy, 2 (n = 23) | Speed: SMD = -1.58^ss^ [-2.97 to -0.18] in favor of VR | ^d^ | Very low | Imprecision (-2): number of participants within pooled analysis less than 100  Inconsistency (-1): I^2^ not reported, assumed to be greater than 75%  Risk of bias (trial quality) (-1): ROB for individual biases could not be extracted, we were conservative and assumed that < 75% of participants had low ROB  Risk of bias (review quality) (-2): no comprehensive literature search; status of publication (i.e., grey literature) was not used as an inclusion criteria |
| Ferreira et al., 2020 | RCTs, 7 *(7)*, 168 participants | VR-based interventions with conventional therapy, 5 (n = 60); VR-based interventions alone, 2 (n = 22) | Conventional therapy, 6 (n = 73); No intervention, 1 (n = 13) | Mobility (Timed Up and Go): MD = -0.51 *ns* [-2.66, 1.64] | 0.00 | Low | Imprecision (-1): number of participants within pooled analysis less than 200  Risk of bias (review quality) (-2): no comprehensive literature search; status of publication (i.e., grey literature) was not used as an inclusion criteria |
| García-Muñoz et al, 2019 | RCTs, 6 *(6)*, 147 participants | VR-based interventions with conventional therapy, 5 (n = 60); VR-based interventions alone, 1 (n = 13) | Conventional therapy, 6 (n = 74) | Timed Up and Go: MD = -0.65 *ns* [-2.91 to 1.60] | 21.00 | Low | Imprecision (-1): number of participants within pooled analysis less than 200  Risk of bias (review quality) (-2): no comprehensive literature search; status of publication (i.e., grey literature) was not used as an inclusion criteria |
| Ghai et al., 2019 | Pre-post design, 12 *(13)*, 184 participants | VR-based interventions^a^, 12 (n = 184) | n/a | Gait velocity: Hedge’s *g* = 0.76^ss^ [0.44 to 1.07] in favor of post-test after VR | 10.70 | Low | Imprecision (- 1): number of participants within pooled analysis less than 200  Risk of bias (trial quality) (-1): 7 RCTs at high risk of detection bias (blinding of outcome assessment)  Risk of bias (review quality) (-2): no comprehensive literature search; status of publication (i.e., grey literature) was not used as an inclusion criteria; data extraction was not performed in duplicate |
| Gibbons et al., 2016 | RCTs, 6 *(6)*, 160 | VR-based interventions^a^, 6 (n = 79) | Conventional therapy, 6 (n = 81) | Functional mobility for chronic stroke: WMD = −2.04 *ns* [−5.82 to 1.75] | 58.00 | Low | Imprecision (- 1): number of participants within pooled analysis less than 200  Risk of bias (trial quality) (-1): 4 RCTs at high risk of selection bias (sequence generation); 1 RCTs at high risk of detection bias;  Risk of bias (review quality) (-2): no comprehensive literature search; status of publication (i.e., grey literature) was not used as an inclusion criteria |
| Gibbons et al., 2016 | RCTs, 7 *(7)*, 160 participants | VR-based interventions with conventional therapy, 1 (n = 10); VR-based interventions alone, 6 (n = 71) | Conventional therapy, 7 (n = 79) | Spatiotemporal characteristics/kinematics of gait, gait velocity for chronic stroke: WMD = 0.12^ss^ [0.03 to 0.22] in favor of VR | 0.00 | Low | Imprecision (-1): number of participants within pooled analysis between 100 – 199  Risk of bias (review quality) (-2): no comprehensive literature search; status of publication (i.e., grey literature) was not used as an inclusion criteria |
| Gibbons et al., 2016 | RCTs, 3 *(3)*, 60 participants | VR-based interventions alone, 3 (n = 30) | Conventional therapy, 3 (n = 30) | Spatiotemporal characteristics/kinematics of gait for chronic stroke, stride length: WMD = 9.79* [0.74 to 18.84] in favor of VR | 0.00 | Low | Imprecision (- 2): number of participants within pooled analysis less than 100  Risk of bias (review quality) (-2): no comprehensive literature search; status of publication (i.e., grey literature) was not used as an inclusion criteria |
| Gibbons et al., 2016 | RCTs, 3 *(3)*, 60 participants | VR-based interventions alone, 3 (n = 30) | Conventional therapy, 3 (n = 30) | Spatiotemporal characteristics/kinematics of gait for chronic stroke, step length: WMD = 5.74* [0.91 to 10.56] in favor of VR | 0.00 | Low | Imprecision (- 2): number of participants within pooled analysis less than  Risk of bias (review quality) (-2): no comprehensive literature search; status of publication (i.e., grey literature) was not used as an inclusion criteria |
| Gibbons et al., 2016 | RCTs, 2 *(2)*, 44 | VR-based interventions alone, 2 (n = 22) | Conventional therapy, 2 (n = 22) | Spatiotemporal characteristics/kinematics of gait for chronic stroke, stance time: SMD = -0.58 *ns* [-1.72 to 0.56] | 70.00 | Low | Imprecision (- 2): number of participants within pooled analysis less than 100  Risk of bias (review quality) (-2): no comprehensive literature search; status of publication (i.e., grey literature) was not used as an inclusion criteria |
| Iruthayarajah et al., 2017 | RCT, 13 *(13)*, 292 participants | VR-based interventions with conventional therapy, 5 (n = 52); VR-based interventions alone, 8 (n = 93) | Conventional therapy, 12 (n = 137); No intervention, 1 (n = 10) | Functional mobility (Timed Up and Go Test): SMD = 0.36** [0.13 to 0.60] in favor of VR | 0.00 | Moderate | Risk of bias (review quality) (-2): no comprehensive literature search; status of publication (i.e., grey literature) was not used as an inclusion criteria |
| Lee et al., 2019 | RCT, 12 *(74)*, 310 participants | VR-based interventions with conventional therapy, 10 (n = 118); VR-based interventions alone, 2 (n = 40) | Conventional therapy, 12 (n = 152) | Lower limb function: Hedge’s *g* = 0.42*** [0.33 to 0.51] in favor of VR | ^d^ | Low | Risk of bias (review quality) (-2): no comprehensive literature search; status of publication (i.e., grey literature) was not used as an inclusion criteria; data extraction was not performed in duplicate  Inconsistency (-1): I^2^ not reported, assumed to be greater than 75%  Risk of bias (trial quality) (-1): 6 RCTs at high risk of selection bias (allocation concealment); 5 RCTs at high risk for detection bias (blinding of outcome assessment) |
| Li et al., 2016 | RCTs, 8 *(8)*, 214 participants | VR-based interventions with conventional therapy, 6 (n = 88); VR-based interventions alone, 2 (n = 17) | Conventional therapy, 8 (n = 109) | Timed Up and Go Test: MD = –1.62*[–3.07 to –0.16] in favor of VR | 24.00 | Moderate | Risk of bias (review quality) (-2): no comprehensive literature search; status of publication (i.e., grey literature) was not used as an inclusion criteria |
| Lin et al., 2018 | RCTs, 2 *(2)*, 80 participants | VR-based interventions with conventional therapy, 2 (n = 41) | No intervention, 2 (n = 39) | Lower extremity: SMD = 0.60** [0.15 to 1.05] in favor of VR | 0.00 | Very low | Imprecision (- 2): number of participants within pooled analysis less than 100  Risk of bias (trial quality) (-1): ROB for individual trials could not be estimated as the trials were not fully described for this outcome, we were conservative and assumed that less than 75% of participants had low ROB  Risk of bias (review quality) (-2): no comprehensive literature search; status of publication (i.e., grey literature) was not used as an inclusion criteria |
| Rodrigues-Baroni et al., 2014 | RCTs, 5 *(5)*, 92 participants | VR-based interventions with conventional therapy, 2^b^; VR-based interventions alone, 3^b^ | Conventional therapy, 5^b^ | Walking speed: MD = 0.15^ss^ [0.05 to 0.24] in favor of VR | 0.00 | Low | Imprecision (- 2): number of participants within pooled analysis less than 100  Risk of bias (review quality) (-2): no comprehensive literature search; status of publication (i.e., grey literature) was not used as an inclusion criteria; study selection and data extraction was not performed in duplicate |
| Rodrigues-Baroni et al., 2014 | RCTs, 3 *(3)*, 72 participants | VR-based interventions with conventional therapy, 2^b^; VR-based interventions alone, 1^b^ | No intervention, 3^b^ | Walking speed: MD = 0.17^ss^ [0.08 to 0.26] in favor of VR | 0.00 | Low | Imprecision (- 2): number of participants within pooled analysis less than 100  Risk of bias (review quality) (-2): no comprehensive literature search; status of publication (i.e., grey literature) was not used as an inclusion criteria; study selection and data extraction was not performed in duplicate |
| Rutkowski et al., 2020 | RCTs, 3 *(5)*, 106 participants | VR-based interventions with conventional therapy, 1 (n = 13); VR-based interventions alone, 2 (n = 41) | Conventional therapy, 3 (n = 52) | Lower limb function (gait): SMD = -0.36 *ns* [-0.74 to 0.03] | 0.00 | Very low | Imprecision (- 1): number of participants within pooled analysis less than 200  Risk of bias (review quality) (-2): no comprehensive literature search; status of publication (i.e., grey literature) was not used as an inclusion criteria |
| Tay et al., 2018 | RCTs, 4 *(4)*, 119 participants | VR-based interventions with conventional therapy, 4 (n = 61) | Conventional therapy, 4 (n = 58) | Mobility (Timed Up and Go Test): WMD = −4.48** [−5.43 to −3.52] in favor of VR | 0.00 | Low | Imprecision (- 1): number of participants within pooled analysis less than 200  Risk of bias (trial quality) (-1): ROB for individual trials could not be estimated as the trials were not fully described for this outcome, we were conservative and assumed that less than 75% of participants had low ROB  Risk of bias (review quality) (-2): no comprehensive literature search; status of publication (i.e., grey literature) was not used as an inclusion criteria |
| Tay et al., 2018 | RCTs, 3 *(3)*, 92 participants | VR-based interventions with conventional therapy, 2 (n = 39); VR-based interventions^a^, 1 (n = 8) | Conventional therapy, 3 (n = 45) | Walk tests: SMD = 0.47* [ 0.05 to 0.89] in favor of VR | 0.00 | Very low | Imprecision (- 2): number of participants within pooled analysis less than 100  Risk of bias (trial quality) (-1): ROB for individual trials could not be estimated as the trials were not fully described for this outcome, we were conservative and assumed that less than 75% of participants had low ROB  Risk of bias (review quality) (-2): no comprehensive literature search; status of publication (i.e., grey literature) was not used as an inclusion criteria |
| Saywell et al., 2017 | RCTs, 10 *(10)*, 260 participants | VR-based interventions with conventional therapy, 5 (n = 91); VR-based interventions alone, 5 (n = 49) | Conventional therapy, 8 (n = 109); No intervention, 2 (n = 19) | Lower limb gait: SMD = 0.31 *ns* [0.07 to 0.55] | 0.00 | Low | Risk of bias (review quality) (-2): no comprehensive literature search; status of publication (i.e., grey literature) was not used as an inclusion criteria |
| Veerbeek et al., 2014 | RCTs, 2 *(2)*, 42 participants | VR-based interventions^a^, 2^b^ | ^b,c^ | Comfortable gait speed: Hedge’s *g* = -0.46 *ns* [-1.34 to 0.41] | 54.00 | Very low | Imprecision (- 2): number of participants within pooled analysis less than 100  Risk of bias (trial quality) (-1): ROB for individual trials could not be estimated as the trials were not fully described for this outcome, we were conservative and assumed that less than 75% of participants had low ROB  Risk of bias (review quality) (-2): status of publication (i.e., grey literature) was not used as an inclusion criteria; study selection and data extraction was not performed in duplicate |
| Veerbeek et al., 2014 | RCTs^c^, 58 participants | VR-based interventions ^a ,b,c^ | ^b,c^ | Maximum gait speed: Hedge’s *g* = 0.21 *ns* [-0.28 to 0.71] | 0.00 | Very low | Imprecision (- 2): number of participants within pooled analysis less than 100  Risk of bias (trial quality) (-1): ROB for individual trials could not be estimated as the trials were not fully described for this outcome, we were conservative and assumed that < 75% of participants had low ROB  Risk of bias (review quality) (-2): status of publication (i.e., grey literature) was not used as an inclusion criteria; study selection and data extraction was not performed in duplicate |
| Veerbeek et al., 2014 | RCTs^c^ 42 participants | VR-based interventions ^a,b^ | ^b,c^ | Step length: Hedge’s *g* = 0.18 *ns* [-0.40 to 0.77] | 31.00 | Very low | Imprecision (- 2): number of participants within pooled analysis less than 100  Risk of bias (trial quality) (-1): ROB for individual trials could not be estimated as the trials were not fully described for this outcome, we were conservative and assumed that less than 75% of participants had low ROB  Risk of bias (review quality) (-2): status of publication (i.e., grey literature) was not used as an inclusion criteria; study selection and data extraction was not performed in duplicate |
| Veerbeek et al., 2014 | RCTs, 2 *(2)*, 30 participants | VR-based interventions ^a,b^ | ^b,c^ | Walking ability: Hedge’s *g* = 0.16 *ns* [-0.51 to 0.84] | 0.00 | Very low | Imprecision (- 2): number of participants within pooled analysis less than 100  Risk of bias (trial quality) (-1): ROB for individual trials could not be estimated as the trials were not fully described for this outcome, we were conservative and assumed that < 75% of participants had low ROB  Risk of bias (review quality) (-2): status of publication (i.e., grey literature) was not used as an inclusion criteria; study selection and data extraction was not performed in duplicate |
| Warnier et al., 2019 | RCTs, 4 *(5)*, 108 participants | VR-based interventions alone, 4 (n = 54) | Control group^c^, 4 (n = 54) | Walking: SMD = 3.10** [0.78 to 5.43] in favor of VR | 92.00 | Low | Imprecision (- 1): number of participants within pooled analysis less than 200  Risk of bias (trial quality) (-1): 3 RCTs at high risk of selection bias (allocation concealment); 7 RCTs at high risk of performance bias; 4 RCTs at high risk of detection bias; 6 RCTs at high risk of attrition bias  Risk of bias (review quality) (-2): no comprehensive literature search; status of publication (i.e., grey literature) was not used as an inclusion criteria |

*Note.* *** *p* < .001, ** *p* < .01, * *p* < .05, *ns*- non-significant results. ^a^ = we were not able to identify whether VR-based interventions were delivered alone or in combination with conventional therapy; ^b^ = number of participants in the intervention/control group could not be extracted; ^c^ = control group type not specified; ^d^ = I^2^ value not reported; Hedge’s *g* = Hedge’s *g* coefficient, effect size; n = sample size; n/a = not applicable, pre-test post-test design; MD = Mean Difference, effect size; RCT = randomized controlled trial; SMD = Standardized Mean Difference, effect size; ^ss^ = statistically significant results, without *p* value provided by authors; WMD = Weighted mean difference, effect size

Supplementary Table S5

*Details of included reviews. Reported effects of interventions and quality of evidence for reported outcomes: Balance and postural control*

| Author(s), year | Design, number of studies *(number of effects included in the analysis)*, total number of participants per outcome | Intervention | Control or comparison intervention | Effect measure (95% CI) | Heterogeneity (I^2^) | Quality of the evidence | Reason for quality of evidence ratings |
| --- | --- | --- | --- | --- | --- | --- | --- |
| Booth et al., 2014 | RCT, 1 *(1)*, 17 participants | VR-based interventions alone, 1 (n = 9) | Conventional therapy, 1 (n = 8) | Balance (Sit to Stand Test): MD = 0.50 *ns* [-3.28 to 4.28] | n/a | Low | Imprecision (- 2): number of participants within pooled analysis less than 100; wide CI crossing line of no effect  Risk of bias (review quality) (-1): status of publication (i.e., grey literature) was not used as an inclusion criteria |
| Chen et al., 2015 | RCTs, 2 *(2)*, 54 participants | VR-based interventions^a^, 2 (n = 27) | Conventional therapy, 2 (n = 27) | Balance (Berg Balance Scale): SMD: -0.17*ns* [-0.70 to 0.37] | 0.00 | Low | Imprecision (- 2): number of participants within pooled analysis less than 100  Risk of bias (review quality) (-2): no comprehensive literature search; status of publication (i.e., grey literature) was not used as an inclusion criteria |
| Chen et al., 2018 | RCT, 10 *(10)*, 227 participants | VR-based interventions alone, 10 (n = 114) | Conventional therapy, 8 (n = 86); No intervention, 2 (n = 27) | Postural Control: SMD = 1.00^ss^ [0.503 to 1.502] in favor of VR | 67.00 | Low | Risk of bias (trial quality) (-1): ROB for individual biases was not reported for this outcome, total score of risk of bias indicates that < 75% of participants had low ROB  Risk of bias (review quality) (-2): no comprehensive literature search; status of publication (i.e., grey literature) was not used as an inclusion criteria |
| Cheok et al., 2015 | RCTs, 2 *(2)*, 42 participants | VR-based interventions with conventional therapy, 2 (n = 21) | Conventional therapy, 2 (n = 21) | Balance (Berg Balance Scale): SMD = 0.39 *ns* [-0.25 to 1.04] | 85.00 | Very low | Imprecision (- 2): number of participants within pooled analysis less than 100  Inconsistency (-1): I^2^ not reported, assumed to be greater than 75%  Risk of bias (trial quality) (-1): ROB for individual trials could not be estimated as the trials were not fully described for this outcome, we were conservative and assumed that < 75% of participants had low ROB  Risk of bias (review quality) (-2): no comprehensive literature search; status of publication (i.e., grey literature) was not used as an inclusion criteria |
| Cheok et al., 2015 | RCTs, 2 *(2)*, 42 participants | VR-based interventions with conventional therapy, 2 (n = 21) | Conventional therapy, 2 (n = 21) | Static balance (Postural sway measures, anteroposterior eyes open): SMD = 0.21 *ns* [-0.40 to 0.82] | 0.00 | Very low | Imprecision (- 2): number of participants within pooled analysis less than 100  Risk of bias (trial quality) (-1): ROB for individual trials could not be estimated as the trials were not fully described for this outcome, we were conservative and assumed that < 75% of participants had low ROB  Risk of bias (review quality) (-2): no comprehensive literature search; status of publication (i.e., grey literature) was not used as an inclusion criteria |
| Cheok et al., 2015 | RCTs, 2 *(2)*, 42 participants | VR-based interventions with conventional therapy, 2 (n = 21) | Conventional therapy, 2 (n = 21) | Static balance (Postural sway measures, anteroposterior eyes closed): SMD = 0.18 *ns* [-0.43 to 0.79] | 0.00 | Very low | Imprecision (- 2): number of participants within pooled analysis less than 100  Risk of bias (trial quality) (-1): ROB for individual trials could not be estimated as the trials were not fully described for this outcome, we were conservative and assumed that < 75% of participants had low ROB  Risk of bias (review quality) (-2): no comprehensive literature search; status of publication (i.e., grey literature) was not used as an inclusion criteria |
| Cheok et al., 2015 | RCTs, 2 *(2)*, 42 participants | VR-based interventions with conventional therapy, 2 (n = 21) | Conventional therapy, 2 (n = 21) | Static balance (Postural sway measures, medio-lateral eyes open): SMD = 0.29 *ns* [-0.43 to 0.79] | 0.00 | Very low | Imprecision (- 2): number of participants within pooled analysis less than 100  Risk of bias (trial quality) (-1): ROB for individual trials could not be estimated as the trials were not fully described for this outcome, we were conservative and assumed that < 75% of participants had low ROB  Risk of bias (review quality) (-2): no comprehensive literature search; status of publication (i.e., grey literature) was not used as an inclusion criteria |
| Cheok et al., 2015 | RCTs, 2 *(2)*, 42 participants | VR-based interventions with conventional therapy, 2 (n = 21) | Conventional therapy, 2 (n = 21) | Static balance (Postural sway measures, medio-lateral eyes closed): SMD = 0.16 *ns* [-0.45 to 0.77] | 0.00 | Very low | Imprecision (- 2): number of participants within pooled analysis less than 100  Risk of bias (trial quality) (-1): ROB for individual trials could not be estimated as the trials were not fully described for this outcome, we were conservative and assumed that < 75% of participants had low ROB  Risk of bias (review quality) (-2): no comprehensive literature search; status of publication (i.e., grey literature) was not used as an inclusion criteria |
| Corbetta et al., 2015 | RCTs, 5 *(5)*, 130 participants | VR-based interventions alone, 5 (n = 67) | Conventional therapy, 5 (n = 63) | Balance (Berg Balance Scale): MD = 2.13** [1.76 to 2.51] in favor of VR | 0.00 | Low | Imprecision (- 1): number of participants within pooled analysis less than 200  Risk of bias (review quality) (-2): no comprehensive literature search; status of publication (i.e., grey literature) was not used as an inclusion criteria |
| De Keersmmaecker et al., 2019 | Pre-post design, 2 *(2)*, 22 participants | VR-based interventions^a^2 (n = 22) | n/a | Berg Balance Scale: MD = 3.83** [2.19 to 5.46] in favor of post-test after VR | 00.00 | Low | Imprecision (-2): number of participants within pooled analysis less than 100  Risk of bias (review quality) (-2): no comprehensive literature search; status of publication (i.e., grey literature) was not used as an inclusion criteria |
| Domínguez-Téllez et al., 2019a | RCTs, 5 *(5)*, 129 participants | VR-based interventions with conventional therapy, 5 (n = 65) | Conventional therapy, 5 (n = 64) | Berg Balance Scale: SMD = -1.89^ss^ [-2.72 to -1.07] in favor of VR | ^c^ | Very low | Imprecision (-1): number of participants within pooled analysis less than 200  Inconsistency (-1): I^2^ not reported, assumed to be greater than 75%  Risk of bias (trial quality) (-1): ROB for individual biases could not be extracted, we were conservative and assumed that < 75% of participants had low ROB  Risk of bias (review quality) (-2): no comprehensive literature search; status of publication (i.e., grey literature) was not used as an inclusion criteria |
| Domínguez-Téllez et al., 2019a | RCTs, 2 *(2)*, 49 participants | VR-based interventions with conventional therapy, 2 (n = 25) | Conventional therapy, 2 (n = 24) | Functional Reach Test: SMD = -1.21 *ns* [-2.84 to 0.40] | ^c^ | Very low | Imprecision (-2): number of participants within pooled analysis less than 100  Inconsistency (-1): I^2^ not reported, assumed to be greater than 75%  Risk of bias (trial quality) (-1): ROB for individual biases could not be extracted, we were conservative and assumed that < 75% of participants had low ROB  Risk of bias (review quality) (-2): no comprehensive literature search; status of publication (i.e., grey literature) was not used as an inclusion criteria |
| Ferreira et al., 2020 | RCTs, 6 *(6)*, 128 participants | VR-based interventions with conventional therapy, 4 (n = 43); VR-based interventions alone, 2 (n = 22) | Conventional therapy, 5 (n = 50); No intervention, 1 (n = 13) | Balance (Berg Balance Scale): MD = 2.24* [0.45 to 4.04] in favor of VR | 24.00 | Low | Imprecision (- 1): number of participants within pooled analysis less than 200  Risk of bias (review quality) (-2): no comprehensive literature search; status of publication (i.e., grey literature) was not used as an inclusion criteria |
| García-Muñoz et al, 2019 | RCTs, 3 *(5)*, 110 participants | VR-based interventions with conventional therapy, 2 (n = 20); VR-based interventions alone, 3 (n = 36) | Conventional therapy, 5 (n = 54) | Anteroposterior deviations of the center of gravity (eyes open and closed): MD = 0.04 *ns* [-0.03 to 0.12] | 6.00 | Low | Imprecision (-1): number of participants within pooled analysis less than 200  Risk of bias (review quality) (-2): no comprehensive literature search; status of publication (i.e., grey literature) was not used as an inclusion criteria |
| García-Muñoz et al, 2019 | RCTs, 3 *(5)*, 110 participants | VR-based interventions with conventional therapy, 2 (n = 20); VR-based interventions alone, 3 (n = 36) | Conventional therapy, 5 (n = 54) | Mediolateral deviations of the center of gravity (eyes open and closed): MD = -0.25 *ns* [-0.59 to 0.08] | 0.00 | Low | Imprecision (-1): number of participants within pooled analysis less than 200  Risk of bias (review quality) (-2): no comprehensive literature search; status of publication (i.e., grey literature) was not used as an inclusion criteria |
| García-Muñoz et al, 2019 | RCTs, 4 *(4)*, 95 participants | VR-based interventions with conventional therapy, 4 (n = 47) | Conventional therapy, 4 (n = 48) | Berg Balance Scale: MD = -0.43 *ns* [-5.06 to 4.20] | 77.00 | Very low | Imprecision (-2): number of participants within pooled analysis less than 100  Inconsistency (-1): I^2^ greater than 75%  Risk of bias (review quality) (-2): no comprehensive literature search; status of publication (i.e., grey literature) was not used as an inclusion criteria |
| García-Muñoz et al, 2019 | RCTs, 4 *(4)*, 105 participants | VR-based interventions with conventional therapy, 1 (n = 15); VR-based interventions alone, 3 (n = 37) | Conventional therapy, 4 (n = 53) | Functional Reach Test: MD = 1.12 *ns* [-0.89 to 3.14] | 00.00 | Low | Imprecision (-1): number of participants within pooled analysis less than 200  Risk of bias (review quality) (-2): no comprehensive literature search; status of publication (i.e., grey literature) was not used as an inclusion criteria |
| Gibbons et al., 2016 | RCTs, 9 *(9)*, 185 participants | VR-based interventions with conventional therapy, 9 (n =93) | Conventional therapy, 9 (n = 92) | Functional balance for chronic stroke: SMD = 0.42^ss^ [0.11 to 0.73] in favor of VR | 10.00 | Low | Imprecision (- 1): number of participants within pooled analysis less than 200  Risk of bias (trial quality) (-1): 6 RCTs at high risk of detection bias (blinding o outcome assessment)  Risk of bias (review quality) (-2): no comprehensive literature search; status of publication (i.e., grey literature) was not used as an inclusion criteria |
| Gibbons et al., 2016 | RCTs, 5 *(5)*, 113 participants | VR-based interventions with conventional therapy, 5 (n = 57) | Conventional therapy, 5, (n = 56) | Static balance for chronic stroke, postural sway measures, centre of pressure sway/path-length eyes open: SMD = −0.02 *ns* [−0.39 to 0.35] | 0.00 | Low | Imprecision (-1): number of participants within pooled analysis less  Risk of bias (review quality) (-2): no comprehensive literature search; status of publication (i.e., grey literature) was not used as an inclusion criteria |
| Gibbons et al., 2016 | RCTs, 3 *(3)*, 88 participants | VR-based interventions with conventional therapy, 3 (n = 46) | Conventional therapy, 3 (n = 42) | Functional balance for acute-subacute stroke: SMD = 0.42 *ns* [−0.21 to 1.06] | 50.00 | Low | Imprecision (- 2): number of participants within pooled analysis less than 100  Risk of bias (review quality) (-2): no comprehensive literature search; status of publication (i.e., grey literature) was not used as an inclusion criteria |
| Gibbons et al., 2016 | RCTs, 3 *(3)*, 65 participants | VR-based interventions with conventional therapy, 3 (n = 34) | Conventional therapy, 3 (n = 31) | Static balance for chronic stroke, postural sway measures, centre of pressure velocity: SMD = −0.10 *ns* [−0.59 to 0.39] | 0.00 | Low | Imprecision (- 2): number of participants within pooled analysis less than 100  Risk of bias (review quality) (-2): no comprehensive literature search; status of publication (i.e., grey literature) was not used as an inclusion criteria |
| Gibbons et al., 2016 | RCTs, 3 *(3)*, 75 participants | VR-based interventions with conventional therapy, 3 (n = 35) | Conventional therapy, 3 (n = 37) | Static balance for chronic stroke, postural sway measures, centre of pressure sway/path-length eyes closed: SMD = 0.16 *ns* [−0.31 to 0.6] | 0.00 | Low | Imprecision (- 2): number of participants within pooled analysis less than 100  Risk of bias (review quality) (-2): no comprehensive literature search; status of publication (i.e., grey literature) was not used as an inclusion criteria |
| Gibbons et al., 2016 | RCTs, 4 *(4)*, 122 participants | VR-based interventions with conventional therapy, 4 (n = 60) | Conventional therapy, 4 (n = 62) | Static balance for chronic stroke, postural sway measures, percentage weight bearing on affected limb: WMD = 0.28 *ns* [−1.93 to 2.49] | 37.00 | Low | Imprecision (- 1): number of participants within pooled analysis less than 200  Risk of bias (review quality) (-2): no comprehensive literature search; status of publication (i.e., grey literature) was not used as an inclusion criteria |
| Iruthayarajah et al., 2017 | RCT, 12 *(12)*, 265 participants | VR-based interventions with conventional therapy, 7 (n =74); VR-based interventions alone, 5 (n = 59) | Conventional therapy, 11 (n = 119); No intervention, 1 (n = 14) | Balance (Berg Balance Scale): SMD = 0.50*** [0.25 to 0.75] in favor of VR | ^c^ | Moderate | Risk of bias (review quality) (-2): no comprehensive literature search; status of publication (i.e., grey literature) was not used as an inclusion criteria |
| Li et al., 2016 | RCTs, 8 *(8)*, 170 participants | VR-based interventions with conventional therapy, 8 (n = 83) | Conventional therapy, 8 (n = 87) | Balance (Berg Balance Scale): MD = 1.46* [0.09 to 2.83] in favor of VR | 0.00 | Low | Imprecision (- 1): number of participants within pooled analysis less than 200  Risk of bias (trial quality) (-1): ROB for individual biases was not reported for this outcome, total score of risk of bias indicates that < 75% of participants had low ROB  Risk of bias (review quality) (-2): no comprehensive literature search; status of publication (i.e., grey literature) was not used as an inclusion criteria |
| Li et al., 2016 | RCTs, 2 *(2)*, 47 participants | VR-based interventions with conventional therapy, 2 (n = 23) | Conventional therapy, 2 (n = 24) | Balance (Functional Reach Test): MD = 1.97 *ns* [-0.22 to 4.17] | 60.00 | Low | Imprecision (- 2): number of participants within pooled analysis less than 100  Risk of bias (review quality) (-2): no comprehensive literature search; status of publication (i.e., grey literature) was not used as an inclusion criteria |
| Li et al., 2016 | RCTs, 2 *(2)*, 41 participants | VR-based interventions with conventional therapy, 1 (n = 11); VR-based interventions alone, 1 (n = 11) | Conventional therapy, 2 (n = 19) | Balance (Activities specific Balance Confidence Scale): MD = 3.73 *ns* [-1.01 to 8.46] | 77.00 | Very low | Imprecision (- 2): number of participants within pooled analysis less than 100  Inconsistency (-1): I^2^ greater than 75%  Risk of bias (review quality) (-2): no comprehensive literature search; status of publication (i.e., grey literature) was not used as an inclusion criteria |
| Li et al., 2016 | RCTs, 3 *(3)*, 69 participants | VR-based interventions with conventional therapy, 3 (n = 36) | Conventional therapy, 3 (n = 33) | Sway velocity: MD = 0.07 *ns* [-0.30 to 0.43] | 0.00 | Low | Imprecision (- 2): number of participants within pooled analysis less than 100  Risk of bias (review quality) (-2): no comprehensive literature search; status of publication (i.e., grey literature) was not used as an inclusion criteria |
| Li et al., 2016 | RCTs, 3 *(3)*, 74 participants | VR-based interventions with conventional therapy, 3 (n = 37) | Conventional therapy, 3 (n = 37) | Weight distribution: MD = -1.21 *ns* [-2.54 to 0.12] | 0.00 | Low | Imprecision (- 2): number of participants within pooled analysis less than 100  Risk of bias (review quality) (-2): no comprehensive literature search; status of publication (i.e., grey literature) was not used as an inclusion criteria |
| Mohammadi et al., 2019 | RCTs, 13 *(13)*, 325 participants | VR-based interventions with conventional therapy, 12 (n = 156); VR-based interventions alone, 1 (n = 10) | Conventional therapy, 13 (n = 159) | Balance (Berg Balance Scale): SMD = 0.64^ss^ [0.36 to 0.92] in favor of VR | 36.70 | Moderate | Risk of bias (review quality) (-2): no comprehensive literature search; status of publication (i.e., grey literature) was not used as an inclusion criteria; data extraction was not performed in duplicate |
| Prosperini et al., 2020 | RCTs, 18 *(18)*, 480 participants | VR-based interventions alone, 18 (n = 245) | Conventional therapy, 18 (n = 235) | Balance: Hedge’s *g* = 0.26^*^ [0.02 to 0.51] in favor of VR for people with stroke | ^c^ | Low | Inconsistency (-1): I^2^ not reported, assumed to be greater than 75%  Risk of bias (trial quality) (-1): ROB for individual biases could not be extracted, we were conservative and assumed that < 75% of participants had low ROB |
| Prosperini et al., 2020 | RCTs, 2 *(18)*, 41 participants | VR-based interventions alone, 2 (n = 21) | Conventional therapy, 2 (n = 20) | Balance: Hedge’s *g* = 0.05 *ns* [-0.61 to 0.62] for people with TBI | ^c^ | Very low | Imprecision (-2): number of participants within pooled analysis less than 100  Inconsistency (-1): I^2^ not reported, assumed to be greater than 75%  Risk of bias (trial quality) (-1): ROB for individual biases could not be extracted, we were conservative and assumed that < 75% of participants had low ROB |
| Saywell et al., 2017 | RCTs, 3 *(3)*, 67 participants | VR-based interventions with conventional therapy, dose matched, 3 (n = 34) | Conventional therapy, 3 (n = 33) | Lower limb balance: SMD = 0.14 *ns* [-0.34 to 0.62] | 0.00 | Very low | Imprecision (- 2): number of participants within pooled analysis less than 100  Risk of bias (trial quality) (-1): 3 RCTs at high risk of selection bias (sequence generation)  Risk of bias (review quality) (-2): no comprehensive literature search; status of publication (i.e., grey literature) was not used as an inclusion criteria |
| Tay et al., 2018 | RCTs, 3 *(3)*, 61 participants | VR-based interventions with conventional therapy, 1 (n = 12); VR-based interventions alone, 2 (n = 19) | Conventional therapy, 3 (n = 30) | Balance (Berg Balance Scale): WMD = 3.31* [0.79 to 5.82] in favour of VR | 27.00 | Very low | Imprecision (- 2): number of participants within pooled analysis less than 100  Risk of bias (trial quality) (-1): ROB for individual biases could not be extracted, we were conservative and assumed that < 75% of participants had low ROB  Risk of bias (review quality) (-2): no comprehensive literature search; status of publication (i.e., grey literature) was not used as an inclusion criteria |
| Wang et al., 2015 | RCTs, 2 *(2)*, 41 participants | VR-based interventions alone, 2 (n = 22) | Conventional therapy, 2 (n = 19) | Balance (Balance Confidence Scale): MD = 7.27 *ns* [-5.95 to 20.48] | 77.00 | Low | Imprecision (- 2): number of participants within pooled analysis less than 100  Inconsistency (-1): I^2^ greater than 75%  Risk of bias (review quality) (-1): status of publication (i.e., grey literature) was not used as an inclusion criteria |
| Warnier et al., 2019 | RCTs, 5 *(8)*, 140 participants | VR-based interventions alone, 5 (n = 69) | Control group^b^, 5 (n = 71) | Balance: SMD = 0.89* [0.14 to 1.63] in favor of VR | 70.00 | Low | Imprecision (- 1): number of participants within pooled analysis less than 200  Risk of bias (trial quality) (-1): 3 RCTs at high risk of selection bias (allocation concealment); 7 RCTs at high risk of performance bias; 4 RCTs at high risk of detection bias; 6 RCTs at high risk of attrition bias  Risk of bias (review quality) (-2): no comprehensive literature search; status of publication (i.e., grey literature) was not used as an inclusion criteria |
| Wu et al., 2019 | RCTs, 11 *(14)*, 448 participants | VR-based interventions with conventional therapy, 10 (n = 206); VR-based interventions alone, 1 (n = 20) | Control group^b^, 11 (n = 222) | Balance: SMD = 0.29** [0.10 to 0.48] in favor of VR | 0.00 | Low | Risk of bias (trial quality) (-1):11 RCTs at high risk of selection bias (allocation concealment); 11 RCTs at high risk of performance bias; 10 RCTs at high risk of detection bias; 6 RCTs at high risk of attrition bias  Risk of bias (review quality) (-2): no comprehensive literature search; status of publication (i.e., grey literature) was not used as an inclusion criteria |

*Note.* *** *p* < .001, ** *p* < .01, * *p* < .05, *ns*- non-significant results. ^a^ = we were not able to identify whether VR-based interventions were delivered alone or in combination with conventional therapy; ^b^ = control group type not specified; ^c^ = I^2^ value not reported; n = sample size; MD = Mean Difference, effect size; RCT = randomized controlled trial; SMD = Standardized Mean Difference, effect size; ^ss^ = statistically significant results, without *p* value provided by authors; WMD = Weighted mean difference, effect size

Supplementary Table S6

*Details of included reviews. Reported effects of interventions and quality of evidence for reported outcomes: Upper limb, arm function and activity*

| Author(s), year | Design, number of studies *(number of effects included in the analysis)*, total number of participants per outcome | Intervention | Control or comparison intervention | Effect measure (95% CI) | Heterogeneity (I^2^) | Quality of the evidence | Reason for quality of evidence ratings |
| --- | --- | --- | --- | --- | --- | --- | --- |
| Chen et al., 2014 | Pre- and post VR design, 14 *(14)*, 97 participants | VR-based interventions^a^, 14 (n = 97) | n/a | Upper extremity: SMD = 0.96*** [0.45 to 1.56] in favor of post-test after VR | 56.00 | Low | Imprecision (- 2): number of participants within pooled analysis less than 100  Risk of bias (review quality) (-2): study selection and data extraction was not performed in duplicate; no comprehensive literature search; status of publication (i.e., grey literature) was not used as an inclusion criteria |
| Chen et al., 2014 | RCTs, 3 *(3)*, 57 participants | VR-based interventions^a^, 3 (n = 32) | Conventional therapy, 3 (n = 25) | Upper extremity: SMD = 1.97 *ns* [-0.26 to 4.20] | ^d^ | Very low | Imprecision (- 2): number of participants within pooled analysis less than 100  Risk of bias (trial quality) (-1): ROB for individual biases could not be extracted, we were conservative and assumed that < 75% of participants had low ROB  Inconsistency (-1): I^2^ not reported, assumed to be greater than 75%  Risk of bias (review quality) (-2): study selection and data extraction was not performed in duplicate; no comprehensive literature search; status of publication (i.e., grey literature) was not used as an inclusion criteria |
| Chen et al., 2015 | RCTs, 2 *(2)*, 46 participants | VR-based interventions^a^, 2 (n = 23) | Conventional therapy, 2 (n = 23) | Upper extremity motor function (Fugl Myer Assessment): SMD = 0.50 *ns* [-0.09 to 1.09] | 0.00 | Low | Imprecision (- 2): number of participants within pooled analysis less than 100  Risk of bias (review quality) (-2): no comprehensive literature search; status of publication (i.e., grey literature) was not used as an inclusion criteria |
| Chen et al., 2018 | RCT, 12 *(13)*, 366 participants | VR-based interventions alone, 13 (n = 193) | Conventional therapy, 13 (n = 173) | Arm function: SMD = 0.83^ss^ [0.38 to 1.28] in favor of VR | 75.00 | Low | (-1): ROB for individual biases was not reported for this outcome, total score of risk of bias indicates that < 75% of participants had low ROB  Inconsistency (-1): I^2^ greater than 75% and *p* value significant  Risk of bias (review quality) (-2): no comprehensive literature search; status of publication (i.e., grey literature) was not used as an inclusion criteria |
| Da-Silva et al., 2018 | RCTs, 2 *(2)*, 231 participants | VR-based interventions alone, 2 (n = 113) | Conventional therapy, 1 (n = 108); No intervention, 1 (n = 10) | Arm function: SMD = -0.11 *ns* [−0.37 to 0.15] | 0.00 | Low | Risk of bias (trial quality) (-1): 2 RCT at high risk or unclear risk of detection bias  Risk of bias (review quality) (-2): no comprehensive literature search; status of publication (i.e., grey literature) was not used as an inclusion criteria |
| Domínguez-Téllez et al., 2019b | RCTs, 9 *(9)*, 302 participants | VR-based interventions with conventional therapy, 6 (n = 98); VR-based interventions with alone, 3 (n = 56) | Conventional therapy, 9 (n = 148) | Fugl Meyer: SMD = 1.53** [0.51 to 2.54] in favor of VR | 92.00 | Very low | Inconsistency (-1): I^2^ greater than 75%  Risk of bias (trial quality) (-1): ROB for individual biases could not be extracted, we were conservative and assumed that < 75% of participants had low ROB  Risk of bias (review quality) (-2): no comprehensive literature search; status of publication (i.e., grey literature) was not used as an inclusion criteria |
| Domínguez-Téllez et al., 2019b | RCTs, 5 *(5)*, 336 participants | VR-based interventions with conventional therapy, 4 (n = 162); VR-based interventions with alone, 1 (n = 10) | Conventional therapy, 5 (n = 164) | Box and Block Test: SMD = 0.55 *ns* [-0.66 to 1.76] | 95.00 | Very low | Inconsistency (-1): I^2^ greater than 75%  Risk of bias (trial quality) (-1): ROB for individual biases could not be extracted, we were conservative and assumed that < 75% of participants had low ROB  Risk of bias (review quality) (-2): no comprehensive literature search; status of publication (i.e., grey literature) was not used as an inclusion criteria |
| Johansen et al., 2020 | RCTs, 7 *(7)*, 222 participants | VR-based interventions with conventional therapy, 7 (n = 113) | Conventional therapy, 7 (n = 109) | Hand and arm function: SMD = 0.89** [0.22 to 1.56] in favor of VR | 80.00 | Low | Inconsistency (-1): I^2^ greater than 75%  Risk of bias (trial quality) (-1): 4 RCTs at unclear or high risk of selection bias (sequence generation, allocation concealment); 3 RCTs at unclear or high risk of detection bias; 3 RCTs at high risk of reporting bias  Risk of bias (review quality) (-2): no comprehensive literature search; status of publication (i.e., grey literature) was not used as an inclusion criteria |
| Karamians et al., 2019 | Pre-post design, 9^b^ *(9)*; RCTs, 29^b^ *(31)* | VR-based interventions^a,b^ | Conventional therapy, 29^b^, no control group due to pre-port design, 9^b^ | Percent of possible improvement in upper extremity: 28.45^ss^ [24.40 to 32.49] in favor of VR | 38.60 | Low | Risk of bias (trial quality) (-1): 12 RCTs at high risk of selection bias (sequence generation); 12 RCTs at high risk of attrition bias  Risk of bias (review quality) (-2): no comprehensive literature search; status of publication (i.e., grey literature) was not used as an inclusion criteria; data extraction was not performed in duplicate |
| Karamians et al., 2019 | RCTs, 29^b^ *(29)* | VR-based interventions^a,b^ | Conventional therapy, 29^b^ | Difference in Percent Possible Improvement in upper extremity function: 10.40^ss^ [5.65 to 15.14] in favor of VR | 0.00 | Low | Risk of bias (trial quality) (-1): 12 RCTs at high risk of selection bias (sequence generation); 12 RCTs at high risk of attrition bias  Risk of bias (review quality) (-2): no comprehensive literature search; status of publication (i.e., grey literature) was not used as an inclusion criteria; data extraction was not performed in duplicate |
| Laver et al., 2017 | RCTs, 17 *(17)*, 599 participants | VR-based interventions alone, 17 (n = 316) | Conventional therapy, 17 (n = 283) | Upper limb function (Fugl Meyer): MD = 2.85** [1.06 to 4.65] in favor of VR | 30.00 | High | No downgrade |
| Laver et al., 2017 | RCTs, 6 *(6)*, 266 participants | VR-based interventions alone, 6 (n = 134) | Conventional therapy, 6 (n = 132) | Grip strength: SMD = -0.02 *ns* [-0.27 to 0.22] | 44.00 | High | No downgrade |
| Laver et al., 2017 | RCTs, 5 *(5)*, 161 participants | VR-based interventions alone, 5 (n = 84) | Conventional therapy, 5 (n = 77) | Upper limb function, amount of use (subjective): MD = -0.11 *ns* [-0.42 to 0.21] | 0.00 | Moderate | Imprecision (- 1): number of participants within pooled analysis less than 200 |
| Lee et al., 2019 | RCT, 9 *(53)*, 232 participants | VR-based interventions with conventional therapy, 5 (n = 65); VR-based interventions with alone, 4 (n = 53) | Conventional therapy, 8 (n = 109); No intervention, 1 (n = 5) | Upper limb function: Hedge’s *g* = 0.43*** [0.42 to 0.53] in favor of VR | ^d^ | Low | Inconsistency (-1): I^2^ not reported, assumed to be greater than 75%  Risk of bias (review quality) (-2): no comprehensive literature search; status of publication (i.e., grey literature) was not used as an inclusion criteria; data extraction was not performed in duplicate |
| Lin et al., 2018 | RCTs, 6 *(6)*, 522 participants | VR-based interventions with conventional therapy, 5 (n = 224), VR-based interventions alone, 1 (n = 35) | Conventional therapy, 6 (n = 263) | Upper extremity: SMD = -0.04 *ns* [-0.21 to 0.13] | 0.00 | Low | Risk of bias (trial quality) (-1): ROB for individual studies could not be extracted, we were conservative and assumed that < 75% of participants had low ROB  Risk of bias (review quality) (-2): no comprehensive literature search; status of publication (i.e., grey literature) was not used as an inclusion criteria |
| Lin et al., 2018 | RCTs, 3 *(3)*, 115 participants | VR-based interventions with conventional therapy, 3 (n = 56) | No intervention, 3 (n = 59) | Upper extremity: SMD = 0.23 *ns* [-0.14 to 0.60] | 0.00 | Low | Imprecision (- 1): number of participants within pooled analysis less than 200  Risk of bias (review quality) (-2): no comprehensive literature search; status of publication (i.e., grey literature) was not used as an inclusion criteria |
| Mekbib et al., 2020 | RCTs, 20 *(20)*, 708 participants | VR-based interventions with with conventional therapy, 19 (n = 356; VR-based interventions alone, 1 (n = 7) | Conventional therapy, 20 (n = 345) | Fugl Meyer: MD = 3.84** [0.93 to 6.75] in favor of VR | 64.00 | Low | Risk of bias (review quality) (-2): study selection was not performed in duplicate; no comprehensive literature search; status of publication (i.e., grey literature) was not used as an inclusion criteria |
| Mekbib et al., 2020 | RCTs, 13 *(13)*, 551 participants | VR-based interventions with with conventional therapy, 12 (n = 269; VR-based interventions alone, 1 (n = 9) | Conventional therapy, 13 (n = 273) | Box and Block test: MD = 3.82* [0.26 to 7.38] in favor of VR | 64.00 | Low | Risk of bias (review quality) (-2): study selection was not performed in duplicate; no comprehensive literature search; status of publication (i.e., grey literature) was not used as an inclusion criteria |
| Mekbib et al., 2020 | RCTs, 6 *(6)*, 124 participants | VR-based interventions with with conventional therapy, 5 (n = 54; VR-based interventions alone, 1 (n = 9) | Conventional therapy, 6 (n = 61) | Motor Activity Log: MD = 0.80** [0.44 to 1.15] in favor of VR | 64.00 | Low | Risk of bias (review quality) (-2): study selection was not performed in duplicate; no comprehensive literature search; status of publication (i.e., grey literature) was not used as an inclusion criteria  Imprecision (- 1): number of participants within pooled analysis less than 200 |
| Rutkowski et al., 2020 | RCTs, 7 *(7)*, 173 participants | VR-based interventions alone, 7 (n = 90) | Conventional therapy, 7 (n = 83) | Upper limb function: SMD = 0.80 *ns* [-0.19 to 1.80] | 88.00 | Very low | Imprecision (- 1): number of participants within pooled analysis less than 200  Risk of bias (review quality) (-2): no comprehensive literature search; status of publication (i.e., grey literature) was not used as an inclusion criteria |
| Rutkowski et al., 2020 | RCTs, 5 *(5)*, 138 participants | VR-based interventions alone, 5 (n = 72) | Conventional therapy, 5 (n = 66) | Upper limb function (Fugl Meyer): MD = 8.41** [7.13 to 9.68] in favor of VR | 46.00 | Very low | Imprecision (- 1): number of participants within pooled analysis less than 200  Risk of bias (review quality) (-2): no comprehensive literature search; status of publication (i.e., grey literature) was not used as an inclusion criteria |
| Rutkowski et al., 2020 | RCTs, 5 *(5)*, 110 participants | VR-based interventions alone, 5 (n = 57) | Conventional therapy, 5 (n = 53) | Upper limb hand dexterity function SMD = 0.98 *ns* [-0.51 to 2.47] | 91.00 | Very low | Imprecision (- 1): number of participants within pooled analysis less than 200  Risk of bias (review quality) (-2): no comprehensive literature search; status of publication (i.e., grey literature) was not used as an inclusion criteria |
| Saposnik et al., 2011 | RCTs, 5 *(5)*, 84 participants | VR-based interventions with with conventional therapy, 1 (n = 10; VR-based interventions alone, 4 (n = 32) | Conventional therapy, 5 (n = 42) | Improvement of motor impairment: OR = 4.89* [1.31 to 18.3] in favor of VR | 0.00 | Very low | Imprecision (- 2): number of participants within pooled analysis less than 100  Risk of bias (trial quality) (-1): ROB for individual trials was not reported within the review, we were conservative and assumed that less than 75% of participants had low ROB  Risk of bias (review quality) (-2): study selection and data extraction was not performed in duplicate; no comprehensive literature search; status of publication (i.e., grey literature) was not used as an inclusion criteria |
| Saposnik et al., 2011 | Pre-post design, 5 *(5)*, 90 participants | VR-based interventions alone, 5 (n = 90) | n/a | Improvement of motor impairment: SMD = 0.14*** [0.08 to 0.23] in favor of post-test after VR | 0.00 | Low | Imprecision (- 2): number of participants within pooled analysis less than 100  Risk of bias (review quality) (-2): study selection and data extraction was not performed in duplicate; no comprehensive literature search; status of publication (i.e., grey literature) was not used as an inclusion criteria |
| Saposnik et al., 2011 | Pre-post design, 6 *(6)*, 49 participants | VR-based interventions alone, 6 (n = 49) | n/a | Improvement of motor function: SMD = 0.20*** [0.11 to 0.33] in favor of post-test after VR | 0.00 | Low | Imprecision (- 2): number of participants within pooled analysis less than 100  Risk of bias (review quality) (-2): study selection and data extraction was not performed in duplicate; no comprehensive literature search; status of publication (i.e., grey literature) was not used as an inclusion criteria |
| Saposnik et al., 2011 | RCTs, 2 *(2)*, 30 participants | VR-based interventions with conventional therapy, (n = 10), VR-based interventions alone, 1 (n = 5) | Conventional therapy, 2 (n = 15) | Motor function (Box and Block Test): OR = 0.49 *ns* [0.09 to 2.65] | 0.00 | Very low | Imprecision (- 2): number of participants within pooled analysis less than 100  Risk of bias (trial quality) (-1): ROB for individual trials was not reported within the review, we were conservative and assumed that less than 75% of participants had low ROB  Risk of bias (review quality) (-2): study selection and data extraction was not performed in duplicate; no comprehensive literature search; status of publication (i.e., grey literature) was not used as an inclusion criteria |
| Saposnik et al., 2011 | RCTs, 3 *(3)*, 40 participants | VR-based interventions with conventional therapy, 1 (n = 10); VR-based interventions alone, 2 (n = 10) | Conventional therapy, 3 (n = 20) | Motor function (Wolf motor function test, manual function): OR = 1.29 *ns* [0.28 to 5.90] | 0.00 | Very low | Imprecision (- 2): number of participants within pooled analysis less than 100  Risk of bias (trial quality) (-1): ROB for individual trials was not reported within the review, we were conservative and assumed that less than 75% of participants had low ROB  Risk of bias (review quality) (-2): study selection and data extraction was not performed in duplicate; no comprehensive literature search; status of publication (i.e., grey literature) was not used as an inclusion criteria |
| Saywell et al., 2017 | RCTs, 9 *(9)*, 267 participants | VR-based interventions with conventional therapy, non-dose matched, 4 (n = 59), VR-based interventions alone, 5 (n = 77) | Conventional therapy, 8 (n = 126); No intervention, 1 (n = 5) | Upper limb: SMD = 0.29 *ns* [0.04 to 0.54] | 0.00 | Low | Risk of bias (trial quality) (-1): 4 RCTs at high risk of selection bias (sequence generation); 1 RCT at high risk of detection bias  Risk of bias (review quality) (-2): no comprehensive literature search; status of publication (i.e., grey literature) was not used as an inclusion criteria |
| Saywell et al., 2017 | RCTs, 7 *(7)*, 228 participants | VR-based interventions with conventional therapy,2 (n = 23); VR-based interventions alone, 5 (n = 84) | Conventional therapy, 2 (n = 33); No intervention, 5 (n = 84) | Fugl Meyer: SMD = 0.39^ss^ [0.13 to 0.65] in favor of VR | 0.00 | Moderate | Risk of bias (review quality) (-2): no comprehensive literature search; status of publication (i.e., grey literature) was not used as an inclusion criteria |
| Tay et al., 2018 | RCTs, 10 *(10)*, 241 participants | VR-based interventions with conventional therapy, 6 (n = 92); VR-based interventions alone, 4 (n = 30) | Conventional therapy, 9 (n = 114); Control group, 1 (n = 5) | Upper limb functioning (Fugl Meyer): SMD = 0.46** [0.20 to 0.70] in favor of VR | 0.00 | Low | Risk of bias (trial quality) (-1): ROB for individual trials was not reported within the review, we were conservative and assumed that less than 75% of participants had low ROB  Risk of bias (review quality) (-2): no comprehensive literature search; status of publication (i.e., grey literature) was not used as an inclusion criteria |
| Tay et al., 2018 | RCTs, 3 *(3)*, 46 participants | VR-based interventions with conventional therapy, 2 (n = 17); VR-based interventions alone, 1 (n = 6) | Conventional therapy, 3 (n = 23) | Upper limb gross movements and activity level (Box and Block Test): MD = 1.46 *ns* [−9.60 to 12.53] | 0.00 | Very low | Imprecision (- 2): number of participants within pooled analysis less than 100  Risk of bias (trial quality) (-1): ROB for individual biases could not be extracted for individual studies, we were conservative and assumed that < 75% of participants had low ROB  Risk of bias (review quality) (-2): no comprehensive literature search; status of publication (i.e., grey literature) was not used as an inclusion criteria |
| Veerbeek et al., 2014 | RCTs, 8 *(8)*, 158 participants | VR-based interventions^a^, 8^b^ | Control group^c^, 8^b^ | Motor function arm (synergy): Hedge’s *g* = 0.19 *ns* [-0.10 to 0.48] | 0.00 | Low | Imprecision (- 1): number of participants within pooled analysis less than 200  Risk of bias (trial quality) (-1): ROB for individual studies could not be extracted, we were conservative and assumed that < 75% of participants had low ROB  Risk of bias (review quality) (-2): status of publication (i.e., grey literature) was not used as an inclusion criteria; study selection and data extraction was not performed in  duplicate |
| Veerbeek et al., 2014 | RCTs, 6 *(6)*, 89 participants | VR-based interventions^a^, 6^b^ | Control group^c^, 6^b^ | Arm-hand activities (unilateral): Hedge’s *g* = 0.16 *ns* [-0.21 to 0.53] | 0.00 | Very low | Imprecision (- 2): number of participants within pooled analysis less than 100  Study limitations (-1): ROB for individual studies could not be extracted, we were conservative and assumed that < 75% of participants had low ROB  Risk of bias (review quality) (-2): status of publication (i.e., grey literature) was not used as an inclusion criteria; study selection and data extraction was not performed in duplicate |
| Veerbeek et al., 2014 | RCTs^a^, 44 participants | VR-based interventions ^a,b^ | Conventional therapy^b,c^, Control group^b,c^ | Muscle tone: Hedge’s *g* = -0.64^ss^ [-1.15 to -0.12] in favour of control intervention | 0.00 | Very low | Imprecision (- 2): number of participants within pooled analysis less than 100  Risk of bias (trial quality) (-1): ROB for individual studies could not be extracted, we were conservative and assumed that < 75% of participants had low ROB  Risk of bias (review quality) (-2): status of publication (i.e., grey literature) was not used as an inclusion criteria; study selection and data extraction was not performed in duplicate |
| Veerbeek et al., 2014 | RCTs^a^, 38 participants | VR-based interventions ^a,b^ | Conventional therapy^b,c^, Control group^b,c^ | Arm-hand activities (bilateral): Hedge’s *g* = 0.47 *ns* [-0.42 to 1.37] | 50.00 | Very low | Imprecision (- 2): number of participants within pooled analysis less than 100  Risk of bias (trial quality) (-1): ROB for individual studies could not be extracted, we were conservative and assumed that < 75% of participants had low ROB  Risk of bias (review quality) (-2): status of publication (i.e., grey literature) was not used as an inclusion criteria; study selection and data extraction was not performed in duplicate |
| Wattchow et al., 2018 | RCTs, 2 *(2)*, 27 participants | VR-based interventions with conventional therapy, 2^b^ | Conventional therapy, 2^b^ | UL activity: SMD = 0.15 *ns* [-0.41 to 0.72] | 0.00 | Low | Imprecision (- 2): number of participants within pooled analysis less than 100  Risk of bias (trial quality) (-1): ROB for individual studies could not be extracted, we were conservative and assumed that < 75% of participants had low ROB  Risk of bias (review quality) (-1): status of publication (i.e., grey literature) was not used as an inclusion criteria |
| Wattchow et al., 2018 | RCTs, 2 *(2)*, 27 participants | VR-based interventions alone, 2^b^ | Conventional therapy, 2^b^ | UL impairment (Fugl-Meyer Assessment): MD = 4.54 *ns* [-6.07 to 15.15] | 0.00 | Low | Imprecision (- 2): number of participants within pooled analysis less than 100  Risk of bias (trial quality) (-1): ROB for individual studies could not be extracted, we were conservative and assumed that < 75% of participants had low ROB  Risk of bias (review quality) (-1): status of publication (i.e., grey literature) was not used as an inclusion criteria |

*Note.* *** *p* < .001, ** *p* < .01, * *p* < .05, *ns*- non-significant results. ^a^ = we were not able to identify whether VR-based interventions were delivered alone or in combination with conventional therapy; ^b^ = number of participants in the intervention/control group could not be extracted; ^c^ = control group type not specified; ^d^ = I^2^ value not reported; Hedge’s *g* = Hedge’s *g* coefficient, effect size; n = sample size; n/a = not applicable, pre-test post-test design; MD = Mean Difference, effect size; OR = Odds Ratio, effect size; RCT = randomized controlled trial; SMD = Standardized Mean Difference, effect size; ^ss^ = statistically significant results, without *p* value provided by authors

Supplementary Table S7

*Details of included reviews. Reported effects of interventions and quality of evidence for reported outcome**s: Activity limitation*

| Author(s), year | Design, number of studies *(number of effects included in the analysis)*, total number of participants per outcome | Intervention | Control or comparison intervention | Effect measure (95% CI) | Heterogeneity | Quality of the evidence | Reason for quality of evidence ratings |
| --- | --- | --- | --- | --- | --- | --- | --- |
| Ahn et al., 2019 | RCTs, 9 *(9)*, 694 participants | VR-based interventions with conventional therapy, 2 (n = 121); VR-based interventions alone, 7 (n = 157) | Conventional therapy, 8 (n = 408); Control group not specified, 1 (n = 8) | Activities of daily living: SMD = 0.41** [0.25 to 0.57] in favor of VR | ^d^ | Low | Risk of bias (trial quality) (-1): 8 RCTs at high risk of selection bias (sequence generation)  Risk of bias (review quality) (-2): no comprehensive literature search; status of publication (i.e., grey literature) was not used as an inclusion criteria; data extraction was not performed in duplicate |
| Domínguez-Téllez et al., 2019b | RCTs, 5 *(5)*, 407 participants | VR-based interventions with conventional therapy, 4 (n = 171); VR-based interventions with alone, 1 (n = 33) | Conventional therapy, 5 (n = 203) | Functional Independence Measure: SMD = 0.77* [0.05 to 1.49] in favor of VR | 91.00 | Very low | Inconsistency (-1): I^2^ greater than 75%  Risk of bias (trial quality) (-1): ROB for individual biases could not be extracted, we were conservative and assumed that < 75% of participants had low ROB  Risk of bias (review quality) (-2): no comprehensive literature search; status of publication (i.e., grey literature) was not used as an inclusion criteria |
| Chen et al., 2015 | RCTs, 2 *(2)*, 114 participants | VR-based interventions^a^, 2 (n = 56) | Conventional therapy, 2 (n = 58) | Activities of daily living (Barthel Index Scale): SMD = -0.37 *ns* [-0.74 to 0.00] | 0.00 | Low | Imprecision (- 1): number of participants within pooled analysis less than 200  Risk of bias (trial quality) (-1) ROB less than 75% of participants had low ROB; 1 RCT at unclear risk and 1 RCT at low risk  Risk of bias (review quality) (-2): no comprehensive literature search; status of publication (i.e., grey literature) was not used as an inclusion criteria |
| Cheok et al., 2015 | RCTs, 2 *(2)*, 37 participants | VR-based interventions with conventional therapy, 2 (n = 20) | Conventional therapy, 2 (n = 17) | Global function (Functional Independence Measure): SMD = 0.27 *ns* [-0.38 to 0.93] | 0.00 | Low | Imprecision (- 2): number of participants within pooled analysis less than 200  Risk of bias (review quality) (-2): no comprehensive literature search; status of publication (i.e., grey literature) was not used as inclusion criteria |
| Da-Silva et al., 2018 | RCTs, 1 *(1)*, 22 participants | VR-based interventions alone, 1 (n = 12) | No intervention, 1 (n = 10) | Perceived amount of use of the stroke arm: MD = -0.13 *ns* [-1.15 to 0.89] | n/a | Very low | Imprecision (- 2): number of participants within pooled analysis less than 200  Risk of bias (trial quality) (-1): 1 RCT at unclear risk of detection bias  Risk of bias (review quality) (-2): no comprehensive literature search; status of publication (i.e., grey literature) was not used as an inclusion criteria |
| Da-Silva et al., 2018 | RCTs, 1 *(1)*, 22 participants | VR-based interventions alone, 1 (n = 12) | No intervention, 1 (n = 10) | Perceived quality of use of the stroke arm: MD = 1.25^ss^ [0.27 to 2.23] in favour of VR | n/a | Very low | Imprecision (- 2): number of participants within pooled analysis less than 100  Risk of bias (trial quality) (-1): 1 RCT at unclear risk of detection bias  Risk of bias (review quality) (-2): no comprehensive literature search; status of publication (i.e., grey literature) was not used as an inclusion criteria |
| Domínguez-Téllez et al., 2019b | RCTs, 4 *(4)*, 273 participants | VR-based interventions with conventional therapy, 3 (n = 127); VR-based interventions with alone, 1 (n = 9) | Conventional therapy, 9 (n = 137) | Modified Barthel Index: SMD = 2.37 *ns* [-0.25 to 4.98] | 98.00 | Very low | Inconsistency (-1): I^2^ greater than 75%  Risk of bias (trial quality) (-1): ROB for individual biases could not be extracted, we were conservative and assumed that < 75% of participants had low ROB  Risk of bias (review quality) (-2): no comprehensive literature search; status of publication (i.e., grey literature) was not used as an inclusion criteria |
| Saywell et al., 2017 | RCTs, 3 *(3)*, 114 participants | VR-based interventions with conventional therapy, dose matched, 3 (n = 58) | Conventional therapy, 3 (n = 56) | Independence: SMD = 0.60^ss^ [0.23 to 0.97] in favor of VR | 0.00 | Low | Imprecision (- 1): number of participants within pooled analysis less than 200  Risk of bias (trial quality) (-1): 3 RCTs at high risk of selection bias (sequence generation)  Risk of bias (review quality) (-2): no comprehensive literature search; status of publication (i.e., grey literature) was not used as an inclusion criteria |
| Veerbeek et al., 2014 | RCTs^a^, 63 participants | VR-based interventions^b,c^ | Conventional therapy^c,d^ Control group^c,d^ | Basic activities of daily living: Hedge’s *g* = 0.80^ss^ [0.29 to 1.30] in favor of VR | 0.00 | Very low | Imprecision (- 2): number of participants within pooled analysis less than 100  Risk of studies (trial quality) (-1): ROB for individual trials could not be estimated as the trials were not fully described for this outcome, we were conservative and assumed that less than 75% of participants had low ROB  Risk of bias (review quality) (-2): status of publication (i.e., grey literature) was not used as an inclusion criteria; study selection and data extraction was not performed in duplicate |

*Note.* *** *p* < .001, ** *p* < .01, * *p* < .05, *ns*- non-significant results. ^a^ = we were not able to identify whether VR-based interventions were delivered alone or in combination with conventional therapy; ^b^ = number of participants in the intervention/control group could not be extracted; ^c^ = control group type not specified; ^d^ = I^2^ value not reported; n = sample size; RCT = randomized controlled trial; SMD = Standardized Mean Difference, effect size

Supplementary Table S8

*Details of included reviews. Reported effects of interventions and quality of evidence for reported outcomes: ICF WHO Framework: body structures/function, activity and participation*

| Author(s), year | Design, number of studies *(number of effects included in the analysis)*, total number of participants per outcome | Intervention | Control or comparison intervention | Effect measure (95% CI) | Heterogeneity | Quality of the evidence | Reason for quality of evidence ratings |
| --- | --- | --- | --- | --- | --- | --- | --- |
| Aminov et al., 2018 | RCTs, 27 *(27)*, NS | VR-based interventions^a^, 27^b^ | Control group^c^, 27^b^ | Body Structures/Function: Hedges *g* = 0.41** [0.28 to 0.55] in favor of VR | 0.00 | Low | Risk of bias (trial quality) (-1): ROB for individual studies could not be extracted, we were conservative and assumed that < 75% of participants had low ROB  Risk of bias (review quality) (-2): no comprehensive literature search; status of publication (i.e., grey literature) was not used as an inclusion criteria |
| Aminov et al., 2018 | RCTs, 29 *(29)*, NS | VR-based interventions^a^, 29^b^ | Control group^c^, 29^b^ | Activity: Hedge’s *g* = 0.47 ** [0.34 to 0.60] in favor of VR | 0.00 | Low | Risk of bias (trial quality) (-1): ROB for individual studies could not be extracted, we were conservative and assumed that < 75% of participants had low ROB  Risk of bias (review quality) (-2): no comprehensive literature search; status of publication (i.e., grey literature) was not used as an inclusion criteria |
| Aminov et al., 2018 | RCTs, 5 *(5)*, NS | VR-based interventions^a^, 5^b^ | Control group^c^, 5^b^ | Participation: Hedge’s *g* = 0.38 *ns* [-0.29-1.04] | 65.06 | Low | Risk of bias (trial quality) (-1): ROB for individual studies could not be extracted, we were conservative and assumed that < 75% of participants had low ROB  Risk of bias (review quality) (-2): no comprehensive literature search; status of publication (i.e., grey literature) was not used as an inclusion criteria |
| Chen et al., 2014 | Pre- and post VR design, 14 *(14)*, 122 participants | VR-based interventions^a^, 14^b^ | n/a | Participation: SMD = 1.92^ss^ [1.19 to 2.66] in favour of post-test after VR | ^d^ | Very low | Imprecision (- 1): number of participants within pooled analysis less than 200  (-1): ROB for individual studies could not be extracted, we were conservative and assumed that < 75% of participants had low ROB  Inconsistency (-1): I^2^ not reported, assumed to be greater than 75%  Risk of bias (review quality) (-2): study selection and data extraction was not performed in duplicate; no comprehensive literature search; status of publication (i.e., grey literature) was not used as an inclusion criteria |
| Chen et al., 2014 | Pre- and post VR design, 14 *(14)*, 122 participants | VR-based interventions^a^, 14^b^ | n/a | Activity: SMD = 0.46 *ns* [-0.08 to 1.16] | ^d^ | Very low | Imprecision (- 1): number of participants within pooled analysis less than 200  Risk of bias (trial quality) (-1): ROB for individual studies could not be extracted, we were conservative and assumed that < 75% of participants had low ROB  Inconsistency (-1): I^2^ not reported, assumed to be greater than 75%  Risk of bias (review quality) (-2): study selection and data extraction was not performed in duplicate; no comprehensive literature search; status of publication (i.e., grey literature) was not used as an inclusion criteria |
| Chen et al., 2014 | Pre- and post VR design, 14 *(14)*, 122 participants | VR-based interventions^a^, 14^b^ | n/a | Body structure and function: SMD = 0.70^ss^ [0.10 to 1.30] in favor of post-test after VR | ^d^ | Very low | Imprecision (- 1): number of participants within pooled analysis less than 200  Risk of bias (trial quality) (-1): ROB for individual studies could not be extracted, we were conservative and assumed that < 75% of participants had low ROB  Inconsistency (-1): I^2^ not reported, assumed to be greater than 75%  Risk of bias (review quality) (-2): study selection and data extraction was not performed in duplicate; no comprehensive literature search; status of publication (i.e., grey literature) was not used as an inclusion criteria |
| Chen et al., 2018 | RCTs, 19 *(19)*, 504 | VR-based interventions^a^, 19 (n = 266) | Control group, 19 (n = 248) | Activity: SMD = 0.89^ss^ [-0.53 to 1.26] in favour of VR | ^d^ | Low | Risk of bias (trial quality) (-1): ROB for individual biases was not reported for this outcome, total score of risk of bias indicates that < 75% of participants had low ROB  Inconsistency (-1): I^2^ not reported, assumed to be greater than 75%  Risk of bias (review quality) (-2): no comprehensive literature search; status of publication (i.e., grey literature) was not used as an inclusion criteria |
| Chen et al., 2018 | RCTs, 8 *(8)*, 304 | VR-based interventions^a^, 8 (n = 157) | Control group, 8 (n = 147) | Body structure and function: SMD = 0.67^ss^ [0.36 to 0.98] in favour of VR | ^d^ | Low | Risk of bias (trial quality) (-1): ROB for individual biases was not reported for this outcome, total score of risk of bias indicates that < 75% of participants had low ROB  Inconsistency (-1): I^2^ not reported, assumed to be greater than 75%  Risk of bias (review quality) (-2): no comprehensive literature search; status of publication (i.e., grey literature) was not used as an inclusion criteria |
| Chen et al., 2018 | RCTs, 3 *(3)*, 145 participants | VR-based interventions^a^, 3 (n = 78) | Control group type^c^, 3 (n = 67) | Participation: SMD = 0.40^ss^ [0.07 to 0.73] in favour of VR | ^d^ | Very low | Imprecision (- 1): number of participants within pooled analysis less than 200  Inconsistency (-1): I^2^ not reported, assumed to be greater than 75%  Risk of bias (trial quality) (-1): ROB for individual trials could not be estimated as the trials were not fully described for this outcome, we were conservative and assumed that less than 75% of participants had low ROB  Risk of bias (review quality) (-2): no comprehensive literature search; status of publication (i.e., grey literature) was not used as an inclusion criteria |
| Maier et al., 2019 | RCTs, 24 *(26)*, 1255 participants | VR-based interventions with conventional therapy, 17 (n = 594); VR-based interventions alone, 9 (n = 110) | Conventional therapy, 27 (n = 546); No intervention, 1 (n =5) | Upper limb body function: SMD = 0.21** [0.08 to 0.33] in favour if VR | 12.00 | Moderate | Risk of bias (review quality) (-2): no comprehensive literature search; status of publication (i.e., grey literature) was not used as an inclusion criteria |
| Maier et al., 2019 | RCTs, 28 *(30)*, 1407 participants | VR-based interventions with conventional therapy, 22 (n = 709); VR-based interventions alone, 8 (n = 75) | Conventional therapy, 27 (n = 609); No intervention, 2 (n =14) | Upper limb activity: SMD = 0.27** [0.13 to 0.41] in favour if VR | 30.00 | Moderate | Risk of bias (review quality) (-2): no comprehensive literature search; status of publication (i.e., grey literature) was not used as an inclusion criteria |
| Lohse et al., 2014 | RCTs, 16 *(16)*, 459 participants | VR-based interventions^a^, 16 (n = 240) | Control group^c^, 16 (n = 219) | Body function: Hedges’ *g* = 0.48** [0.27 to 0.70] in favor of VR | 24.79 | Low | Risk of bias (trial quality) (-1): high risk of selection bias (sequence generation); 5 RCTs at high risk of detection bias;  Risk of bias (review quality) (-2): no comprehensive literature search; status of publication (i.e., grey literature) was not used as an inclusion criteria |
| Lohse et al., 2014 | RCTs, 22 *(22)*, 554 participants | VR-based interventions^a^, 22 (n = 283) | Control group^c^, 22 (n = 271) | Activity: Hedges’ *g* = 0.58** [0.32 to 0.85] in favor of VR | 55.23 | Low | Risk of bias (trial quality) (-1): 14 RCTs at high risk of selection bias (sequence generation); 9 RCTs at high risk of detection bias  Risk of bias (review quality) (-2): no comprehensive literature search; status of publication (i.e., grey literature) was not used as an inclusion criteria |
| Lohse et al., 2014 | RCTs, 3 *(3)*, 74 participants | VR-based interventions^a^, 3 (n = 42) | Conventional therapy, 2 (n = 16) No intervention, 1 (n = 16) | Participation: Hedges’ *g* = 0.56* [0.02 to 1.10] in favor of VR | 26.75 | Very low | Imprecision (- 2): number of participants within pooled analysis less than 100  Risk of bias (trial quality) (-1): 2 RCTs at high risk of selection bias (sequence generation); 2 RCTs at high risk of detection bias  Risk of bias (review quality) (-2): no comprehensive literature search; status of publication (i.e., grey literature) was not used as an inclusion criteria |

*Note.* *** *p* < .001, ** *p* < .01, * *p* < .05, *ns*- non-significant results. ^a^ = we were not able to identify whether VR-based interventions were delivered alone or in combination with conventional therapy; ^b^ = number of participants in the intervention/control group could not be extracted; ^c^ = control group type not specified; ^d^ = I^2^ value not reported; Hedge’s *g* = Hedge’s *g* coefficient, effect size; n = sample size; n/a = not applicable, pre-test post-test design; RCT = randomized controlled trial; SMD = Standardized Mean Difference, effect size; ^ss^ = statistically significant results, without *p* value provided by authors

Supplementary Table S9

*Details of included reviews. Reported effects of interventions and quality of evidence for reported outcomes: Motor function*

| Author(s), year | Design and control/intervention number of studies included in the meta-analysis | Intervention | Control or comparison intervention | Effect measure (95% CI) | Heterogeneity | Quality of the evidence | Reason for quality of evidence ratings |
| --- | --- | --- | --- | --- | --- | --- | --- |
| Ghai et al., 2019 | Pre-post design, 4 *(4)*, 53 participants | VR-based interventions alone, 4 (n = 53) | n/a | Gross motor function: Hedge’s *g* = 0.44^ss^ [0.06 to 0.83] in favor of post-test after VR | 54.00 | Low | Imprecision (- 2): number of participants within pooled analysis less than 100  Risk of bias (review quality) (-2): no comprehensive literature search; status of publication (i.e., grey literature) was not used as an inclusion criteria; data extraction was not performed in duplicate  Risk of bias (trial quality) (-1): 3 RCTs at high risk of detection bias |
| Gibbons et al., 2016 | RCTs, 2 *(2)*, 80 participants | VR-based interventions^a^, 2 (n = 41) | Conventional therapy, 2 (n = 39) | Motor function for acute-subacute stroke: SMD = 0.20 *ns* [-0.92 to 1.31] | 79.00 | Very low | Imprecision (- 2): number of participants within pooled analysis less than 100  Inconsistency (-1): I^2^ greater than 75%  Risk of bias (trial quality) (-1): 1 RCT at high risk of selection bias (sequence generation); 1 RCT at high risk of detection bias  Inconsistency (-1): I^2^ greater than 75% and *p* value significant  Risk of bias (review quality) (-2): no comprehensive literature search; status of publication (i.e., grey literature) was not used as an inclusion criteria |
| Gibbons et al., 2016 | RCTs, 3 *(3)*, 71 participants | VR-based interventions^a^, 2 (n = 37) | Conventional therapy, 2 (n = 34) | Motor function for chronic stroke: SMD = 0.27 *ns* [-0.63 to 1.17] | 70.00 | Low | Imprecision (- 2): number of participants within pooled analysis less than 100  Risk of bias (review quality) (-2): no comprehensive literature search; status of publication (i.e., grey literature) was not used as an inclusion criteria |
| Laver et al., 2017 | RCTs, 3 *(3)*, 43 participants | VR-based interventions with conventional therapy, 3 (n = 23) | Conventional therapy, 1 (n = 8), No intervention, 2 (n = 12) | Global motor function: SMD = 0.01 *ns* [-0.60 to 0.61] | 0.00 | Moderate | Imprecision (- 2): number of participants within pooled analysis less than 100 |

*Note.* *** *p* < .001, ** *p* < .01, * *p* < .05, *ns*- non-significant results. ^a^ = we were not able to identify whether VR-based interventions were delivered alone or in combination with conventional therapy; Hedge’s *g* = Hedge’s *g* coefficient, effect size; n = sample size; n/a = not applicable; RCT = randomized controlled trial; SMD = Standardized Mean Difference, effect size; ^ss^ = statistically significant results, without *p* value provided by authors

Supplementary Table S10

*Details of included reviews. Reported effects of interventions and quality of evidence for reported outcomes: Cognitive functioning*

| Author(s), year | Design and control/intervention number of studies included in the meta-analysis | Intervention | Control or comparison intervention | Effect measure (95% CI) | Heterogeneity | Quality of the evidence | Reason for quality of evidence ratings |
| --- | --- | --- | --- | --- | --- | --- | --- |
| Aminov et al., 2018 | RCTs, 4 *(4)*, 86 participants | VR-based interventions with conventional therapy, 4 (n = 45) | Conventional therapy, 4 (n = 41) | Cognition: Hedges *g* = 0.45* [0.02 to 0.88] in favor of VR | 14.69 | Very low | Imprecision (- 2): number of participants within pooled analysis less than 100  Risk of bias (trial quality) (-1): 3 RCTs at high risk of selection bias (sequence generation); 3 RCTs at high risk of detection bias  Risk of bias (review quality) (-2): no comprehensive literature search; status of publication (i.e., grey literature) was not used as an inclusion criteria |
| Wiley et al., 2020 | RCTs, 5 *(5)*, 122 participants | VR-based interventions with conventional therapy, 1 (n = 10); VR-based interventions with alone, 4 (n = 53) | Conventional therapy, 4 (n = 59) | Global cognition: SMD = 0.24 *ns* [-0.30 to 0.78] | 53.00 | Very low | Imprecision (- 2): number of participants within pooled analysis less than 100  Risk of bias (review quality) (-1): status of publication (i.e., grey literature) was not used as an inclusion criteria |
| Wiley et al., 2020 | RCTs, 2 *(2)*, 46 participants | VR-based interventions with alone, 2 (n = 24) | Conventional therapy, 2 (n = 22) | Attention (TMT-A): MD = 8.90 *ns* [-27.89 to 45.70] | 49.00 | Very low | Imprecision (- 2): number of participants within pooled analysis less than 100  Risk of bias (review quality) (-1): status of publication (i.e., grey literature) was not used as an inclusion criteria |
| Wiley et al., 2020 | RCTs, 2 *(2)*, 46 participants | VR-based interventions with alone, 2 (n = 24) | Conventional therapy, 2 (n = 22) | Memory: SMD = 0.00 *ns* [-0.58 to 0.59] | 0.00 | Very low | Imprecision (- 2): number of participants within pooled analysis less than 100  Risk of bias (review quality) (-1): status of publication (i.e., grey literature) was not used as an inclusion criteria |
| Wiley et al., 2020 | RCTs, 2 *(2)*, 39 participants | VR-based interventions with conventional therapy, 1 (n = 10); VR-based interventions with alone, 1 (n = 9) | Conventional therapy, 2 (n = 20) | Language: SMD = 0.56 *ns* [-0.08 to 1.21] | 0.00 | Very low | Imprecision (- 2): number of participants within pooled analysis less than 100  Risk of bias (review quality) (-1): status of publication (i.e., grey literature) was not used as an inclusion criteria |

*Note.* *** *p* < .001, ** *p* < .01, * *p* < .05, *ns*- non-significant results. Hedge’s *g* = Hedge’s *g* coefficient, effect size; MD = Mean Difference, effect size; n = sample size; RCT = randomized controlled trial; SMD = Standardized Mean Difference, effect size

Supplementary Table S11

*Reported effects for follow-up*

| Author(s), year | Design, number of studies, total number of participants per comparison | Intervention, number of studies and participants per comparison | Control, number of studies and participants per comparison | Outcomes and effect measure (95% CI) | Heterogeneity (I^2^) | Quality of the evidence | Reason for quality of evidence ratings |
| --- | --- | --- | --- | --- | --- | --- | --- |
| Aminov et al., 2018 | RCTs, 12^a^ *(12)* | VR-based interventions, 12^b,c^ | Conventional therapy, 12^c^ | Motor, functional, and cognitive outcomes combined: Hedge’s *g* = 0.46** [0.21 to 0.68] in favor of VR | 27.14 | Low | Risk of bias (trial quality) (-1): ROB for individual studies could not be extracted, we were conservative and assumed that < 75% of participants had low ROB  Risk of bias (review quality) (-2): no comprehensive literature search; status of publication (i.e., grey literature) was not used as an inclusion criteria |
| Corbetta et al., 2015 | RCT, 3 *(3)*, 54 participants | VR-based interventions alone, 3 (n = 28) | Conventional therapy, 3 (n = 26) | Walking speed: MD = 0.12** [0.03 to 0.20] in favor of VR | 0.00 | Very low | Imprecision (- 2): number of participants within pooled analysis less than 100  Risk of bias (trial quality) (-1): 1 RCT at unclear risk of selection bias (sequence generation); 1 RCT at unclear risk of detection bias  Risk of bias (review quality) (-2): no comprehensive literature search; status of publication (i.e., grey literature) was not used as an inclusion criteria |
| Gibbons et al., 2016 | RCTs, 2 *(2)*, 36 participants | VR-based interventions alone, 2 (n = 19) | Conventional therapy, 2 (n = 17) | Gait velocity for chronic stroke: MD = -0.12* [-0.22 to -0.02] in favour of control | 0.00 | Low | Imprecision (- 2): number of participants within pooled analysis less than 100  Risk of bias (review quality) (-2): no comprehensive literature search; status of publication (i.e., grey literature) was not used as an inclusion criteria |
| Gibbons et al., 2016 | RCTs, 2 *(2)*, 58 participants | VR-based interventions, 12^b^, 2 (n = 28) | Conventional therapy, 2 (n = 30) | Functional mobility for chronic stroke: WMD = -4.94 *ns* [-10.37 to 0.49] | 0.00 | Very low | Imprecision (- 2): number of participants within pooled analysis less than 100  Risk of bias (trial quality) (-1): 1 RCT at high risk of selection bias (sequence generation); 1 RCTs at high risk of detection bias;  Risk of bias (review quality) (-2): no comprehensive literature search; status of publication (i.e., grey literature) was not used as an inclusion criteria |
| Gibbons et al., 2016 | RCTs, 2 *(2)*, 58 participants | VR-based interventions with conventional therapy, 2 (n =28) | Conventional therapy, 2 (n = 30) | Functional balance for chronic stroke: SMD = 0.38 *ns* [-0.73 to 1.50] | 77.00 | Very low | Imprecision (- 2): number of participants within pooled analysis less than 100  Risk of bias (trial quality) (-1): 1 RCT at high risk of selection bias (sequence generation); 1 RCTs at high risk of detection bias;  Risk of bias (review quality) (-2): no comprehensive literature search; status of publication (i.e., grey literature) was not used as an inclusion criteria |
| Laver et al., 2017 | RCTs, 9 *(9)*, 366 participants | VR-based interventions alone, 9 (n = 182) | Conventional therapy, 9 (n = 184) | Upper limb function: SMD = 0.11 *ns* [-0.10 to 0.32] | 0.00 | High | No downgrade |

*Note.* *** *p* < .001, ** *p* < .01, * *p* < .05, *ns*- non-significant results. ^a^ = number of total number of participants could not be extracted; ; ^b^ = we were not able to identify whether VR-based interventions were delivered alone or in combination with conventional therapy; ^c^ = number of participants in the intervention/control group could not be extracted; Cohen’s *d* = Cohen’s *d* coefficient, effect size; MD = Mean difference, effect size; n = sample size; RCT = randomized controlled trial; SMD = Standardized Mean Difference, effect size; WMD = Weighted mean difference, effect size

Supplementary Table S12

*Subgroup comparisons.* *Moderator effects for comparisons between VR interventions alone versus VR interventions with conventional therapy*

| Author(s), year | Design, number of studies, total number of participants per comparison | Intervention, number of studies and participants per comparison | Control, number of studies and participants per comparison | Outcomes and effect measure (95% CI) | Heterogeneity (I^2^) |
| --- | --- | --- | --- | --- | --- |
| Corbetta et al., 2015 | RCTs, 7, 138 participants | VR-based interventions alone, 7 (total n = 65) | Conventional therapy, 7 (n = 73) | Walking speed: MD = 0.15** [0.10 to 0.19] in favor of VR | 26.00 |
| Corbetta et al., 2015 | RCTs, 1, 24 participants | VR-based interventions with conventional therapy, 1 (n = 12) | Conventional therapy, 1 (n = 12) | Walking speed: MD = 0.21 *ns* [–0.23 to 0.65] | n/a |
| Corbetta et al., 2015 | RCTs, 5, 114 participants | VR-based interventions alone (n = 53) | Conventional therapy, 5 (n = 61) | Mobility (Timed Up and Go): MD = 2.32** [1.22 to 3.42] in favor of VR | 84.00 |
| Corbetta et al., 2015 | RCTs, 2, 42 participants | VR-based interventions with conventional therapy, 2 (n = 21) | Conventional therapy, 2 (n = 21) | Mobility (Timed Up and Go): MD = 0.74** [0.42 to 1.17] in favor of VR | 0.00 |
| De Rooij et al., 2016 | RCTs, 8, 214 participants | VR-based interventions alone, 8 (n = 110) | Conventional therapy, 8 (n = 104) | Gait speed: SMD = 1.03** [0.38 to 1.69] in favor of VR | 78.00 |
| De Rooij et al., 2016 | RCTs, 1, 24 participants | VR-based interventions with conventional therapy, 1 (n = 12) | Conventional therapy, 1 (n = 12) | Gait speed: MD = 13.07** [5.76 to 20.38] in favor of VR | n/a |
| De Rooij et al., 2016 | RCTs, 6, 132 participants | VR-based interventions alone, 6 (n = 66) | Conventional therapy, 6 (n = 66) | Functional mobility (Timed Up and Go): MD = 2.48** [1.28 to 3.67] in favor of VR | 85.00 |
| De Rooij et al., 2016 | RCTs, 1, 20 participants | VR-based interventions with conventional therapy, 1 (n = 10) | Conventional therapy, 1 (n = 10) | Functional mobility (Timed Up and Go): MD = 0.70** [0.29 to 1.11] in favor of VR | n/a |
| Laver et al., 2017 | RCT, 3, 89 participants | VR-based interventions alone, 3 (n = 44) | Conventional therapy, 3 (n = 45) | Lower limb activity (Timed Up and Go): MD = -1.76 *ns* [-4.67 to 1.16] | 59.00 |
| Laver et al., 2017 | RCTs, 3, 93 participants | VR-based interventions with conventional therapy, 3 (n = 46) | Conventional therapy, 3 (n = 47) | Lower limb activity (Timed Up and Go): MD = -4.76** [-8.91 to -0.61] in favor of VR | 50.00 |
| Laver et al., 2017 | RCTs, 6, 139 participants | VR-based interventions alone, 6 (n = 70) | Conventional therapy, 6 (n = 69) | Lower limb activity, Gait speed: MD = 0.09 *ns* [-0.04 to 0.22] | 10.00 |
| Laver et al., 2017 | RCTs, 3, 57 participants | VR-based interventions with conventional therapy, 2 (n = 28) | Conventional therapy, 3 (n = 29) | Lower limb activity, Gait speed: MD = 0.08 *ns* [-0.05 to 0.21] | 0.00 |
| De Rooij et al., 2016 | RCTs, 5, 130 participants | VR-based interventions alone, 5 (n = 67) | Conventional therapy, 5 (n = 63) | Balance (Berg Balance Scale): MD = 2.18** [1.52 to 2.85] in favor of VR | 9.00 |
| De Rooij et al., 2016 | RCTs, 2, 44 participants | VR-based interventions with conventional therapy, 2 (n = 22) | Conventional therapy, 2 (n = 22) | Balance (Berg Balance Scale): MD = 1.17 *ns* [-6.54 to 8.88] | 98.00 |
| Laver et al., 2017 | RCT, 3, 72 participants | VR-based interventions alone, 3 (n = 35) | Conventional therapy, 3 (n = 37) | Balance: SMD = 0.39 *ns* [-0.09 to 0.86] | 10.00 |
| Laver et al., 2017 | RCTs, 7, 173 participants | VR-based interventions with conventional therapy, 7 (n = 87) | Conventional therapy, 7 (n = 86) | Balance: SMD = 0.59** [0.28 to 0.90] in favor of VR | 32.00 |
| Laver et al., 2017 | RCTs, 22, quasi-RCTs, 1, 1038 participants | VR-based interventions alone, 23 (n = 533) | Conventional therapy, 23 (n = 505) | Upper limb function (composite measure): SMD = 0.07 *ns* [-0.05 to 0.20] | 43.00 |
| Laver et al., 2017 | RCTs, 11, 210 participants | VR-based interventions with conventional therapy, 11 (n = 110) | Conventional therapy, 9 (n = 87); No intervention, 1 (n = 5); Control group type not specified, 1 (n = 8) | Upper limb function (composite measure): SMD = 0.49** [0.21 to 0.77] in favor of VR | 0.00 |
| Laver et al., 2017 | RCTs, 10, 466 participants | VR-based interventions alone, 10 (n = 245) | Conventional therapy, 10 (n = 221) | Activity limitation: SMD = 0.25** [0.06 to 0.43] in favor of VR | 22.00 |
| Laver et al., 2017 | RCTs, 8, 153 participants | VR-based interventions with conventional therapy, 8 (n = 80) | Conventional therapy, 7 (n = 66); No intervention, 1 (n = 7) | Activity limitation: SMD = 0.44** [0.11-0.76] in favour of VR | 0.00 |

*Note.* *** *p* < .001, ** *p* < .01, * *p* < .05, *ns*- non-significant results. MD = Mean difference, effect size; n = sample size; n/a = not applicable; RCT = randomized controlled trial; SMD = Standardized Mean Difference, effect size

Supplementary Table S13

*Subgroup comparisons.* *Moderator effects for comparisons between time dose matched interventions versus time non-dose matched interventions*

| Author(s), year | Design, number of studies, total number of participants per comparison | Intervention, number of studies and participants per comparison | Control, number of studies and participants per comparison | Outcomes and effect measure (95% CI) | Heterogeneity (I^2^) |
| --- | --- | --- | --- | --- | --- |
| Saywell et al., 2017 | RCTs, 4, 88 participants | VR-based interventions with conventional therapy, non-dose matched, 2 (n = 24); VR-based interventions alone, non-dose matched, 2 (n = 21) | Conventional therapy, 2 (n = 24); No intervention, 2 (n = 19) | Lower limb gait: SMD = 0.36 *ns* [-0.05 to 0.79] | 0.00 |
| Saywell et al., 2017 | RCTs, 6, 180 participants | VR-based interventions with conventional therapy, dose matched, 3 (n = 67); VR-based interventions alone, dose matched, 3 (n = 28) | Conventional therapy, 6 (n = 85) | Lower limb gait: SMD = 0.28 *ns* [-0.00 to 0.58] | 0.00 |
| Saywell et al., 2017 | RCTs, 3, 66 participants | VR-based interventions with conventional therapy, non- dose matched, 1 (n = 13); VR-based interventions alone, non-dose matched, 2 (n = 21) | Conventional therapy, 1 (n = 13); No intervention, 2 (n = 19) | Fugl Meyer: SMD = 0.29 *ns* [-0.19 to 0.78] | 0.00 |
| Saywell et al., 2017 | RCTs, 4, 162 participants | VR-based interventions with conventional therapy, dose matched, 1 (n = 20); VR-based interventions alone, dose matched, 3 (n = 63); | Conventional therapy, 4 (n = 79) | Fugl Meyer: SMD = 0.43^ss^ [0.12 to 0.74] | 0.00 |
| Saywell et al., 2017 | RCTs, 4, 88 participants | VR-based interventions with conventional therapy, non-dose matched, 3 (n = 39), VR-based interventions alone, non-dose matched, 1 (n = 5) | Conventional therapy, 3 (n = 39) No intervention, 1 (n = 5) | Upper limb: SMD = 0.42 *ns* [-0.00 to 0.84] | 0.00 |
| Saywell et al., 2017 | RCTs, 5, 180 participants | VR-based interventions with conventional therapy, dose matched, 1 (n = 20); VR-based interventions alone, dose matched, 4 (n = 72) | Conventional therapy, 5 (n = 88) | Upper limb: SMD = 0.22 *ns* [-0.07 to 0.53] | 14.10 |

*Note.* *** *p* < .001, ** *p* < .01, * *p* < .05, *ns*- non-significant results. n = sample size; RCT = randomized controlled trial; SMD = Standardized Mean Difference, effect size; ^ss^ = statistically significant results, without *p* value provided by authors

Supplementary Table S14

*Subgroup comparisons. Moderator effects for comparisons between commercially available systems versus customized systems*

| Author(s), year | Design, number of studies, total number of participants per comparison | Intervention, number of studies and participants per comparison | Control, number of studies and participants per comparison | Outcomes and effect measure (95% CI) | Heterogeneity (I^2^) |
| --- | --- | --- | --- | --- | --- |
| Aminov et al., 2018 | RCTs, 14^a^ | Commercially available VR systems, 14^b^ | Conventional therapy, 14^c^ | Motor, functional, and cognitive outcomes combined: Hedge’s *g* = 0.33** [0.14 to 0.51] in favor of VR | 0.00 |
| Aminov et al., 2018 | RCTs, 19^a^ | Customized VR systems, 19^b^ | Conventional therapy, 14^c^ | Motor, functional, and cognitive outcomes combined: Hedge’s *g* = 0.58** [0.41 to 0.76] in favor of VR | 0.00 |
| Chen et al., 2014 | RCTs, 4^a^ | Commercially available VR systems, 4^c^ | Conventional therapy, (NS) | Upper extremity: SMD = 0.31^ss^ | 0.00 |
| Chen et al., 2014 | RCTs, 10^a^ | Customized VR systems, 10^c^ | Conventional therapy, (NS) | Upper extremity: SMD = 1.27^ss^ | 55.00 |
| Chen et al., 2018 | RCTs^a,b^ | Commercially available VR systems^c,d^ | ^e,f,g^ | Upper extremity, ambulation and postural control: Cohen’s *d* = 0.62 *ns* | ^h^ |
| Chen et al., 2018 | RCTs^a,b^ | Customized VR systems^c,d^ | ^e,f,g^ | Upper extremity, ambulation and postural control: Cohen’s *d* = 1.57^ss^ | ^h^ |
| Chen et al., 2018 | RCTs^a,b^ | Commercially available VR systems^c,d^ | ^e,f,g^ | Arm function: Cohen’s *d* = 0.49 *ns* | ^h^ |
| Chen et al., 2018 | RCTs^a,b^ | Customized VR systems^c,d^ | ^e,f,g^ | Arm function: Cohen’s *d* = 2.16^ss^ | ^h^ |
| Chen et al., 2018 | RCTs^a,b^ | Commercially available VR systems^c,d^ | ^e,f,g^ | Postural control: *ns*, but further data not provided | ^h^ |
| Chen et al., 2018 | RCTs^a,b^ | Customized VR systems^c,d^ | ^e,f,g^ | Postural control: *ns*, but further data not provided | ^h^ |
| Maier et al., 2019 | RCTs, 19, 945 participants | Customized VR systems, 19 (n = 550) | Conventional therapy, 18 (n = 390); Passive control group, 1 (n = 5) | Upper limb body function: SMD = 0.23** [0.10 to 0.36] in favour if VR | 0.00 |
| Maier et al., 2019 | RCTs, 7, 310 participants | Commercially available VR systems, 7 (n = 154) | Conventional therapy, 7 (n = 156) | Upper limb body function: SMD = 0.16 *ns* [-0.14 to 0.47] | 37.00 |
| Maier et al., 2019 | RCTs, 23, 1103 participants | Customized VR systems, 23 (n = 632) | Conventional therapy, 21 (n = 457); Passive control group, 2 (n = 14) | Upper limb activity: SMD = 0.31** [0.15 to 0.47] in favour if VR | 29.00 |
| Maier et al., 2019 | RCTs, 7, 310 participants | Commercially available VR systems, 7 (n = 152) | Conventional therapy, 7 (n = 152) | Upper limb activity: SMD = 0.15 *ns* [0.13 to 0.41] in favour if VR | 32.00 |
| Laver et al., 2017 | RCTs, 7, 507 participants | Commercially available VR Systems, VR intervention delivered alone, 7 (n = 267) | Conventional therapy, 7 (n = 240) | Upper limb function: SMD = -0.20 *ns* [-0.15 to 0.45] | 66.00 |
| Laver et al., 2017 | RCTs, 15, Quasi-RCTs, 1, 531 participants | Customized VR systems, VR intervention delivered alone, 16 (n = 266) | Conventional therapy, 7 (n = 265) | Upper limb function: SMD = 0.17 *ns* [0.00 to 0.35] | 17.00 |
| Laver et al., 2017 | RCTs, 3, 71 participants | Commercially available VR Systems, VR intervention delivered with conventional therapy, (n = 36) | Conventional therapy, 2 (n = 27); Control group^e^, 1 (n = 8) | Upper limb function: SMD = 0.67** [0.18 to 1.15] in favor of commercially available VR Systems | 32.00 |
| Laver et al., 2017 | RCTs, 8, 139 participants | Customized VR systems, VR intervention delivered with conventional therapy, 8 (n = 74) | Conventional therapy, 6 (n = 46); No intervention, 2 (n = 19) | Upper limb function: SMD = 0.40* [0.06 to 0.75] in favor of customized VR systems | 0.00 |
| Lohse et al., 2014 | RCTs, 3, 58 participants | IG-based interventions, 3 (n = 29) | Conventional therapy, 3 (n = 29) | Body function: Hedges’ *g* = 0.76 *ns* [-0.17 to 1.70] | 66.30 |
| Lohse et al., 2014 | RCTs, 13, 401 participants | VE-based interventions, 13 (n = 211) | Conventional therapy, 13 (n = 190) | Body function: Hedges’ *g* = 0.43*** [0.22 to 0.64] in favor of VR | 11.03 |
| Lohse et al., 2014 | RCTs, 4, 75 participants | IG-based interventions, 4 (n = 38) | Conventional therapy, 4 (n = 37) | Activity: Hedges’ *g* = 0.76 *ns* [-0.25 to 1.76] | 77.17 |
| Lohse et al., 2014 | RCTs, 18, 479 participants | VE-based interventions, 18 (n = 245) | Conventional therapy, 18 (n = 234) | Activity: Hedges’ *g* = 0.54*** [0.28 to 0.81] in favor of VR | 49.18 |

*Note.* *** *p* < .001, ** *p* < .01, * *p* < .05, *ns*- non-significant results. ^a^ = number of total number of participants could not be extracted; ^b^ = number of primary studies included could not be extracted; ^c^ = number of participants in the intervention group could not be extracted; ^d^ = number of studies in the intervention group could not be extracted; ^e^ = control group type not specified; ^f^ = number of participants in the control group could not be extracted; ^g^ = number of studies in the control group could not be extracted; ^h^ = I^2^ value not reported; Cohen’s *d* = Cohen’s *d* coefficient, effect size; n = sample size; RCT = randomized controlled trial; SMD = Standardized Mean Difference, effect size; ^ss^ = statistically significant results, without *p* value provided by authors

Supplementary Table S15

*Subgroup comparisons.* *Moderator effects for comparisons between VE-based interventions versus IG-based interventions*

| Author(s), year | Design, number of studies, total number of participants per comparison | Intervention, number of studies and participants per comparison | Control, number of studies and participants per comparison | Outcomes and effect measure (95% CI) | Heterogeneity (I^2^) |
| --- | --- | --- | --- | --- | --- |
| Iruthayarajah et al., 2017 | RCTs, 5, 127 participants | IG-based interventions, 5 (n = 63) | Conventional therapy, 5 (n = 64) | Functional Mobility (Timed Up and Go Test): SMD = 0.20 *ns* [-0.14 to 0.55] | 0.00 |
| Iruthayarajah et al., 2017 | RCTs, 5, 113, participants | VE-based interventions (treadmill training), 5 (n = 52) | Conventional therapy, 5 (n = 61) | Functional Mobility (Timed Up and Go Test): SMD = 0.43** [0.04 to 0.82] in favor of VR | 0.00 |
| Iruthayarajah et al., 2017 | RCTs, 3, 61 participants | VE-based interventions (postural training), 3 (n = 30) | Conventional therapy, 3 (n = 31) | Functional Mobility (Timed Up and Go Test): SMD = 0.60* [0.08 to 1.13] in favor of VR | 66.56 |
| Iruthayarajah et al., 2017 | RCTs, 4, 87 participants | IG-based interventions, 4 (n = 44) | Conventional therapy, 3 (n = 29); No intervention, 1 (n = 14) | Balance (Berg Balance Scale): SMD = 0.33 *ns* [-0.08 to 0.76] | 0.00 |
| Iruthayarajah et al., 2017 | RCTs, 3, 72 participants | VE-based interventions (treadmill training), 3 (n = 31) | Conventional therapy, 3 (n = 41) | Balance (Berg Balance Scale): SMD = 0.39 *ns* [-0.10 to 0.89] | 0.00 |
| Iruthayarajah et al., 2017 | RCTs, 5, 115 participants | VE-based interventions (postural training), 5 (n = 57) | Conventional therapy, 5 (n = 58) | Balance (Berg Balance Scale): SMD = 0.70**[0.32 to 1.09] | 52.53 |

*Note.* *** *p* < .001, ** *p* < .01, * *p* < .05, *ns*- non-significant results. ^a^ = the total number of participants could not be extracted; ^b^ = number of participants in the intervention group could not be extracted; ^c^ = number of participants from the control group could not be extracted; IG = Interactive gaming; Hedge’s *g* = Hedge’s *g* coefficient, effect size; n = sample size; RCT = randomized controlled trial; SMD = Standardized Mean Difference, effect size; VE = Virtual environment

**References to studies excluded from this review**

Agostini, M., Moja, L., Banzi, R., Pistotti, V., Tonin, P., Venneri, A., & Turolla, A. (2015). Telerehabilitation and recovery of motor function: a systematic review and meta-analysis. *Journal of Telemedicine and Telecare, 21*(4), 202-213. doi:10.1177/1357633x15572201

Alvarez-Bueno, C., Pesce, C., Cavero-Redondo, I., Sanchez-Lopez, M., Martinez-Hortelano, J. A., & Martinez-Vizcaino, V. (2017). The effect of physical activity interventions on children's cognition and metacognition: a systematic review and meta-analysis. *Journal of the American Academy of Child and Adolescent Psychiatry, 56*(9), 729-738. doi:10.1016/j.jaac.2017.06.012

Alwardat, M., Etoom, M., al Dajah, S., Schirinzi, T., di Lazzaro, G., Sinibaldi Salimei, P., . . . Pisani, A. (2018). Effectiveness of robot-assisted gait training on motor impairments in people with Parkinson's disease: a systematic review and meta-analysis. *International Journal of Rehabilitation Research, 41*(4), 287-296. doi:10.1097/MRR.0000000000000312

Araujo, P. A., Starling, J. M. P., Oliveira, V. C., Gontijo, A. P. B., & Mancini, M. C. (2019). Combining balance-training interventions with other active interventions may enhance effects on postural control in children and adolescents with cerebral palsy: a systematic review and meta-analysis [with consumer summary]. *Brazilian Journal of Physical Therapy :Epub ahead of print*.

Balasukumaran, T., Olivier, B., & Ntsiea, M. V. (2019). The effectiveness of backward walking as a treatment for people with gait impairments: a systematic review and meta-analysis. *Clinical rehabilitation*, *33*(2), 171-182. doi:10.1177/0269215518801430

Bania, T., Chiu, H.-C., & Billis, E. (2019). Activity training on the ground in children with cerebral palsy: Systematic review and meta-analysis. *Physiotherapy Theory and Practice, 35*(9), 810-821.

Bediou, B., Adams, D. M., Mayer, R. E., Tipton, E., Green, C. S., & Bavelier, D. (2018). Meta-analysis of action video game impact on perceptual, attentional, and cognitive skills. *Psychological Bulletin, 144*(1), 77-110. doi:10.1037/bul0000130

Booth, A. T. C., Buizer, A. I., Meyns, P., Lansink, I., Steenbrink, F., & van der Krogt, M. M. (2018). The efficacy of functional gait training in children and young adults with cerebral palsy: a systematic review and meta-analysis. *Developmental Medicine and Child Neurology, 60*(9), 866. doi:10.1111/dmcn.13708

Buchignani, B., Beani, E., Pomeroy, V., Iacono, O., Sicola, E., Perazza, S., . . . Sgandurra, G. (2019). Action observation training for rehabilitation in brain injuries: a systematic review and meta-analysis. *BMC Neurology, 19(344):Epub*.

Burton, E., Farrier, K., Galvin, R., Johnson, S., Horgan, N. F., Warters, A., & Hill, K. D. (2019). Physical activity programs for older people in the community receiving home care services: systematic review and meta-analysis. *Clinical Interventions in Aging, 14:1045-1064*.

Busk, H., Stausholm, M. B., Lykke, L., & Wienecke, T. (2019). Electrical stimulation in lower limb during exercise to improve gait speed and functional motor ability 6 months poststroke. A review with meta-analysis. *Journal of Stroke & Cerebrovascular Diseases, Epub ahead of print*.

Cabrales, S. X., Jr. (2018). *Exploring the effectiveness of virtual reality therapy compared to mirror therapy in treating individuals with upper extremity dysfunction following stroke: A meta analysis.* ProQuest Information & Learning, US. PsycINFO database.

Cacciata, M., Stromberg, A., Lee, J.-A., Sorkin, D., Lombardo, D., Clancy, S., . . . Evangelista, L. S. (2019). Effect of exergaming on health-related quality of life in older adults: A systematic review. *International Journal of Nursing Studies, 93*, 30-40. doi:10.1016/j.ijnurstu.2019.01.010

Cao, P. Y., Zhao, Q. H., Xiao, M. Z., Kong, L. N., & Xiao, L. (2018). The effectiveness of exercise for fall prevention in nursing home residents: a systematic review meta-analysis. *Journal of Advanced Nursing, 74*(11), 2511-2522. doi:10.1111/jan.13814

Carvalho, I., Pinto, S. M., Chagas, D. V., Praxedes dos Santos, J. L., de Sousa Oliveira, T., & Batista, L. A. (2017). Robotic gait training for individuals with cerebral palsy: a systematic review and meta-analysis. *Archives of Physical Medicine and Rehabilitation, 98*(11), 2332-2344. doi:10.1016/j.apmr.2017.06.018

Chen, Y. P., Pope, S., Tyler, D., & Warren, G. L. (2014). Effectiveness of constraint-induced movement therapy on upper-extremity function in children with cerebral palsy: a systematic review and meta-analysis of randomized controlled trials. *Clinical Rehabilitation, 28*(10), 939-953. doi:10.1177/0269215514544982

Choi, M., & Hector, M. (2012). Effectiveness of intervention programs in preventing falls: a systematic review of recent 10 years and meta-analysis. *Journal of the American Medical Directors Association, 13*(2), 188.e113-188.e121. doi:10.1016/j.jamda.2011.04.022

Chung, C. L. H., Thilarajah, S., & Tan, D. (2016). Effectiveness of resistance training on muscle strength and physical function in people with Parkinson's disease: a systematic review and meta-analysis. *Clinical Rehabilitation, 30*(1), 11-23. doi:10.1177/0269215515570381

Cooke, E. V., Mares, K., Clark, A., Tallis, R. C., & Pomeroy, V. M. (2010). The effects of increased dose of exercise-based therapies to enhance motor recovery after stroke: a systematic review and meta-analysis. *BMC Medicine, 8*(60). doi:10.1186/1741-7015-8-60

Corbetta, D., Sirtori, V., Moja, L., & Gatti, R. (2010). Constraint-induced movement therapy in stroke patients: systematic review and meta-analysis. *European Journal of Physical and Rehabilitation Medicine, 46*(4), 537-544.

Corregidor-Sánchez, A. I., Segura-Fragoso, A., Rodríguez-Hernández, M., Criado-Alvarez, J. J., González-Gonzalez, J., & Polonio-López, B. (2020). Can exergames contribute to improving walking capacity in older adults? A systematic review and meta-analysis. *Maturitas*, *132*, 40-48. doi:10.1016/j.maturitas.2019.12.006

Cottrell, M. A., Galea, O. A., O'Leary, S. P., Hill, A. J., & Russell, T. G. (2017). Real-time telerehabilitation for the treatment of musculoskeletal conditions is effective and comparable to standard practice: a systematic review and meta-analysis. *Clinical Rehabilitation, 31*(5), 625-638. doi:10.1177/0269215516645148

Coupar, F. M. (2012). *Exploring upper limb interventions after stroke.* (Ph.D.). University of Glasgow (United Kingdom), Ann Arbor. Retrieved from https://search.proquest.com/docview/1442474431?accountid=11862

Coussement, J., de Paepe, L., Schwendimann, R., Denhaerynck, K., Dejaeger, E., & Milisen, K. (2008). Interventions for preventing falls in acute- and chronic-care hospitals: a systematic review and meta-analysis. *Journal of the American Geriatrics Society, 56*(1), 29-36. doi:10.1111/j.1532-5415.2007.01508.x

Crocker, T., Young, J., Forster, A., Brown, L., Ozer, S., & Greenwood, D. C. (2013). The effect of physical rehabilitation on activities of daily living in older residents of long-term care facilities: systematic review with meta-analysis. *Age and Ageing, 42*(6), 682-688. doi:10.1093/ageing/aft133

Cruickshank, T. M., Reyes, A. R., & Ziman, M. R. (2015). A systematic review and meta-analysis of strength training in individuals with multiple sclerosis or Parkinson disease. *Medicine, 94*(4), e411. doi:10.1097/MD.0000000000000411

Da Rocha, P. A., McClelland, J., & Morris, M. (2015). Alternative physical therapies for movement disorders in parkinson's disease: A systematic review. *Movement Disorders, 30*, S114. doi:10.1002/mds.26295

Da Rocha, P. A., McClelland, J., & Morris, M. E. (2015). Complementary physical therapies for movement disorders in Parkinson's disease: a systematic review. *European Journal of Physical and Rehabilitation Medicine, 51*(6), 693-704.

Dillon, L., Clemson, L., Ramulu, P., Sherrington, C., & Keay, L. (2018). A systematic review and meta-analysis of exercise-based falls prevention strategies in adults aged 50+ years with visual impairment. *Ophthalmic & Physiological Optics, 38*(4), 456-467. doi:10.1111/opo.12562

Dockx, K., Bekkers, E. M. J., Van den Bergh, V., Ginis, P., Rochester, L., Hausdorff, J. M., . . . Nieuwboer, A. (2016). Virtual reality for rehabilitation in Parkinson's disease. *Cochrane Database of Systematic Reviews* (12). doi:10.1002/14651858.CD010760.pub2

Elbanna, S. T., Elshennawy, S., & Ayad, M. N. (2019). Noninvasive Brain Stimulation for Rehabilitation of Pediatric Motor Disorders Following Brain Injury: Systematic Review of Randomized Controlled Trials. *Archives of Physical Medicine and Rehabilitation, 100*(10), 1945-1963. doi:10.1016/j.apmr.2019.04.009

Elsner, B., Kugler, J., Pohl, M., & Mehrholz, J. (2016). Transcranial direct current stimulation (tDCS) for improving activities of daily living, and physical and cognitive functioning, in people after stroke. *Cochrane Database of Systematic Reviews* (3), 190. doi:10.1002/14651858.CD009645.pub3

Falck, R. S., Davis, J. C., Best, J. R., Crockett, R. A., & Liu-Ambrose, T. (2019). Impact of exercise training on physical and cognitive function among older adults: a systematic review and meta-analysis [with consumer summary]. *Neurobiology of Aging, 7,* 119-130.

Fedewa, M. V., Hathaway, E. D., Williams, T. D., & Schmidt, M. D. (2017). Effect of exercise training on non-exercise physical activity: a systematic review and meta-analysis of randomized controlled trials. *Sports Medicine, 47*(6), 1171-1182. doi:10.1007/s40279-016-0649-z

Finnegan, S., Seers, K., & Bruce, J. (2019). Long-term follow-up of exercise interventions aimed at preventing falls in older people living in the community: a systematic review and meta-analysis [with consumer summary]. *Physiotherapy, 105*(2), 187-199.

French, B., Thomas, L., Leathley, M., Sutton, C., McAdam, J., Forster, A., . . . Watkins, C. (2010). Does repetitive task training improve functional activity after stroke? A Cochrane systematic review and meta-analysis. *Journal of Rehabilitation Medicine, 42*(1), 9-14. doi:10.2340/16501977-0473

Goodwin, V. A., Richards, S. H., Taylor, R. S., Taylor, A. H., & Campbell, J. L. (2008). The effectiveness of exercise interventions for people with Parkinson's disease: a systematic review and meta-analysis. *Movement Disorders, 23*(5), 631-640. doi:10.1002/mds.21922

Grande, G. D., Oliveira, C. B., Morelhao, P. K., Sherrington, C., Tiedemann, A., Pinto, R. Z., & Franco, M. R. (2019). Interventions promoting physical activity among older adults: a systematic review and meta-analysis. *The Gerontologist, Epub ahead of print*.

Hamilton, A., Wakely, L., & Marquez, J. (2018). Transcranial Direct-Current Stimulation on Motor Function in Pediatric Cerebral Palsy: A Systematic Review. *Pediatric Physical Therapy, 30*(4), 291-301. doi:10.1097/pep.0000000000000535

Hanratty, C. E., McVeigh, J. G., Kerr, D. P., Basford, J. R., Finch, M. B., Pendleton, A., & Sim, J. (2012). The effectiveness of physiotherapy exercises in subacromial impingement syndrome: a systematic review and meta-analysis. *Seminars in Arthritis and Rheumatism, 42*(3), 297-316. doi:10.1016/j.semarthrit.2012.03.015

Harris, D. M., Rantalainen, T., Muthalib, M., Johnson, L., & Teo, W.-P. (2015). Exergaming as a viable therapeutic tool to improve static and dynamic balance among older adults and people with idiopathic Parkinson’s disease: A systematic review and meta-analysis. *Frontiers in Aging Neuroscience, 7*, 167. doi:10.3389/fnagi.2015.00167

Hart, P. D., & Buck, D. J. (2019). The effect of resistance training on health-related quality of life in older adults: systematic review and meta-analysis. *Health Promotion Perspectives, 9*(1), 1-12.

Hayward, K. S., Barker, R. N., Carson, R. G., & Brauer, S. G. (2014). The effect of altering a single component of a rehabilitation programme on the functional recovery of stroke patients: a systematic review and meta-analysis. *Clinical Rehabilitation, 28*(2), 107-117. doi:10.1177/0269215513497601

Hillier, S., & McDonnell, M. (2016). Is vestibular rehabilitation effective in improving dizziness and function after unilateral peripheral vestibular hypofunction? An abridged version of a Cochrane Review. *European Journal of Physical and Rehabilitation Medicine, 52*(4), 541-556.

Hopewell, S., Copsey, B., Nicolson, P., Adedire, B., Boniface, G., & Lamb, S. (2019). Multifactorial interventions for preventing falls in older people living in the community: a systematic review and meta-analysis of 41 trials and almost 20,000 participants [with consumer summary]. *British Journal of Sports Medicine*.

Howard, M. C. (2017). A meta-analysis and systematic literature review of virtual reality rehabilitation programs. *Computers in Human Behavior, 70*, 317-327. doi:10.1016/j.chb.2017.01.013

Hugues, A., di Marco, J., Ribault, S., Ardaillon, H., Janiaud, P., Xue, Y., . . . Rode, G. (2019). Limited evidence of physical therapy on balance after stroke: a systematic review and meta-analysis. *PLoS ONE, 14(8):e0221700*.

Isabel Corregidor-Sanchez, A., Segura-Fragoso, A., Rodriguez-Hernandez, M., Jose Criado-Alvarez, J., Gonzalez-Gonzalez, J., & Polonio-Lopez, B. (2020). Can exergames contribute to improving walking capacity in older adults? A systematic review and meta-analysis. *Maturitas, 132*, 40-48. doi:10.1016/j.maturitas.2019.12.006

Kalron, A., & Zeilig, G. (2015). Efficacy of exercise intervention programs on cognition in people suffering from multiple sclerosis, stroke and Parkinson's disease: A systematic review and meta-analysis of current evidence. *Neurorehabilitation, 37*(2), 273-289. doi:10.3233/nre-151260

Kayambu, G., Boots, R., & Paratz, J. (2013). Physical therapy for the critically ill in the ICU: a systematic review and meta-analysis. *Critical Care Medicine, 41*(6), 1543-1554. doi:10.1097/CCM.0b013e31827ca637

Lal, A., Kolakowsky-Hayner, S. A., Ghajar, J., & Balamane, M. (2018). The effect of physical exercise after a concussion: a systematic review and meta-analysis. *The American Journal of Sports Medicine, 46*(3), 743-752. doi:10.1177/0363546517706137

Lau, H. M., Smit, J. H., Fleming, T. M., & Riper, H. (2017). Serious games for mental health: Are they accessible, feasible, and effective? A systematic review and meta-analysis. *Frontiers in Psychiatry, 7*. doi:10.3389/fpsyt.2016.00209

Logan, P. A., Gladman, J. R. F., Drummond, A. E. R., Radford, K. A., & Grp, T. S. (2003). A study of interventions and related outcomes in a randomized controlled trial of occupational therapy and leisure therapy for community stroke patients. *Clinical Rehabilitation, 17*(3), 249-255. doi:10.1191/0269215503cr593oa

Fernández López, R., & Antolí, A. (2020). Computer-based cognitive interventions in acquired brain injury: A systematic review and meta-analysis of randomized controlled trials. *PLOS ONE*, *15*(7), e0235510.

Louie, D. R., Lim, S. B., & Eng, J. J. (2019). The efficacy of lower extremity mirror therapy for improving balance, gait, and motor function poststroke: a systematic review and meta-analysis. *Journal of Stroke and Cerebrovascular Diseases*, *28*(1), 107-120. doi:10.1016/j.jstrokecerebrovasdis.2018.09.017

Lucas, B. R., Elliott, E. J., Coggan, S., Pinto, R. Z., Jirikowic, T., McCoy, S. W., & Latimer, J. (2016). Interventions to improve gross motor performance in children with neurodevelopmental disorders: A meta-analysis. *BMC Pediatrics, 16*(1). doi:10.1186/s12887-016-0731-6

Lundell, S., Holmner, A., Rehn, B., Nyberg, A., & Wadell, K. (2015). Telehealthcare in COPD: a systematic review and meta-analysis on physical outcomes and dyspnea. *Respiratory Medicine, 109*(1), 11-26. doi:10.1016/j.rmed.2014.10.008

Mansfield, A., Wong, J. S., Bryce, J., Knorr, S., & Patterson, K. K. (2015). Does perturbation-based balance training prevent falls? Systematic review and meta-analysis of preliminary randomized controlled trials. *Physical Therapy, 95*(5), 700-709. doi:10.2522/ptj.20140090

Mansor, N. S., Chow, C. M., & Halaki, M. (2019). Cognitive effects of video games in older adults and their moderators: a systematic review with meta-analysis and meta-regression. *Aging & Mental Health*, 1-16. doi:10.1080/13607863.2019.1574710

Martins, E., Cordovil, R., Oliveira, R., Letras, S., Lourenco, S., Pereira, I., . . . Marques, M. (2016). Efficacy of suit therapy on functioning in children and adolescents with cerebral palsy: a systematic review and meta-analysis. *Developmental Medicine and Child Neurology, 58*(4), 348-360. doi:10.1111/dmcn.12988

McIntyre, A., Viana, R., Janzen, S., Mehta, S., Pereira, S., & Teasell, R. (2012). Systematic review and meta-analysis of constraint-induced movement therapy in the hemiparetic upper extremity more than six months post stroke. *Topics in Stroke Rehabilitation, 19*(6), 499-513. doi:10.1310/tsr1906-499

Mentiplay, B. F., FitzGerald, T. L., Clark, R. A., Bower, K. J., Denehy, L., & Spittle, A. J. (2019). Do video game interventions improve motor outcomes in children with developmental coordination disorder? A systematic review using the ICF framework. *BMC Pediatrics, 19*. doi:10.1186/s12887-018-1381-7

Metcalf, B., Henley, W., & Wilkin, T. (2012). Effectiveness of intervention on physical activity of children: systematic review and meta-analysis of controlled trials with objectively measured outcomes (EarlyBird 54). *BMJ, 27*, 345, e5888. doi: 10.1136/bmj.e5888

Moreau, N. G., Bodkin, A. W., Bjornson, K., Hobbs, A., Soileau, M., & Lahasky, K. (2016). Effectiveness of rehabilitation interventions to improve gait speed in children with cerebral palsy: systematic review and meta-analysis. *Physical Therapy, 96*(12), 1938-1954. doi:10.2522/ptj.20150401

Morello, R. T., Soh, S. E., Behm, K., Egan, A., Ayton, D., Hill, K., . . . Barker, A. L. (2019). Multifactorial falls prevention programmes for older adults presenting to the emergency department with a fall: systematic review and meta-analysis [with consumer summary]. *Injury Prevention, 25*(6), 557-564.

Morgan, C., Novak, I., & Badawi, N. (2013). Enriched environments and motor outcomes in cerebral palsy: systematic review and meta-analysis. *Pediatrics, 132*(3), e735-e746. doi:10.1542/peds.2012-3985

Morgan, P. J., Barnett, L. M., Cliff, D. P., Okely, A. D., Scott, H. A., Cohen, K. E., & Lubans, D. R. (2013). Fundamental movement skill interventions in youth: a systematic review and meta-analysis. *Pediatrics, 132*(5), e1361-e1383. doi:10.1542/peds.2013-1167

Mura, G., Carta, M. G., Sancassiani, F., Machado, S., & Prosperini, L. (2018). Active exergames to improve cognitive functioning in neurological disabilities: a systematic review and meta-analysis. *European Journal of Physical and Rehabilitation Medicine, 54*(3), 450-462. doi:10.23736/s1973-9087.17.04680-9

Murphy, S., & Tickle-Degnen, L. (2001). The effectiveness of occupational therapy-related treatments for persons with Parkinson's disease: a meta-analytic review. *The American Journal of Occupational Therapy, 55*(4), 385-392.

Ng, Y.-L., Ma, F., Ho, F. K., Ip, P., & Fu, K.-w. (2019). Effectiveness of virtual and augmented reality-enhanced exercise on physical activity, psychological outcomes, and physical performance: A systematic review and meta-analysis of randomized controlled trials. *Computers in Human Behavior, 99*, 278-291. doi:10.1016/j.chb.2019.05.026

Ni, M., Hazzard, J. B., Signorile, J. F., & Luca, C. (2018). Exercise guidelines for gait function in Parkinson's disease: a systematic review and meta-analysis. *Neurorehabilitation and Neural Repair, 32*(10), 872-886. doi: 10.1177/1545968318801558

Norouzi-Gheidari, N., Archambault, P. S., & Fung, J. (2012). Effects of robot-assisted therapy on stroke rehabilitation in upper limbs: systematic review and meta-analysis of the literature. *Journal of Rehabilitation Research and Development, 49*(4), 479-496. 6360. doi:10.1682/JRRD.2010.10.0210

Pacheco, T. B. F., de Medeiros, C. S. P., de Oliveira, V. H. B., Vieira, E. R., & da Costa Cavalcanti, F. A. (2020). Effectiveness of exergames for improving mobility and balance in older adults: A systematic review and meta-analysis. *Systematic Reviews, 9,* 163. doi:10.1186/s13643-020-01421-7

Pearsall, R., Smith, D. J., Pelosi, A., & Geddes, J. (2014). Exercise therapy in adults with serious mental illness: a systematic review and meta-analysis. *BMC Psychiatry,14*(117). doi:10.1186/1471-244X-14-117

Perrochon, A., Borel, B., Istrate, D., Compagnat, M., & Daviet, J.-C. (2019). Exercise-based games interventions at home in individuals with a neurological disease: A systematic review and meta-analysis. *Annals of Physical and Rehabilitation Medicine, 62*(5), 366-378. doi:10.1016/j.rehab.2019.04.004

Pogrebnoy, D., & Dennett, A. (2020). Exercise programs delivered according to guidelines improve mobility in people with stroke: a systematic review and meta-analysis. *Archives of Physical Medicine and Rehabilitation, 101*(1), 154-165.

Pope, Z., Zeng, N., & Gao, Z. (2017). The effects of active video games on patients' rehabilitative outcomes: A meta-analysis. *Preventive Medicine, 95*(1), 38-46. doi:10.1016/j.ypmed.2016.12.003

Reedman, S., Boyd, R. N., & Sakzewski, L. (2017). The efficacy of interventions to increase physical activity participation of children with cerebral palsy: a systematic review and meta-analysis. *Developmental Medicine and Child Neurology, 59*(10), 1011-1018. doi:10.1111/dmcn.13413

Rintala, A., Paivarinne, V., Hakala, S., Paltamaa, J., Heinonen, A., Karvanen, J., & Sjogren, T. (2019). Effectiveness of Technology-Based Distance Physical Rehabilitation Interventions for Improving Physical Functioning in Stroke: A Systematic Review and Meta-analysis of Randomized Controlled Trials. *Archives of Physical Medicine and Rehabilitation, 100*(7), 1339-1358. doi:10.1016/j.apmr.2018.11.007

Rosa, P. J., Sousa, C., Faustino, B., Feiteira, F., Oliveira, J., Lopes, P., ... & Morais, D. (2016, October). The effect of virtual reality-based serious games in cognitive interventions: a meta-analysis study. In *Proceedings of the 4th Workshop on ICTs for improving Patients Rehabilitation Research Techniques* (pp. 113-116). ACM. doi:10.1145/3051488.3051510

Rosenbaum, S., Tiedemann, A., & Ward, P. B. (2014). Meta-analysis physical activity interventions for people with mental illness: a systematic review and meta-analysis. *The Journal of Clinical Psychiatry*, *75*(0), 1-11. doi:10.4088/JCP.13r08765

Rushton, A., Eveleigh, G., Petherick, E. J., Heneghan, N., Bennett, R., James, G., & Wright, C. (2012). Physiotherapy rehabilitation following lumbar spinal fusion: a systematic review and meta-analysis of randomised controlled trials. *BMJ Open, 2*(4), e000829. doi:10.1136/bmjopen-2012-000829

Sala, G., Tatlidil, K. S., & Gobet, F. (2018). Video game training does not enhance cognitive ability: A comprehensive meta-analytic investigation. *Psychological Bulletin, 144*(2), 111-139. doi:10.1037/bul0000139

Salazar, A. P., Pinto, C., Ruschel, M., J, V., Figueiro, B., Lukrafka, J. L., & Pagnussat, A. S. (2019). Effectiveness of static stretching positioning on post-stroke upper-limb spasticity and mobility: systematic review with meta-analysis. *Annals of Physical and Rehabilitation Medicine, 62*(4), 274-282.

Saleem, G. T., Crasta, J. E., Slomine, B. S., Cantarero, G. L., & Suskauer, S. J. (2019). Transcranial Direct Current Stimulation in Pediatric Motor Disorders: A Systematic Review and Meta-analysis. *Archives of Physical Medicine and Rehabilitation, 100*(4), 724-738. doi:10.1016/j.apmr.2018.10.011

Schröder, J., van Criekinge, T., Embrechts, E., Celis, X., Van Schuppen, J., Truijen, S., & Saeys, W. (2019). Combining the benefits of tele-rehabilitation and virtual reality-based balance training: A systematic review on feasibility and effectiveness. *Disability and Rehabilitation: Assistive Technology, 14*(1), 2-11. doi:10.1080/17483107.2018.1503738

Shi, Y. X., Tian, J. H., Yang, K. H., & Zhao, Y. (2011). Modified constraint-induced movement therapy versus traditional rehabilitation in patients with upper-extremity dysfunction after stroke: a systematic review and meta-analysis. *Archives of Physical Medicine and Rehabilitation, 92*(6), 972-982. doi:10.1016/j.apmr.2010.12.036

Soares de Moura, M. C. D., Hazime, F. A., Marotti Aparicio, L. V., Grecco, L. A. C., Brunoni, A. R., & Hasue, R. H. (2019). Effects of transcranial direct current stimulation (tDCS) on balance improvement: a systematic review and meta-analysis. *Somatosensory and Motor Research, 36*(2), 122-135. doi:10.1080/08990220.2019.1624517

Stanmore, E., Stubbs, B., Vancampfort, D., de Bruin, E. D., & Firth, J. (2017). The effect of active video games on cognitive functioning in clinical and non-clinical populations: A meta-analysis of randomized controlled trials. *Neuroscience and Biobehavioral Reviews, 78*, 34-43. doi:10.1016/j.neubiorev.2017.04.011

Stevenson, T., Thalman, L., Christie, H., & Poluha, W. (2012). Constraint-induced movement therapy compared to dose-matched interventions for upper-limb dysfunction in adult survivors of stroke: a systematic review with meta-analysis. *Physiotherapy, 64*(4), 397-413. doi:10.3138/ptc.2011-24

Stewart, K. C., Cauraugh, J. H., & Summers, J. J. (2006). Bilateral movement training and stroke rehabilitation: a systematic review and meta-analysis. *Journal of the Neurological Sciences, 244*(1-2), 89-95. doi:10.1016/j.jns.2006.01.005

Stretton, C. M., Mudge, S., Kayes, N. M., & McPherson, K. M. (2017). Interventions to improve real-world walking after stroke: a systematic review and meta-analysis. *Clinical Rehabilitation, 31*(3), 310-318. doi:10.1177/0269215516640863

Subramanian, S. K., & Prasanna, S. S. (2018). Virtual Reality and Noninvasive Brain Stimulation in Stroke: How Effective Is Their Combination for Upper Limb Motor Improvement?—A Meta-Analysis. *PM & R, 10*(11), 1261-1270. doi:10.1016/j.pmrj.2018.10.001

Tang, A., Tao, A., Soh, M., Tam, C., Tan, H., Thompson, J., & Eng, J. J. (2015). The effect of interventions on balance self-efficacy in the stroke population: a systematic review and meta-analysis. *Clinical Rehabilitation, 29*(12), 1168-1177. doi:10.1177/0269215515570380

Tăut, D., Pintea, S., Roovers, J.-P. W. R., Mañanas, M.-A., & Băban, A. (2017). Play seriously: Effectiveness of serious games and their features in motor rehabilitation. A meta-analysis. *Neurorehabilitation, 41*(1), 105-118. doi:10.3233/NRE-171462

Tomlinson, C. L., Patel, S., Meek, C., Herd, C. P., Clarke, C. E., Stowe, R., . . . Ives, N. (2012). Physiotherapy intervention in Parkinson's disease: systematic review and meta-analysis. *BMJ, 6*(345), e5004. doi:10.1136/bmj.e5004

Triccas, L. T., Burridge, J. H., Hughes, A. M., Pickering, R. M., Desikan, M., Rothwell, J. C., & Verheyden, G. (2016). Multiple sessions of transcranial direct current stimulation and upper extremity rehabilitation in stroke: A review and meta-analysis. *Clinical Neurophysiology, 127*(1), 946-955. doi:10.1016/j.clinph.2015.04.067

Tripette, J., Murakami, H., Ryan, K. R., Ohta, Y., & Miyachi, M. (2017). The contribution of Nintendo Wii Fit series in the field of health: a systematic review and meta-analysis. *Peerj, 5*, 50. doi:10.7717/peerj.3600

Uhrbrand, A., Stenager, E., Pedersen, M. S., & Dalgas, U. (2015). Parkinson's disease and intensive exercise therapy - a systematic review and meta-analysis of randomized controlled trials. *Journal of the Neurological Sciences, 353*(1-2), 9-19. doi: 10.1016/j.jns.2015.04.004

Valkenborghs, S. R., Callister, R., Visser, M. M., Nilsson, M., & van Vliet, P. (2019). Interventions combined with task-specific training to improve upper limb motor recovery following stroke: a systematic review with meta-analyses. *Physical Therapy Reviews, 24*(3-4), 100-117. doi:10.1080/10833196.2019.1597439

Välimäki, M., Hätönen, H. M., Lahti, M. E., Kurki, M., Hottinen, A., Metsäranta, K., . . . Adams, C. E. (2014). Virtual reality for treatment compliance for people with serious mental illness. *Cochrane Database of Systematic Reviews*(10). doi:10.1002/14651858.CD009928.pub2

van Duijnhoven, H. J. R., Heeren, A., Peters, M. A. M., Veerbeek, J. M., Kwakkel, G., Geurts, A. C. H., & Weerdesteyn, V. (2016). Effects of exercise therapy on balance capacity in chronic stroke: systematic review and meta-analysis. *Stroke,47*(10), 2603-2610. doi:10.1161/STROKEAHA.116.013839

Van Criekinge, T., Truijen, S., Schroder, J., Maebe, Z., Blanckaert, K., van der Waal, C., . . . Saeys, W. (2019). The effectiveness of trunk training on trunk control, sitting and standing balance and mobility post-stroke: a systematic review and meta-analysis [with consumer summary]. *Clinical Rehabilitation, 33*(6), 992-1002.

Vazquez, F. L., Otero, P., Garcia-Casal, J. A., Blanco, V., Torres, A. J., & Arrojo, M. (2018). Efficacy of video game-based interventions for active aging. A systematic literature review and meta-analysis. *PloS One, 13*(12), e0208192. doi:10.1371/journal.pone.0208192

Veerbeek, J. M., Langbroek-Amersfoort, A. C., van Wegen, E. E. H., Meskers, C. G. M., & Kwakkel, G. (2017). Effects of robot-assisted therapy for the upper limb after stroke: a systematic review and meta-analysis. *Neurorehabilitation and Neural Repair, 31*(2), 107-121. doi:10.1177/1545968316666957

Verweij, L., van de Korput, E., Daams, J. G., ter Riet, G., Peters, R. J. G., Engelbert, R. H. H., . . . Buurman, B. M. (2019). Effects of post-acute multidisciplinary rehabilitation including exercise in out-of-hospital settings in the aged: systematic review and meta-analysis. *Archives of Physical Medicine and Rehabilitationm 100*(3), 530-550.

Wang, X. Q., Pi, Y. L., Chen, B. L., Wang, R., Li, X., & Chen, P. J. (2016). Cognitive motor intervention for gait and balance in Parkinson's disease: systematic review and meta-analysis. *Clinical Rehabilitation, 30*(2), 134-144. doi:10.1177/0269215515578295

Yang, J. D., Liao, C. D., Huang, S. W., Tam, K. W., Liou, T. H., Lee, Y. H., . . . Chen, H. C. (2019). Effectiveness of electrical stimulation therapy in improving arm function after stroke: a systematic review and a meta-analysis of randomised controlled trials [with consumer summary]. *Clinical Rehabilitation, 33*(8), 1286-1297.

Yilmazer, C., Boccuni, L., Thijs, L., & Verheyden, G. (2019). Effectiveness of somatosensory interventions on somatosensory, motor and functional outcomes in the upper limb post-stroke: a systematic review and meta-analysis. *Neurorehabilitation, 44*(4), 459-477.

Yu, J. J., Burnett, A. F., & Sit, C. H. (2018). Motor Skill Interventions in Children With Developmental Coordination Disorder: A Systematic Review and Meta-Analysis. *Archives of Physical Medicine and Rehabilitation, 99*(10), 2076-2099. doi:10.1016/j.apmr.2017.12.009

Zhang, W. W., Speare, S., Churilov, L., Thuy, M., Donnan, G., & Bernhardt, J. (2014). Stroke rehabilitation in China: a systematic review and meta-analysis. *International Journal of Stroke, 9*(4), 494-502. doi:doi.org/10.1111/ijs.12029

Zou, L., Sasaki, J. E., Zeng, N., Wang, C., & Sun, L. (2018). A systematic review with meta-analysis of mindful exercises on rehabilitative outcomes among poststroke patients. *Archives of Physical Medicine and Rehabilitation, 99*(11), 2355-2364. doi:10.1016/j.apmr.2018.04
