# Supplementary material for: Virtual Reality in Neurorehabilitation: An Umbrella Review of Meta-Analyses
Source: J Clin Med. 2021 Apr 2;10(7):1478. doi: 10.3390/jcm10071478 (PMC8038192; doi:10.3390/jcm10071478)
Supplement: Supplementary file 1 [file jcm-10-01478-s001.zip › Supplem files/Appendix 2-Supplemental_Methods.docx]

# Methods

### Data extraction and management.

Data was extracted independently by two reviewers using a predefined extraction form. Any concerns were discussed with a third reviewer. Where any information from the reviews was unclear or missing, we contacted the review authors. Two attempts have been made. Positive responses were used to update the umbrella review with data needed. The following information was extracted, including apriori moderators:

*Meta-analysis identification data:* authors, year of publication and county of origin

*Population characteristics:* age and type of clinical condition (e.g. stroke, Cerebral Palsy, TBI)

*Intervention and control characteristics:* type of VR intervention (e.g. treadmill training in VR; various exercises delivered via gaming consoles) and control intervention (e.g. conventional therapy such as treadmill training without VR); type of VR platform used for intervention (e.g. visual screen with leg sensors and treadmill; Nintendo Wii balance-board); VR intervention time (e.g. total minutes, minutes per session and number of sessions)

*Review characteristics:* design (e.g. RCT, quasi-RCT, pre-test post-test design), total number of primary studies included in the meta-analysis, total number of participants, number of participants per intervention and control group; type of intervention (e.g. VR-based intervention in combination with conventional therapy or VR-based interventions as standalone therapy); type of control group (e.g. conventional therapy or passive control group)

*Outcomes were categorized as follows:* a) lower limb activity (e.g. mobility, ambulation function, gait, walking speed); b) balance and postural control (e.g. balance, postural control); c) upper limb, arm function and activity (e.g. grip strength, arm function, improvement of motor impairment, improvement of motor function, arm-hand activities); d) activity limitation (e.g. activities of daily living, global function, independence); e) ICF WHO Framework outcomes (e.g. participation, body structure and function, activity); f) motor function; g) cognitive functioning (e.g. overall cognition). Summary of results were synthetized and reported by outcomes.

*Statistical summaries:* outcomes and effect measure with 95% confidence intervals (CI). Each effect size measure was extracted from the review as reported by the review authors (e.g. standardized mean differences, mean differences, weighted mean differences, Hedge’s *g*, odds ratio). *P* values and heterogeneity (I^2^) were also extracted.

*Apriori moderators:* age (children vs adults vs older participants), immersion (e.g. highly immersive, moderate immersion, low immersion), type of VR platform (commercial VR vs non-commercial VR), type of rehabilitation outcome (physical vs cognitive), type of control group (conventional, placebo, waiting list)

### Assessment of methodological quality of included reviews.

#### Quality of the included reviews.

We used the AMSTAR 2 appraisal tool (Shea et al., 2017). AMSTAR 2 contains 16 items which assess the quality of systematic reviews that include RCTs and non-randomized studies. Seven items are considered critical to high quality reviews such as: protocol registration (item 2), comprehensive literature search (item 4), justification for excluded studies (item 7), risk of bias assessment (item 9), appropriateness of meta-analytical methods (item 11), consideration of risk of bias when interpreting and discussing results (item 13), assessment and implication of publication bias (item 15). Other non-critical domains concern: inclusion of the PICO (population, intervention, comparison and outcome) components (item 1), justification of the study designs that were included (item 3), study and data selection performed by two reviewers (item 5 and 6), comprehensive description of the primary studies (item 8), reports of primary studies funding sources (item 10), assessing the impact of risk of bias on the study results (item 12), assessed and discussed heterogeneity (item 14) and reporting of conflicts of interest (item 16). One review author (AV) performed quality assessment of all included meta-analysis and another two reviewers (JS and DSF) performed the assessment of a random sample of included studies and obtained good agreement.

#### Quality of the evidence in included reviews.

##### Risk of bias.

We did not reassess the risk of bias of primary studies but instead reported risk of bias according to the assessments performed by review authors, where a risk of bias assessment was performed and data could be extracted for individual studies. We also collected information about the risk of bias appraisal tool used (e.g. Cochrane risk of bias tool). For each meta-analysis we presented the raw data as provided by authors and computed percentages of risk of bias to allow for a better visual inspection of risk of bias across meta-analysis. In reporting and synthetizing the risk of biases for all included reviews we used a threshold of 75% as suggested by Pollock et al. (2016). If more than 75% of the primary studies from the reported reviews had high risk of bias we considered the reviews at high risk of bias.

##### GRADE assessment.

We assessed and reported the quality of evidence for each outcome using a modified version for systematic reviews of the GRADE approach developed by Pollock et al. (2016). The algorithm for assigning GRADE levels of evidence is based on four essential criteria such as: number of participants, risk of bias of primary studies, heterogeneity of results and methodological quality of the review.

Similar to the original GRADE approach (Ryan, 2016), each study is assigned a grade score based on the following: imprecision (number of participants), risk of bias (trial quality), inconsistency (heterogeneity) and risk of bias (review quality). Failure to meet the key criteria will result in downgrading one or two levels for each criteria, depending on how serious the risk is. For example, if the number of participants within the pooled analysis exceeds 200, no downgrading will be applied, but if the number is less than 200, we will downgrade one level and for less than 100 we will downgrade two levels. If the participants have less than 75% of risk of bias for selection (randomization) and detection (observer blinding) bias, we will not downgrade. In cases where more than 75% have increased risk of bias or if risk of bias is not reported we will downgrade one level. For heterogeneity, if I^2^ is lower than 75% there is no need for downgrading, but if it exceeds 75% or it is not reported, then it will be downgraded one level. Risk of bias (review quality) is evaluated using the first four items of AMSTAR (Shea, 2007, 2009) that concern a priori research design, search characteristics, independence of study selection, and data extraction. According to guidelines, we will not downgrade if the review meets all four criteria, but will downgrade one level if one answer is “no” or “unclear” and downgrade two levels if more than one answer is “no” or “unclear”. At the end the total number of downgrade points is computed and assign a GRADE score. Similar to the Cochrane GRADE assessment guidelines for RCTs (Ryan, 2016; Schünemann et al. 2020) the assessment will result in four categories of evidence quality: high (further research is very unlikely to change confidence in the estimate of effect), moderate (further research is likely to have an important impact on confidence in the estimate of effects and may change the estimate), low (further research is very likely to have an important impact on confidence in the estimate of effect and is likely to change the estimate) and very low (any estimate of effect is very uncertain).

### Overlapping of studies.

We calculated the corrected covered area (CCA) to account for overlapping of studies in our umbrella review using guidelines provided by Pieper et al. (2014). The CCA is calculated by:

“Dividing the frequency of repeated occurrences of the index publication in other reviews by the product of index publications and reviews, and this product is reduced by the number of index publications” (Pieper et al., 2014, p. 2014) where the index publication is the first occurrence of a primary publication. CCA score will range between 0 to 100%. A score ranging from 0 to 5 indicates a slight overlap, a score from 6 to 10 corresponds to moderate overlap, one from 11 to 15 indicates high overlap and one above 15 indicates very high overlap. Data was extracted in a separate excel sheet and included a list of all primary publications (author/s name, year of publication and title of study) on separate rows and a list of all meta-analysis included in the umbrella review. The occurrence of each primary study in each review was marked by an “x” in the excel spreadsheet.

### Data synthesis.

We produced a narrative description and synthesis of the reviews. The general characteristics of the included reviews are described in Table 1. We organized the review findings by outcomes and reported all the comparisons that were provided by review authors. For each outcome and comparison we extracted the effect size and the 95% CI. We reported the effect size estimators as reported by review authors (e.g. standardized mean differences, mean differences, weighted mean differences, Hedge’s g, odds ratio). To assess the magnitude of the effect, for standardized mean difference and Hedge’s *g* coefficients we used Cohen’s metrics where a value of between 0.20 and 0.50 indicates a small effect, one between 0.50 and 0.80 indicates a medium effect, while a value larger than 0.80 indicates a large effect size (Cohen, 1988). For mean differences and weighted mean differences, we used review authors judgements about the magnitude of results because they were in the best position to understand and evaluate the scale results and cut-off scores, given their familiarity with study-level data. For odds ratio, no estimation of the magnitude of effect was employed because each odds ratio estimates are explained by different variables and each statistical model has a different arbitrary scaling factor (Norton et al., 2018). For each comparison we did not report the exact *p* value, but the significance level reported by review authors. We extracted I^2^ as a measure of heterogeneity and interpreted the heterogeneity based on the criteria provided by the Cochrane Handbook. I^2^ values ranging from 0 to 50% correspond to low and not important heterogeneity, values ranging from 50% to 75% correspond to moderate heterogeneity and values above 75% indicate substantial heterogeneity (Deeks et al., 2011).

To assess the effectiveness of VR-based interventions, we categorized the findings using a framework that has been used within previous Cochrane overviews (e.g. Martis et al., 2018; Sheperd et al., 2018): a) effective interventions: the meta-analysis reports moderate to high-quality evidence of effectiveness; b) promising interventions (more evidence needed): the meta-analysis reports moderate-quality evidence of effectiveness, but more evidence is needed; c) ineffective or possibly harmful interventions: the meta-analysis found moderate to high-quality evidence of lack of effectiveness; d) probably ineffective or harmful interventions (more evidence needed): the meta-analysis reports moderate quality evidence suggesting lack of effectiveness for an intervention, but more evidence is needed; e) no conclusions possible due to lack of evidence: the meta-analysis found low- or very low-quality evidence, or insufficient evidence to comment on the effectiveness of an intervention, more evidence needed.

#### Intervention effects for individual outcomes

As recommended in the Cochrane guidelines (Becker and Oxamn, 2011) we chose to report our results and statistical summaries by outcomes. The following outcomes were used: a) lower limb activity (e.g. mobility, ambulation function, gait, walking speed); b) balance and postural control (e.g. balance, postural control); c) upper limb, arm function and activity (e.g. grip strength, arm function, improvement of motor impairment, improvement of motor function, arm-hand activities); d) activity limitation (e.g. activities of daily living, global function, independence); e) ICF WHO Framework outcomes (e.g. participation, body structure and function, activity); f) motor function; g) cognitive functioning (e.g. overall cognition). Summary of results were synthetized and reported by outcomes.

#### Moderator effects

We investigated the effects of moderators and organized moderator findings by each moderator. We employed a similar approach of data extraction and reporting as we did for the overall effects.

#### Safety concerns - Adverse effects

We extracted and synthetized available data on adverse effects and reported the number of primary studies and meta-analyses that reported adverse effects. We also qualitatively provided a summary of findings.

# Results

### Quality of the included reviews.

With regards to AMSTAR 2 (Shea et al., 2017) we report the following methodological concerns (see Table 2). in relation to critical criteria (seven items):

1. Most of the reviews had a research question and study methods before conducting the review (73%), though only eight reviews worked with a pre-registered protocol (20%) and three reviews (7%) did not.
2. Most of the reviews did not perform a comprehensive literature search (93%), one (2%) partially met the criteria for a comprehensive search and only two performed an extensive search (5%). Main reasons for downgrading was the fact that most of the reviews failed to include grey literature and to justify publication restrictions.
3. Eighteen reviews (44%) justified the exclusion from the review of each potentially relevant study, while 23 reviews did not (56%).
4. Most reviews (88%) used a satisfactory technique to assess risk of bias, including random sequence generation and reporting incomplete outcome data. Two reviews assessed risk of bias from allocation concealment and blinding of participants, personnel and outcome assessors, but did not assess risk of bias from random sequence generation and reporting incomplete outcome data and one did not assess against selective reporting (5%). Three studies (7%) did not use a satisfactory technique to assess risk of bias.
5. Most studies (88%) used appropriate methods for statistical analysis and investigated causes of heterogeneity, if this was identified and five studies did not (12%).
6. Twenty-eight reviews (68%) failed to account for risk of bias in individual studies when interpreting and discussing results. Thirteen reviews (32%) included only low risk studies or if they included moderate and high-risk studies, they provided a discussion of the likely impact of risk of bias in the results.
7. Twenty-six (63%) of the reviews did not investigate publication (graphical or statistical tests) bias and discussed its impact on study results while fifteen studies (37%) assessed publication bias.

With regards to AMSTAR 2 non-critical domains (nine items):

1. Thirty reviews (73%) used PICOs criteria in the research question and for inclusion and exclusion criteria and 11 reviews (27%) did not.
2. Thirty-two studies (78%) failed to justify the reasons for including only RCTs or non-RCTs and nine explained the reasons for inclusion (22%).
3. Thirty-two studies (78%) performed study selection in duplicate while nine (22%) did not.
4. Twenty-eight reviews (68%) performed data extraction in duplicate while thirteen (32%) did not.
5. Fifteen reviews (37%) described included studies in adequate detail, thus providing relevant information on study setting and time frame for follow up compared to 24 reviews (59%) which described population, intervention, comparator and outcomes and research design but did not report study setting and time frame for follow up. Two studies (4%) did not provide enough detail.
6. Most reviews (95%) failed to report on the sources of funding for primary studies included in the review and two reported funding sources (5%).
7. Most reviews (83%) assessed the potential impact of risk of bias in individual studies on the results of the meta-analysis. Seven reviews (17%) accounted for risk of bias and either included only low risk of bias studies or authors performed analyses to investigate possible impact of risk of bias on summary estimates of effect.
8. Twenty-seven studies (66%) provided a satisfactory explanation and/or discussed heterogeneity if it was detected. Fourteen studies (34%) did not explain or discussed heterogeneity.
9. Twenty-seven reviews (66%) reported any potential sources of conflict of interest, including any funding they received for conducting the review. Fourteen reviews (34%) failed to report potential sources of conflict, or if they did, the explanation was not satisfactory, or how they managed the conflict.

### Quality of the evidence in included reviews.

#### Risk of bias.

Forty out of 41 reviews assessed risk of bias. Most reviews used the Physiotherapy Evidence Database (PEDro) Scale (21 reviews, 52%) and Cochrane’s “Risk of bias” tool (15 reviews, 36%). One used the Jadad scale (3%), one used the Joanna Briggs Institute appraisal

Tool (3%), one used Downs-Black rating scale items (3%) and one used an adapted scoring protocol (3%). In the case of nine (25%) out of 40 reviews that used a risk of bias assessment tool we were able to extract risk of bias for each primary study evaluated as the review authors reported the overall risk assessment. The results of the risk of bias assessment are reported in Supplementary Table 3.

With regards to risk of bias categories:

1. Random sequence generation (selection bias). Thirty reviews reported random sequence generation out of which nineteen studies (63%) had low risk of selection bias (more than 75% of the primary studies that were included in the pooled analysis had low risk of selection bias).
2. Allocation concealment (selection bias). Twenty-seven reviews assessed allocation concealment. Four reviews (15%) included studies at high or unclear risk of selection bias as more than 75% of the primary studies had high or unclear risk of selection bias. Six reviews (22%) included studies at low risk of selection bias as more than 75% had low risk of selection bias.
3. Blinding of participants and personnel (performance bias). Twenty-one out of 24 reviews (88%) that assessed blinding of participants and personnel included primary studies at high or unclear risk of performance bias and more than 75% of the primary studies reported high or unclear risk of performance bias.
4. Blinding of outcome assessment (detection bias). Twenty-nine reviews assessed blinding of outcome assessment. Ten reviews (34%) were at low risk of detection bias with more than 75% of the primary studies at low risk of detection bias. Only four reviews (14%) reported low risk of detection bias with more than 75% of the primary studies at high or unclear risk of detection bias.
5. Incomplete outcome data (attrition bias). Twenty-seven reviews evaluated incomplete outcome data. Eleven reviews (41%) were at low risk of attrition bias with more than 75% of the primary studies at low risk of attrition bias and two reviews (7%) were at high or unclear risk of attrition bias as more than 75% of the primary studies were at high or unclear risk of attrition bias and
6. Selective reporting (reporting bias). Fourteen reviews assessed selective reporting. Nine reviews (64%) were at low risk of reporting bias with more 75% of the primary studies at low risk of bias. Two reviews (14%) were at high or unclear risk of reporting bias with more than 75% of the primary studies at high or unclear risk of reporting bias.
7. Intention to treat (attrition bias) was assessed in 14 reviews. Five (36%) were at high risk of attrition bias as more than 75% of the participants were at high or unclear risk of bias. One (7%) was at low risk of attrition bias due to not performing intention to treat analysis with more than 75% at low risk of bias.
8. Group similarity at baseline. Fifteen reviews evaluated whether groups were similar at baseline. Thirteen reviews (87%) had similar groups at baseline with more than 75% of studies at low risk of bias.
9. Other sources of bias were assessed in three reviews. One review (33%) was at high or unclear of bias with more than 75% of participants at high or unclear risk of bias. One review (33%) was at low risk from other sources of bias with more than 75% of participants al low risk.

#### GRADE assessment.

Most of the results of comparisons that investigated the effectiveness of VR-based interventions compared to control conditions were rated as low to very low quality of evidence. Supplementary Tables 4 to 16 contain a full descriptions of GRADE assessment and reasons for downgrading for each comparison and by outcomes. Based on these results we suggest that no conclusions about the effectiveness of VR-based intervention are possible due to lack of evidence: the narrative data synthesis found low- or very low-quality evidence, or insufficient evidence to comment on the effectiveness of an intervention, more evidence needed.

### Overlapping of studies.

Using the formula provided by Pieper et al. (2014) we computed the CCA as a measure for overlapping of studies. Our total number of included primary publications (including double counting) was 601. We had 41 meta-analysis and a total number of 223 index publications. After applying the formula, we obtained a value of CCA of 0.042 which indicates a slight overlap of studies. The excel spreadsheet used by us to computer CCA can be found in the Supplementary material.

### 
